# Supplementary figures and images for: Specific Activation of T Cells by an ACE2-Based CAR-Like Receptor upon Recognition of SARS-CoV-2 Spike Protein (part 2 of 2)
Source: Int J Mol Sci. 2023 Apr 21;24(8):7641. doi: 10.3390/ijms24087641 (PMC10145580; doi:10.3390/ijms24087641)

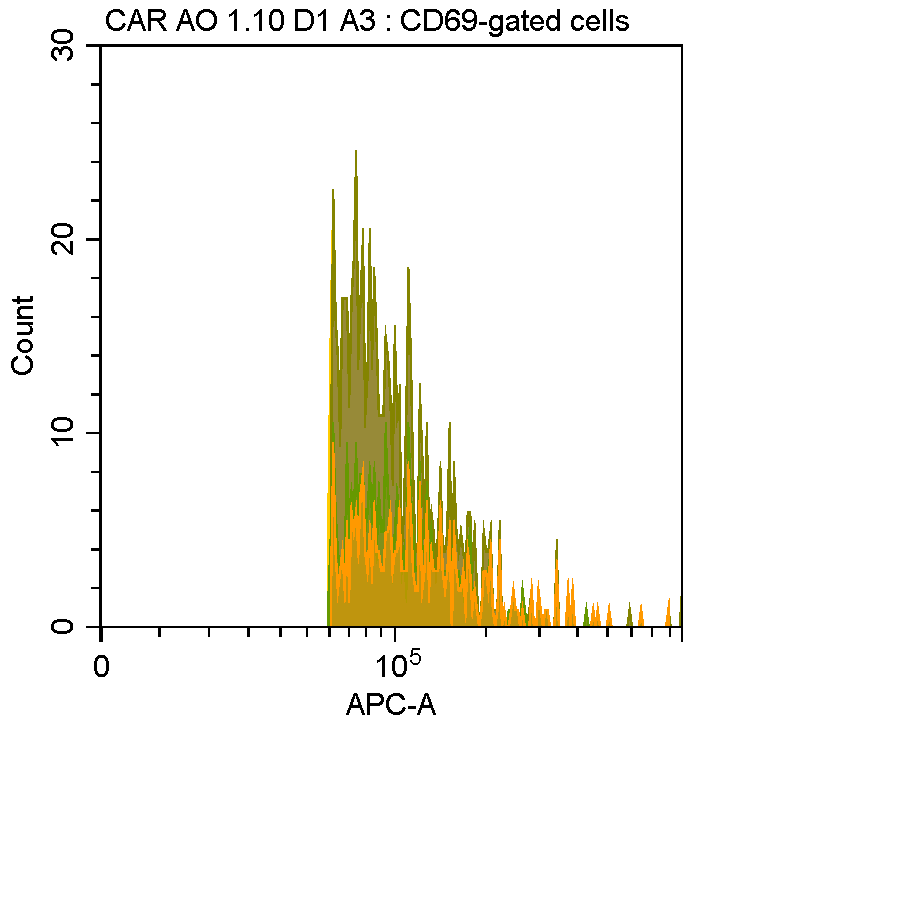

Supplement: Supplementary file 1 [file ijms-24-07641-s001.zip › Cocultures/CAR AO 1.10 D1 A3_Plot2.bmp]

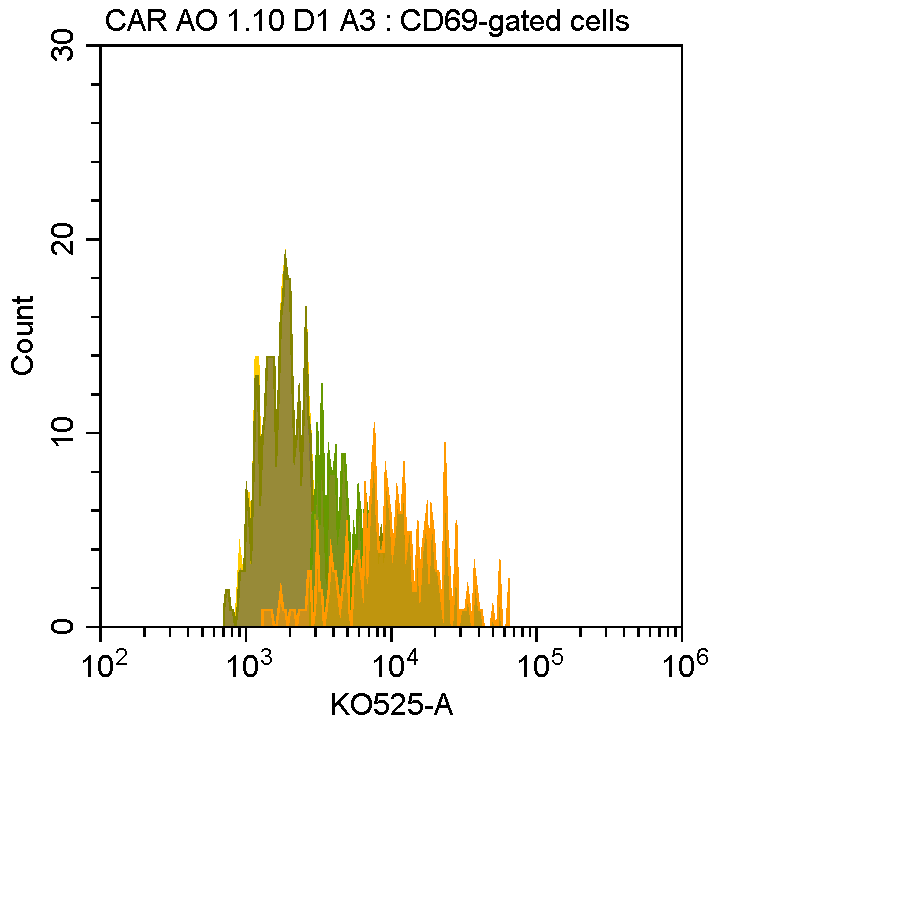

Supplement: Supplementary file 1 [file ijms-24-07641-s001.zip › Cocultures/CAR AO 1.10 D1 A3_Plot3.bmp]

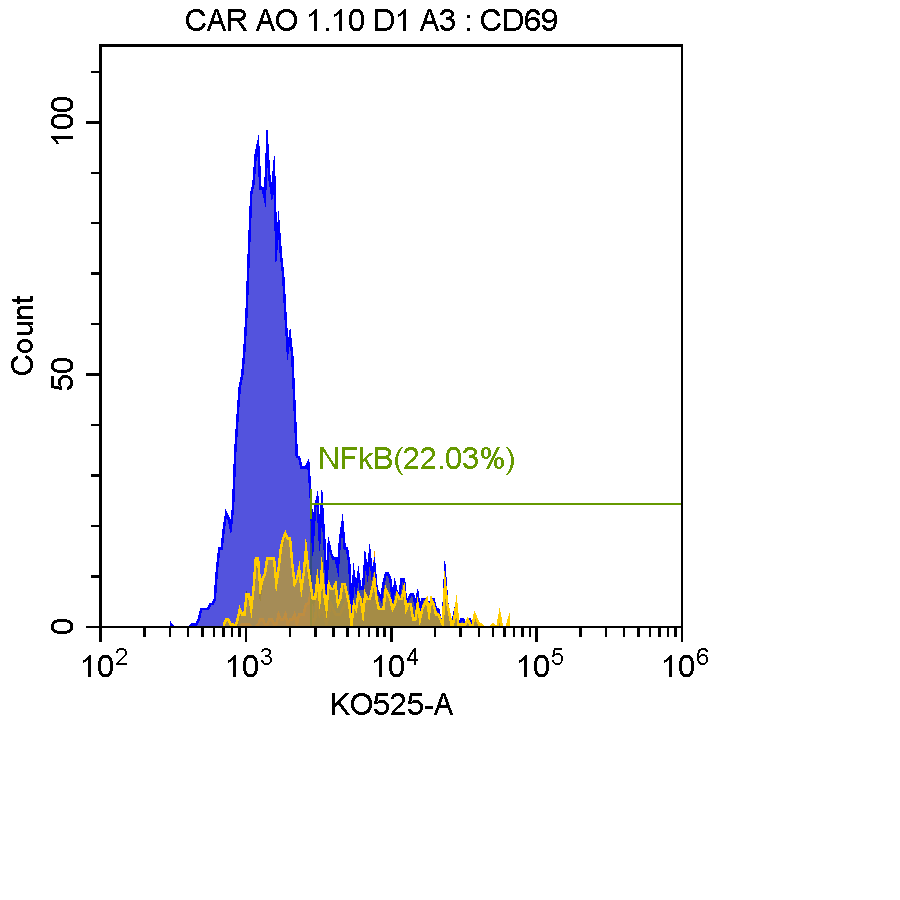

Supplement: Supplementary file 1 [file ijms-24-07641-s001.zip › Cocultures/CAR AO 1.10 D1 A3_Plot4.bmp]

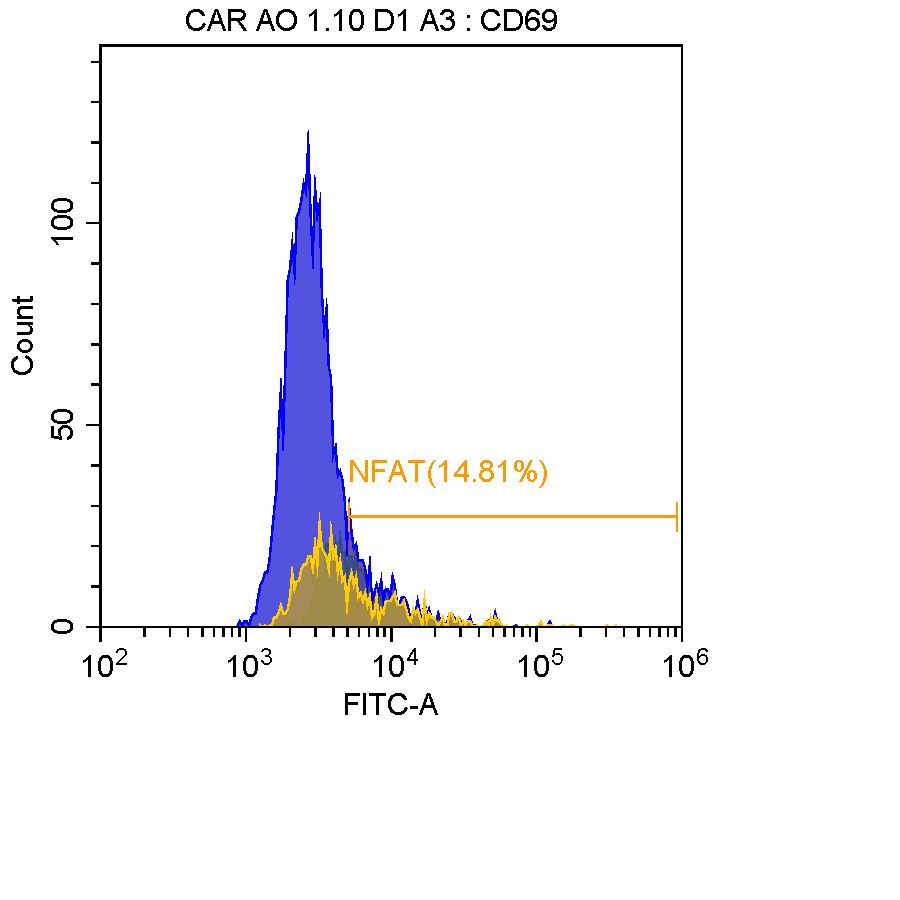

Supplement: Supplementary file 1 [file ijms-24-07641-s001.zip › Cocultures/CAR AO 1.10 D1 A3_Plot5.bmp]

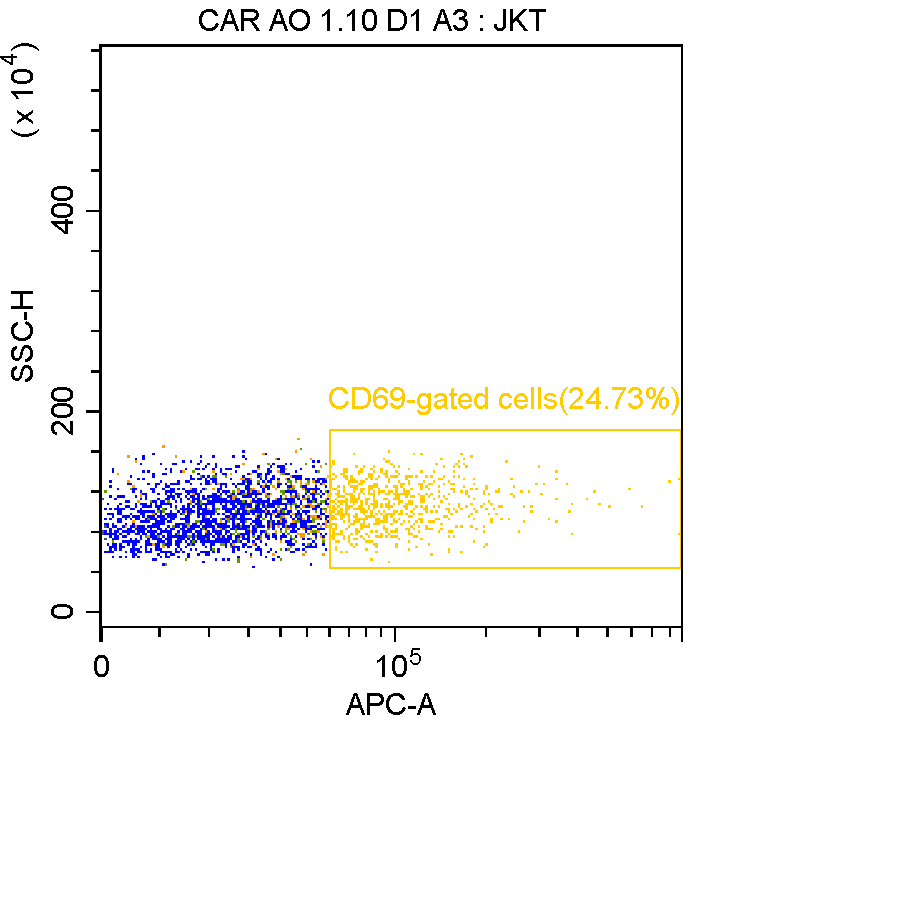

Supplement: Supplementary file 1 [file ijms-24-07641-s001.zip › Cocultures/CAR AO 1.10 D1 A3_Plot6.bmp]

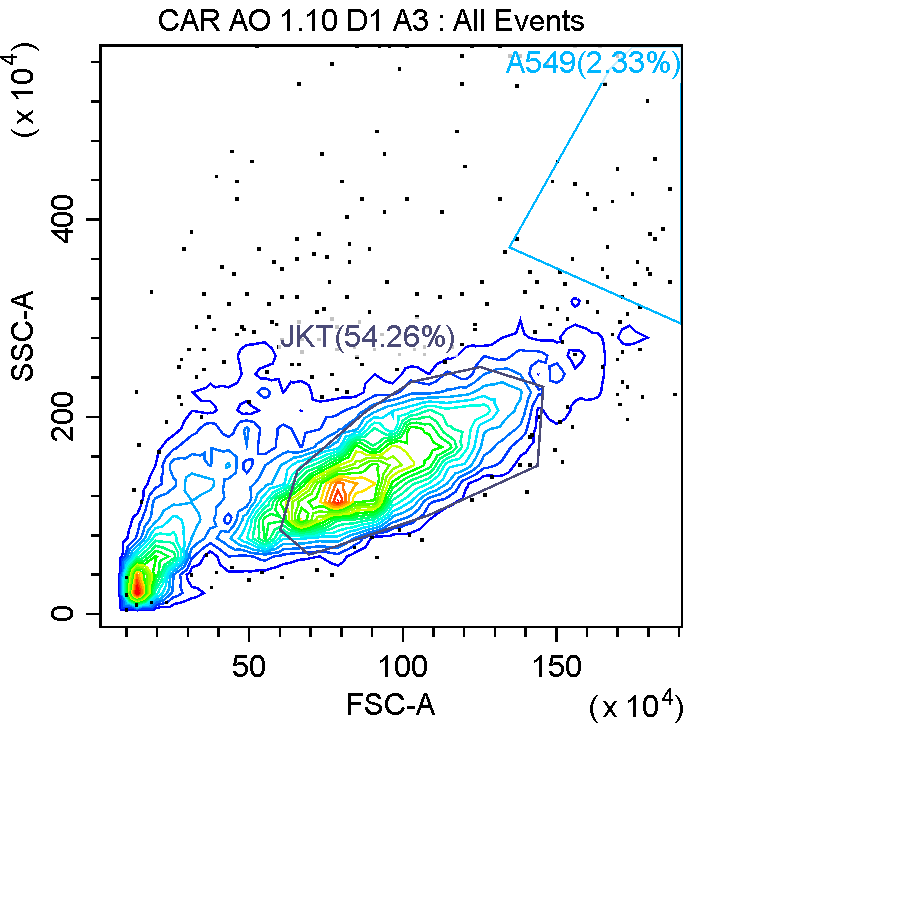

Supplement: Supplementary file 1 [file ijms-24-07641-s001.zip › Cocultures/CAR AO 1.10 D1 A3_Plot7.bmp]

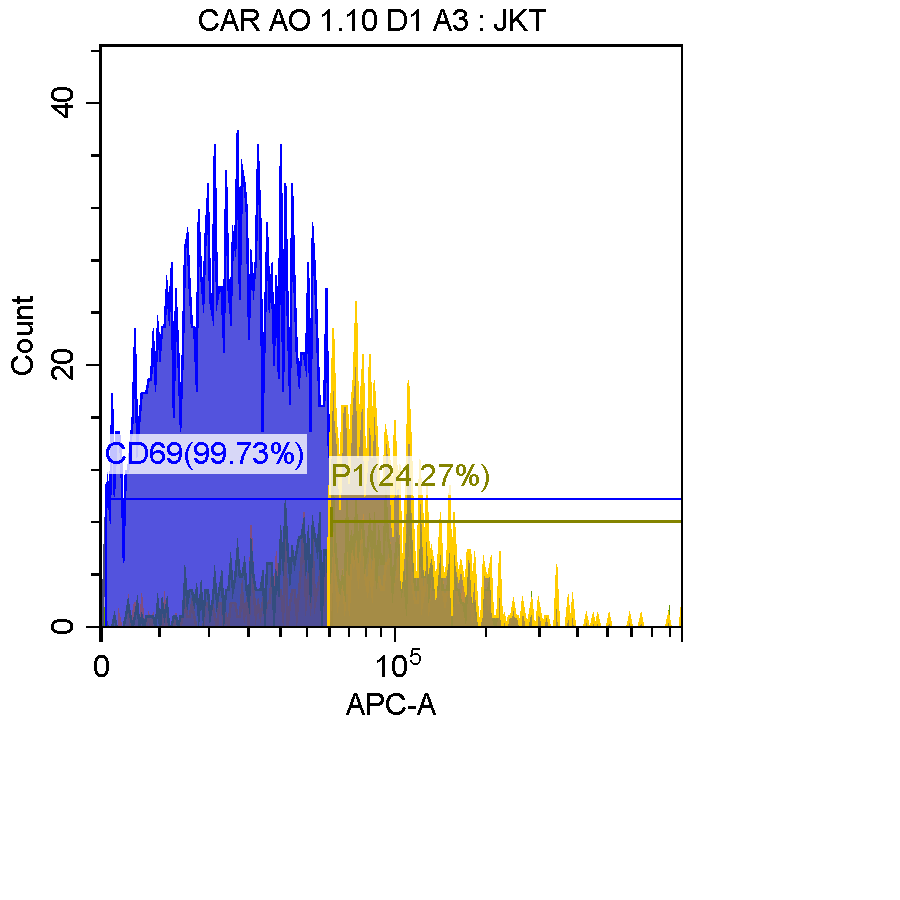

Supplement: Supplementary file 1 [file ijms-24-07641-s001.zip › Cocultures/CAR AO 1.10 D1 A3_Plot8.bmp]

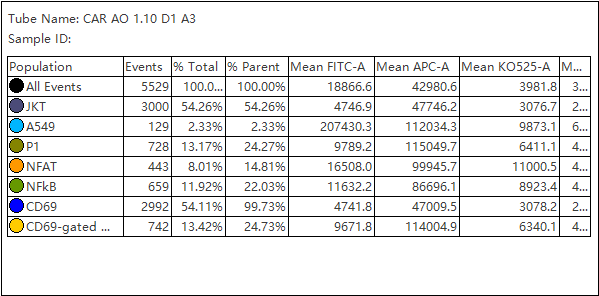

Supplement: Supplementary file 1 [file ijms-24-07641-s001.zip › Cocultures/CAR AO 1.10 D1 A3_Statistics1.bmp]

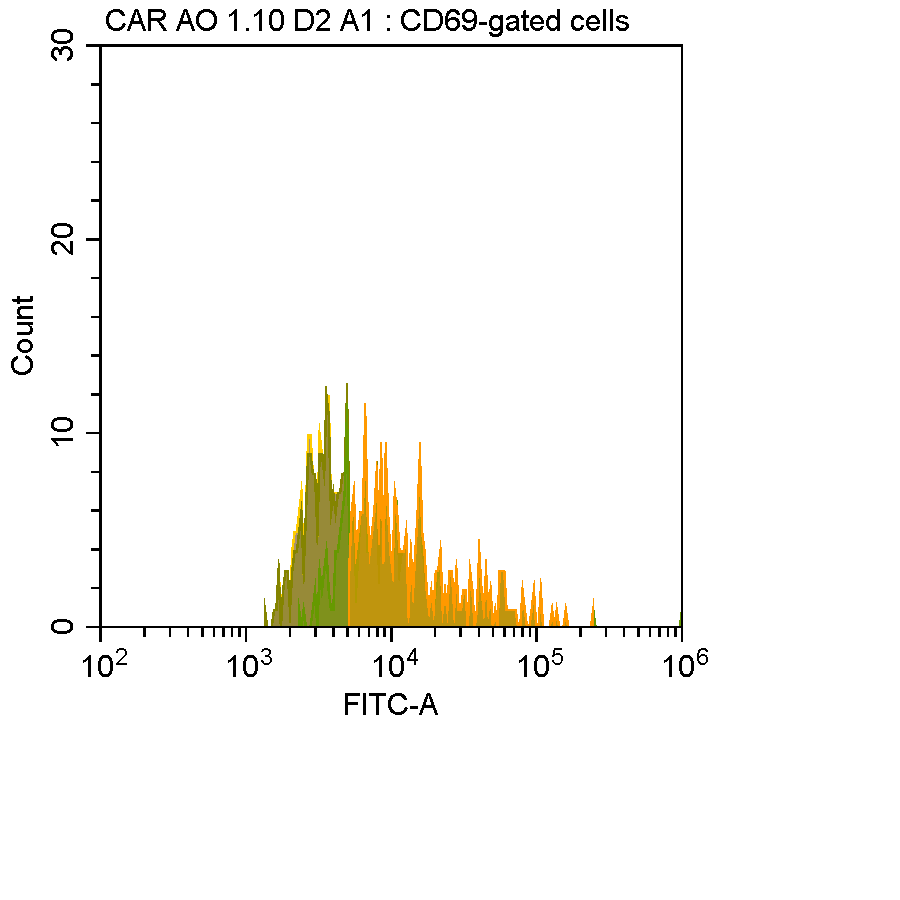

Supplement: Supplementary file 1 [file ijms-24-07641-s001.zip › Cocultures/CAR AO 1.10 D2 A1_Plot1.bmp]

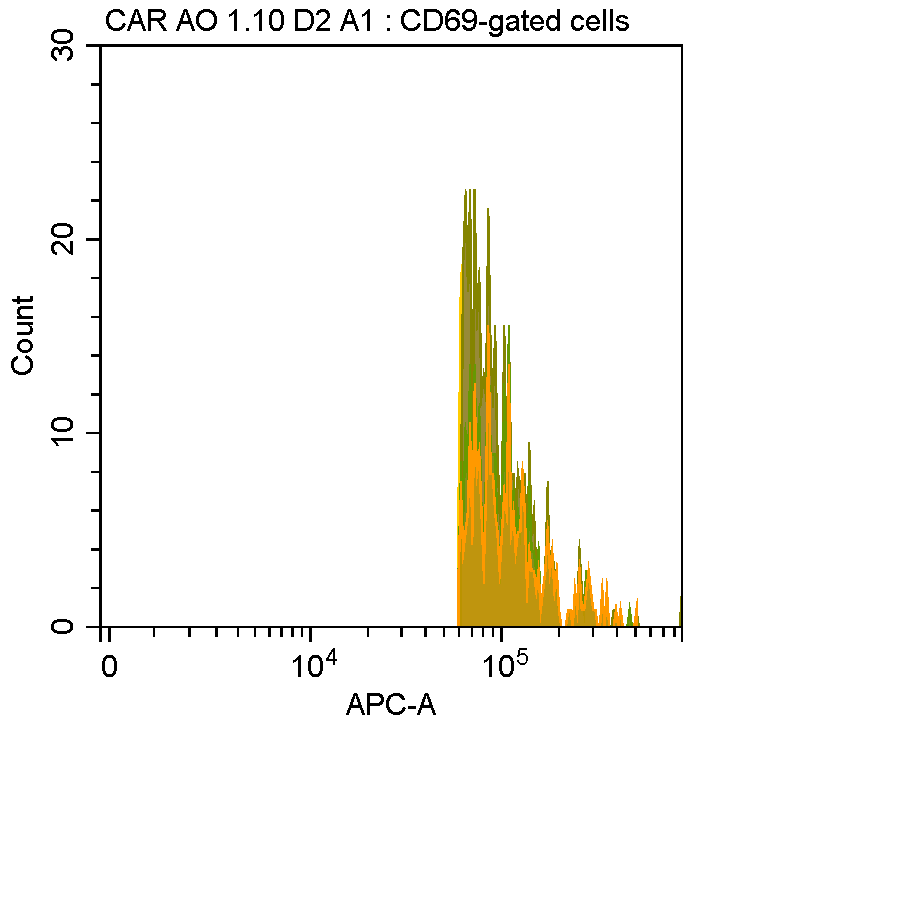

Supplement: Supplementary file 1 [file ijms-24-07641-s001.zip › Cocultures/CAR AO 1.10 D2 A1_Plot2.bmp]

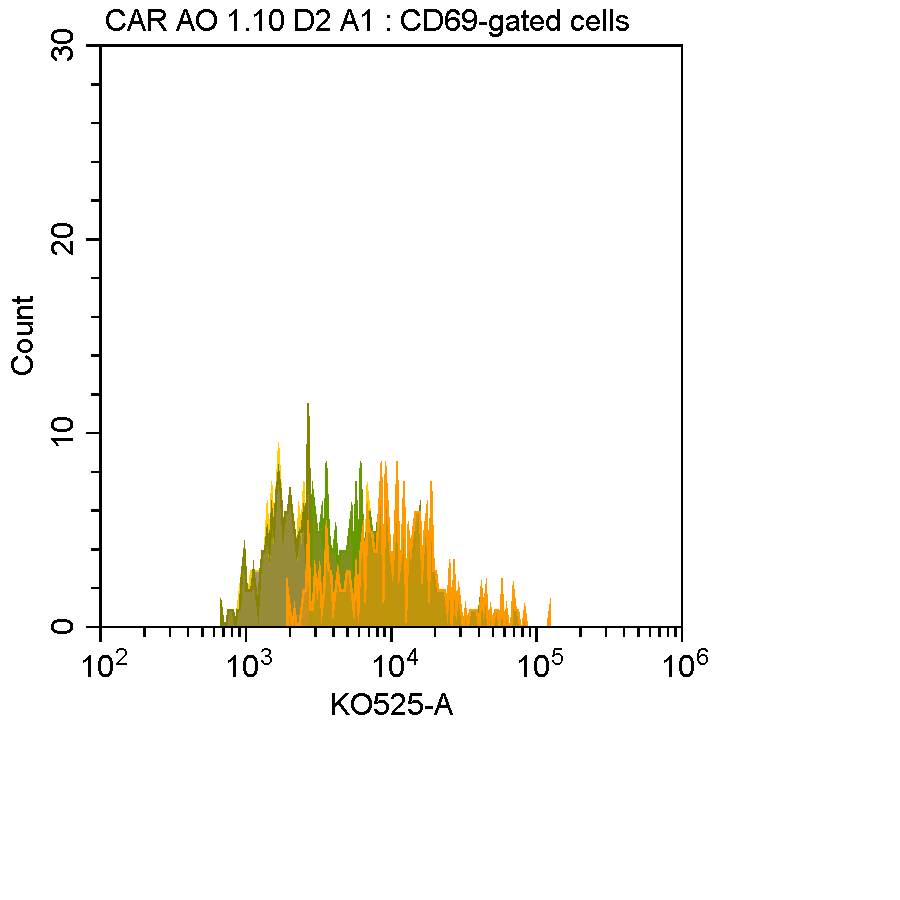

Supplement: Supplementary file 1 [file ijms-24-07641-s001.zip › Cocultures/CAR AO 1.10 D2 A1_Plot3.bmp]

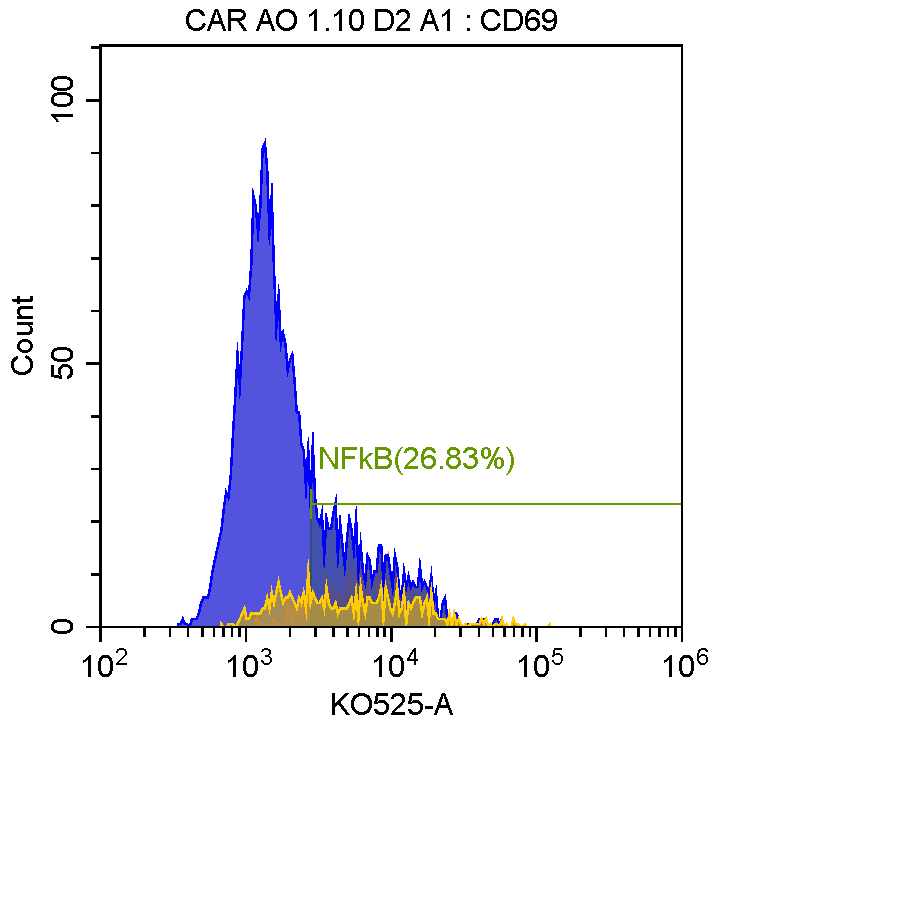

Supplement: Supplementary file 1 [file ijms-24-07641-s001.zip › Cocultures/CAR AO 1.10 D2 A1_Plot4.bmp]

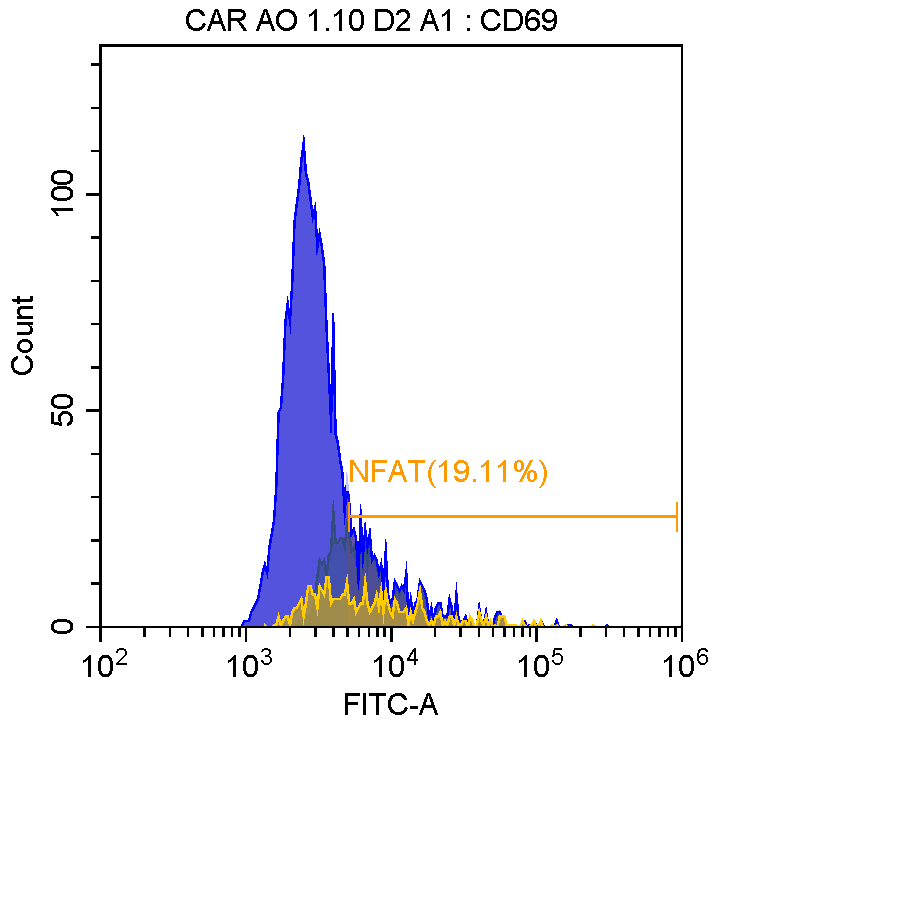

Supplement: Supplementary file 1 [file ijms-24-07641-s001.zip › Cocultures/CAR AO 1.10 D2 A1_Plot5.bmp]

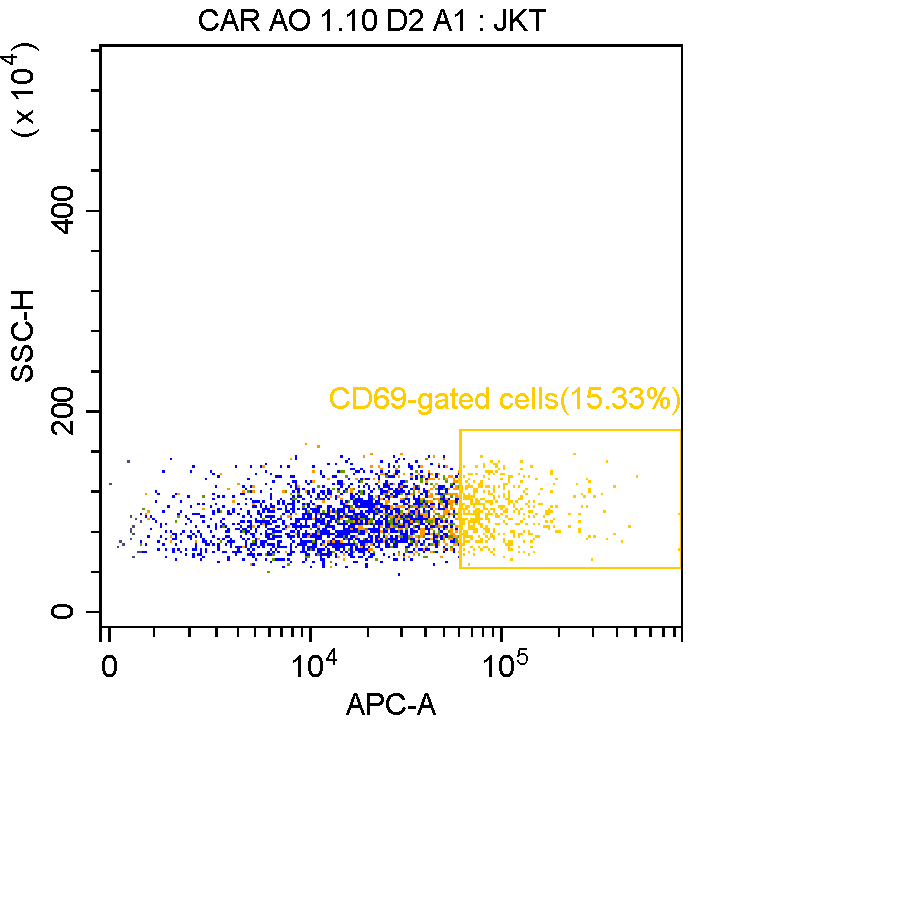

Supplement: Supplementary file 1 [file ijms-24-07641-s001.zip › Cocultures/CAR AO 1.10 D2 A1_Plot6.bmp]

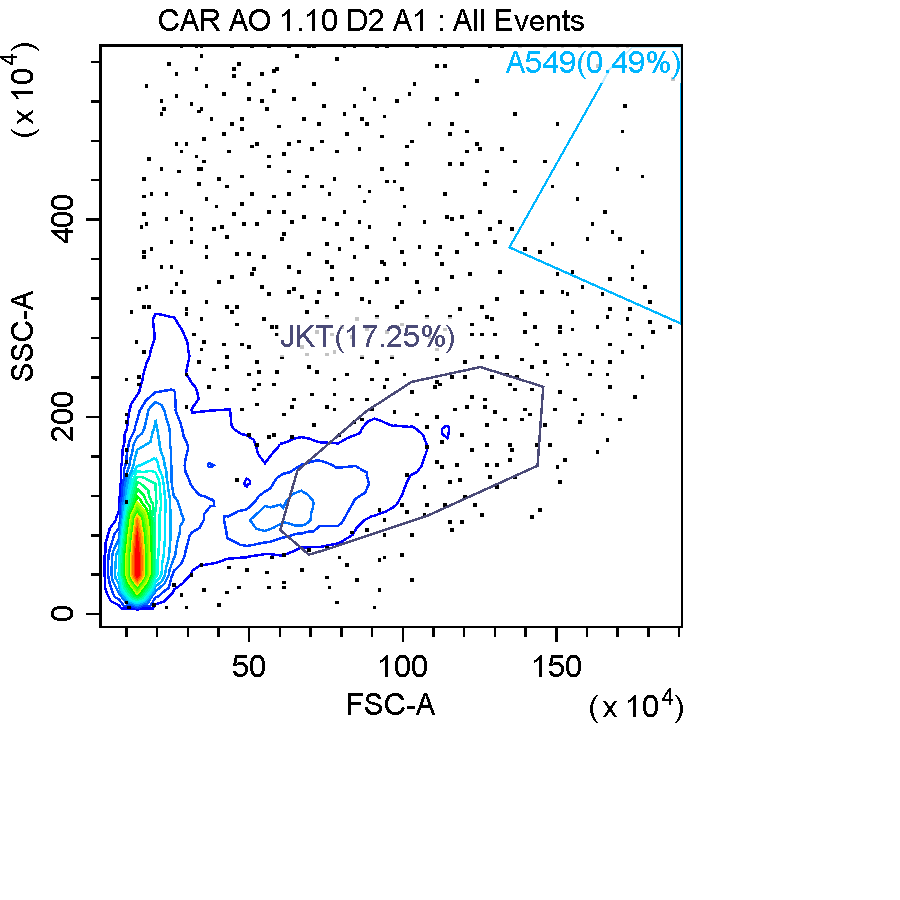

Supplement: Supplementary file 1 [file ijms-24-07641-s001.zip › Cocultures/CAR AO 1.10 D2 A1_Plot7.bmp]

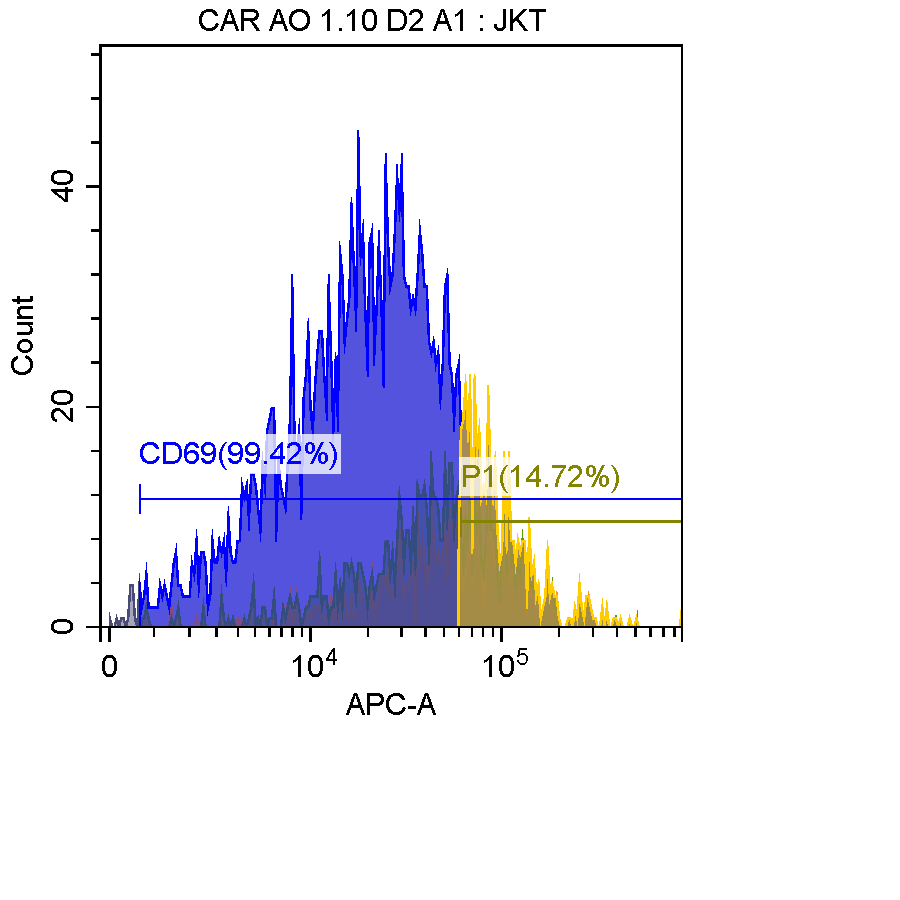

Supplement: Supplementary file 1 [file ijms-24-07641-s001.zip › Cocultures/CAR AO 1.10 D2 A1_Plot8.bmp]

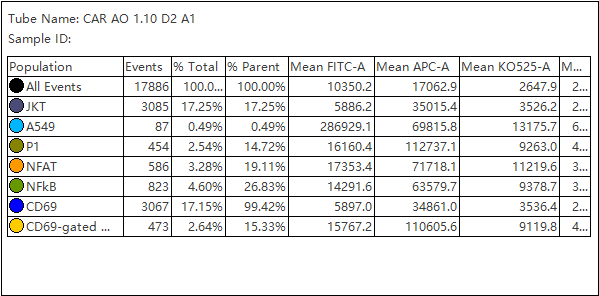

Supplement: Supplementary file 1 [file ijms-24-07641-s001.zip › Cocultures/CAR AO 1.10 D2 A1_Statistics1.bmp]

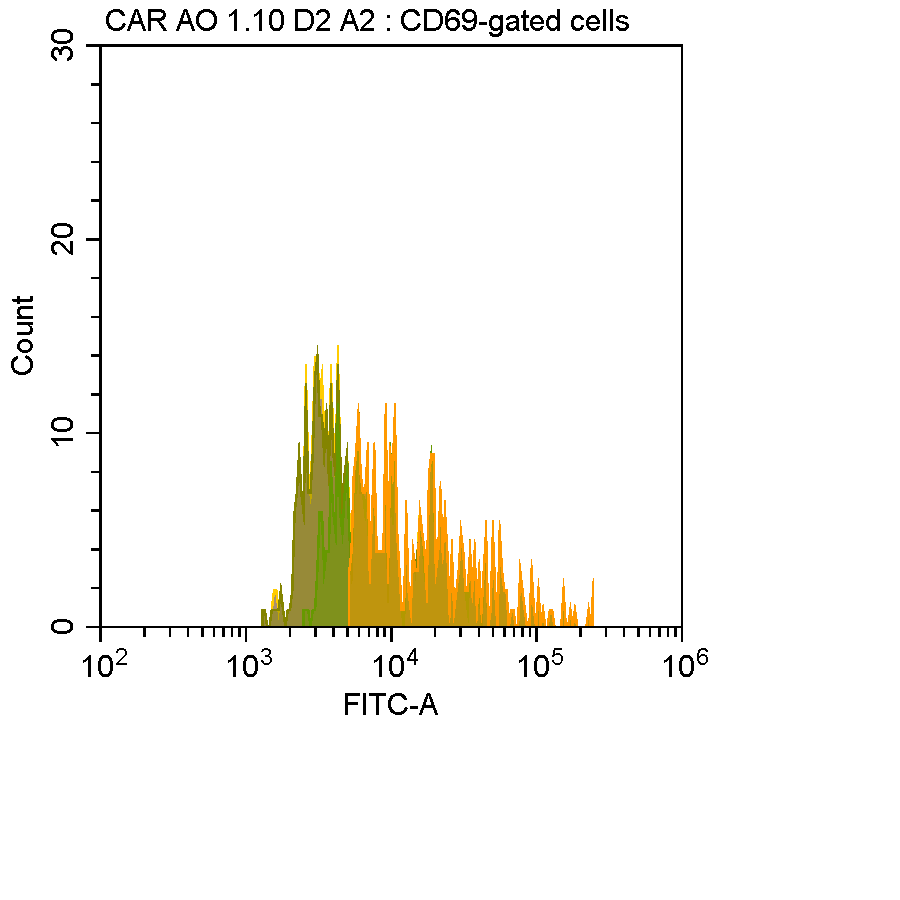

Supplement: Supplementary file 1 [file ijms-24-07641-s001.zip › Cocultures/CAR AO 1.10 D2 A2_Plot1.bmp]

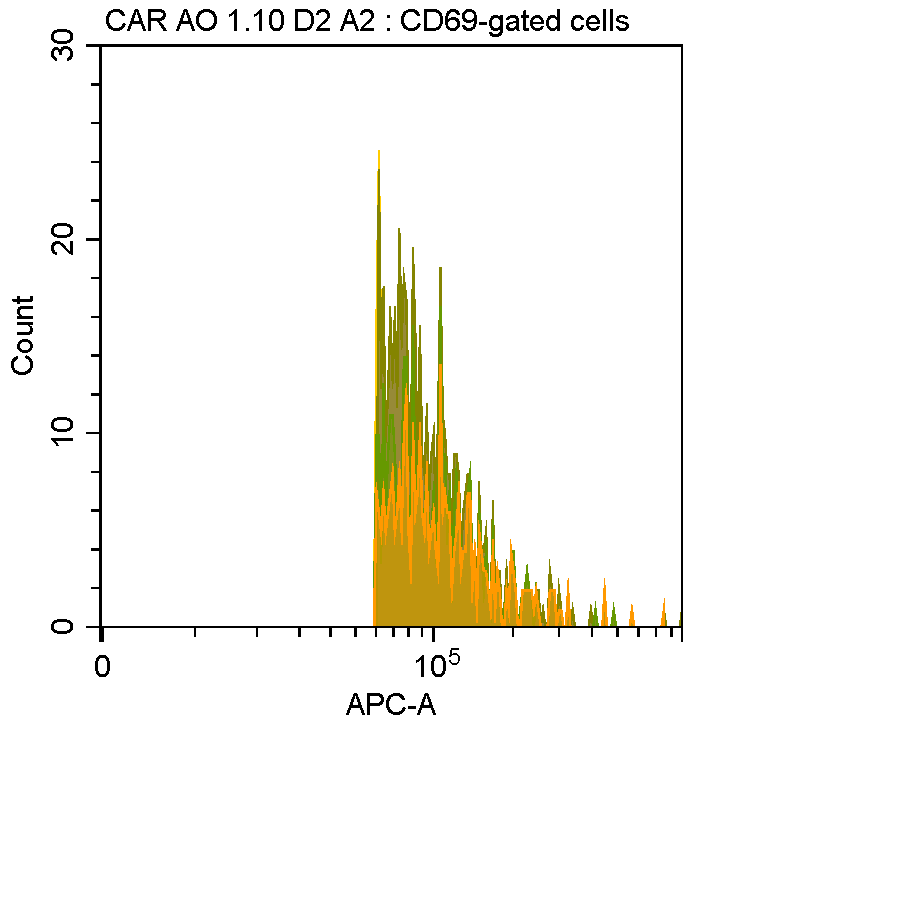

Supplement: Supplementary file 1 [file ijms-24-07641-s001.zip › Cocultures/CAR AO 1.10 D2 A2_Plot2.bmp]

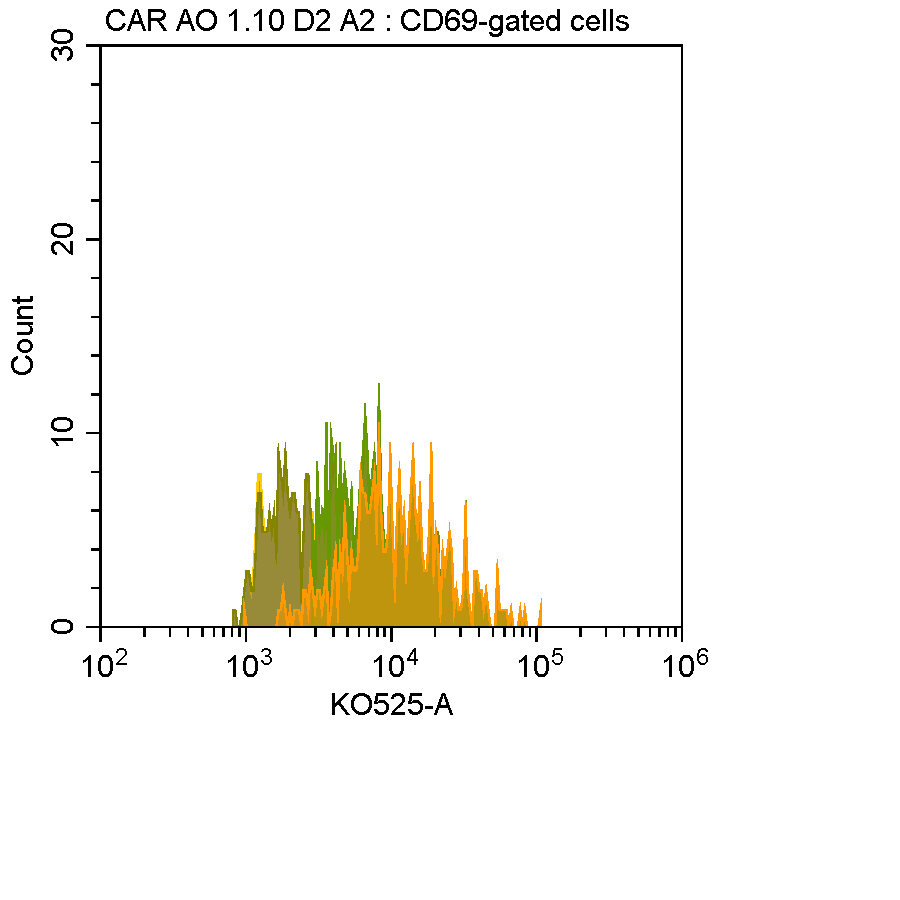

Supplement: Supplementary file 1 [file ijms-24-07641-s001.zip › Cocultures/CAR AO 1.10 D2 A2_Plot3.bmp]

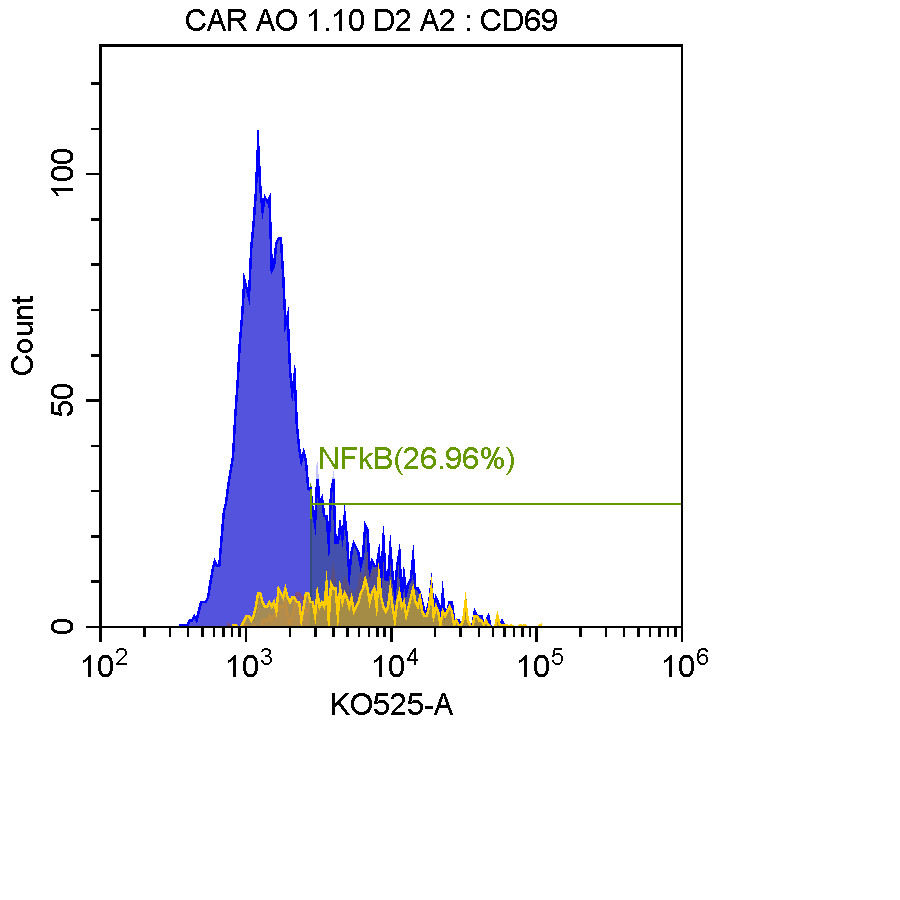

Supplement: Supplementary file 1 [file ijms-24-07641-s001.zip › Cocultures/CAR AO 1.10 D2 A2_Plot4.bmp]

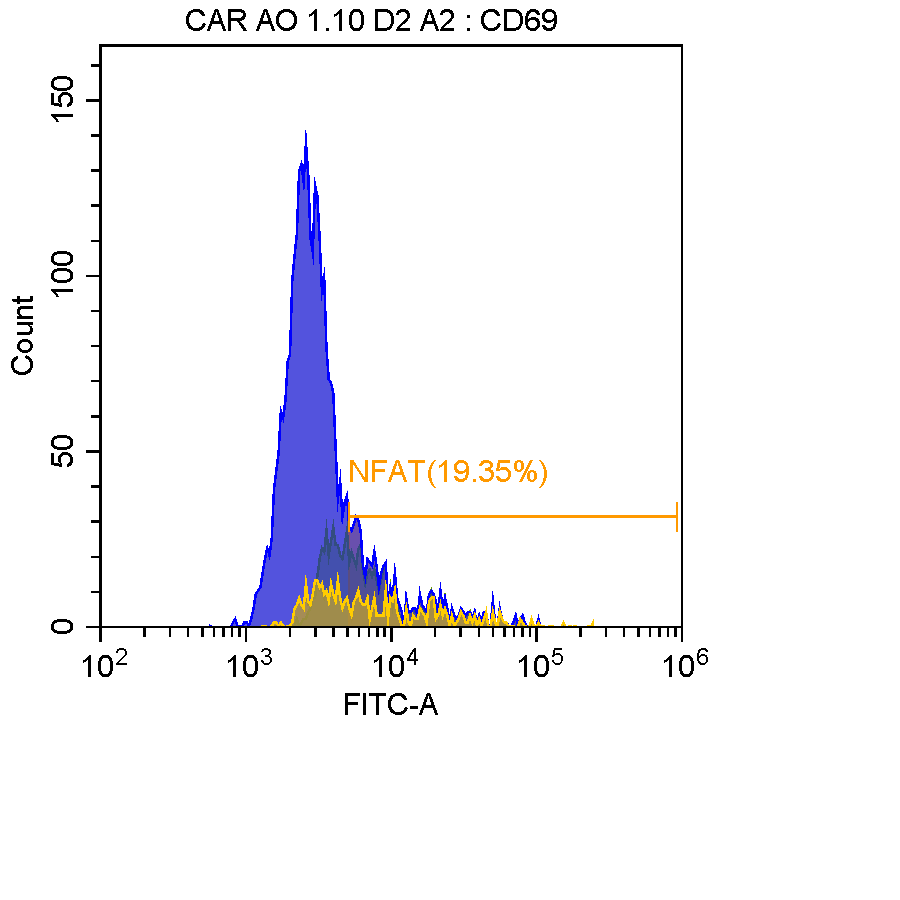

Supplement: Supplementary file 1 [file ijms-24-07641-s001.zip › Cocultures/CAR AO 1.10 D2 A2_Plot5.bmp]

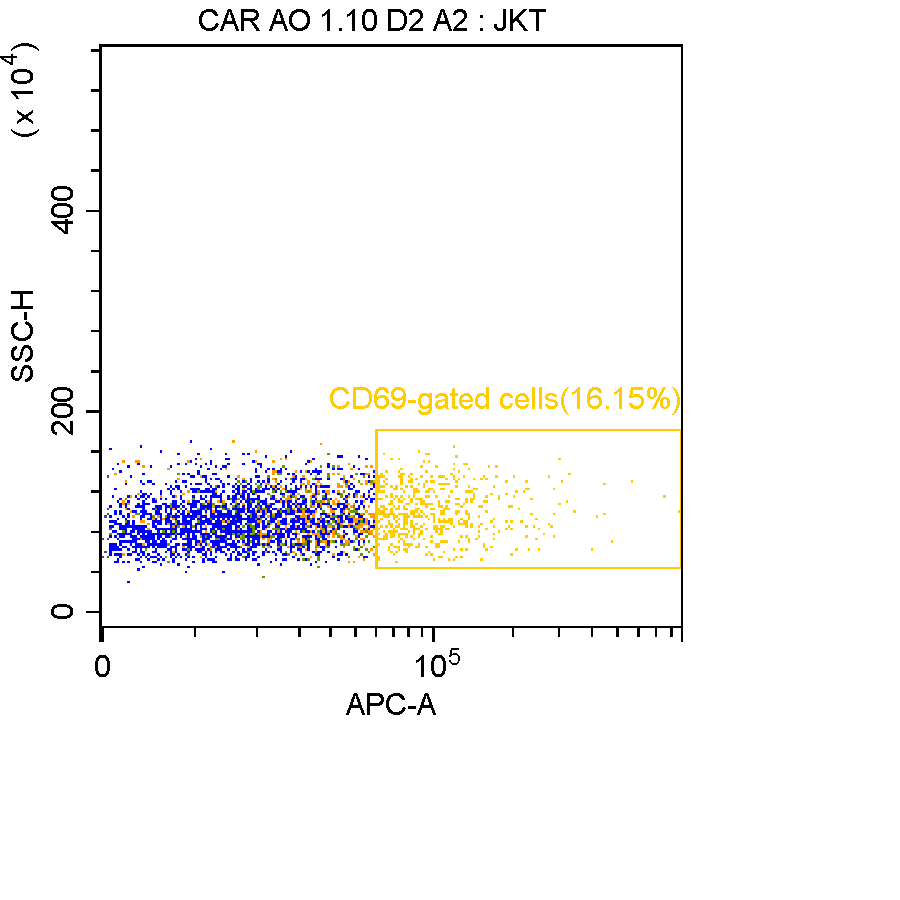

Supplement: Supplementary file 1 [file ijms-24-07641-s001.zip › Cocultures/CAR AO 1.10 D2 A2_Plot6.bmp]

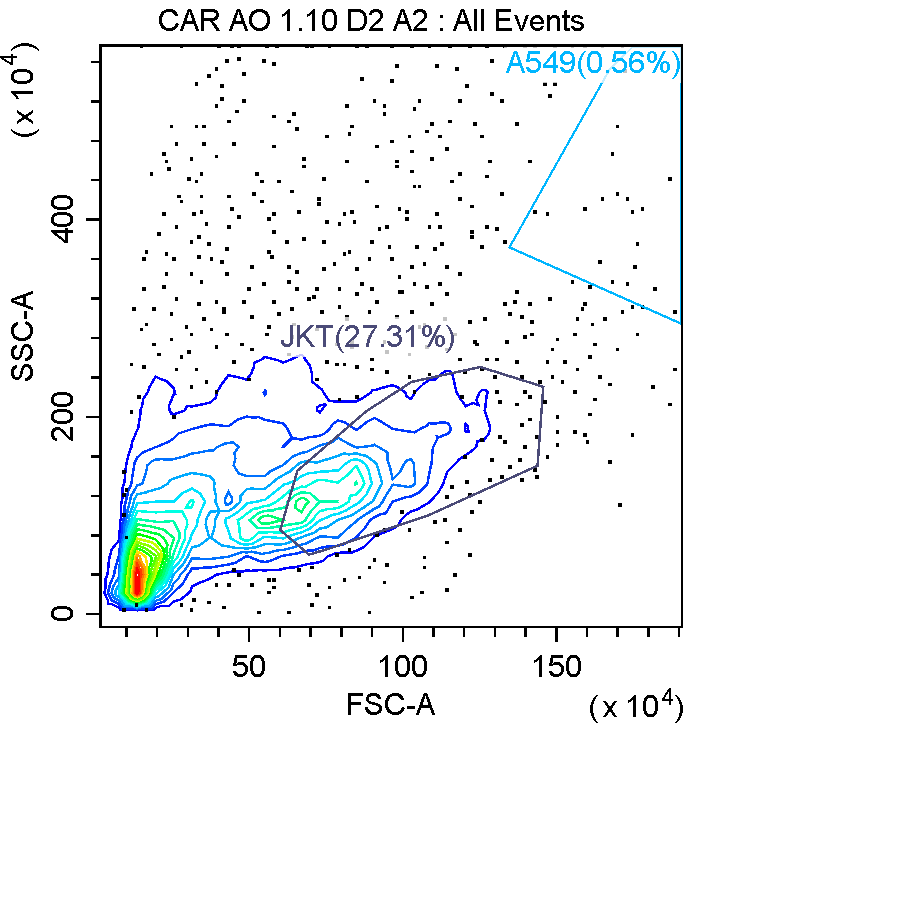

Supplement: Supplementary file 1 [file ijms-24-07641-s001.zip › Cocultures/CAR AO 1.10 D2 A2_Plot7.bmp]

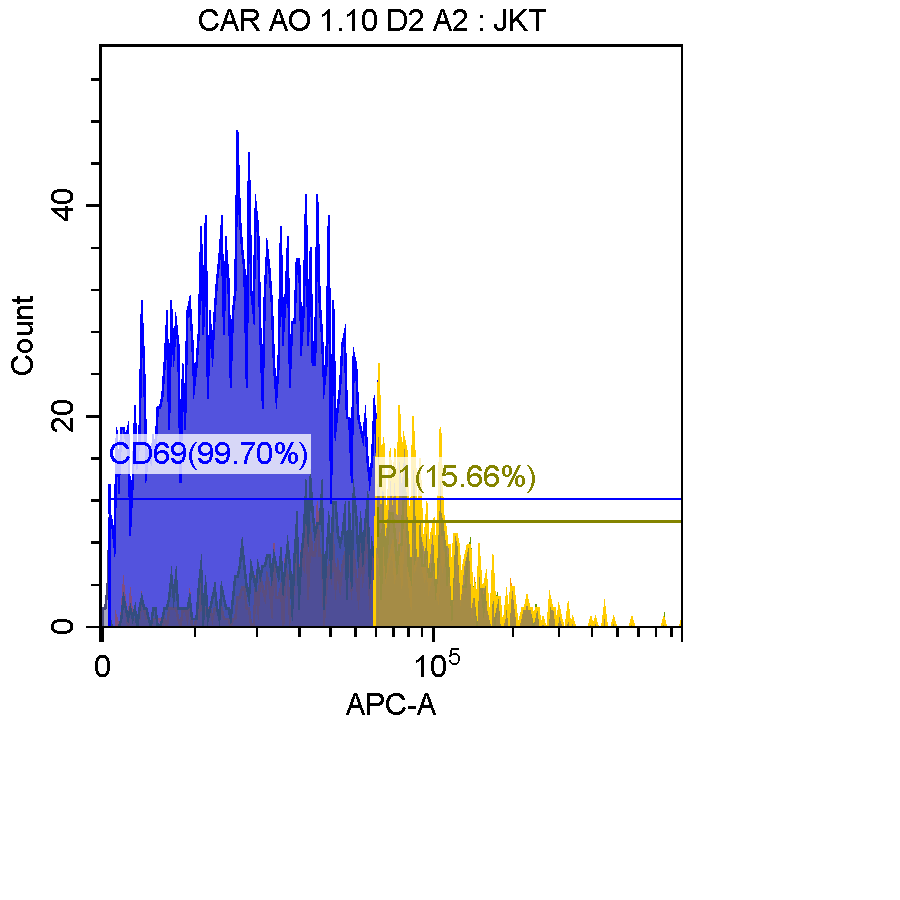

Supplement: Supplementary file 1 [file ijms-24-07641-s001.zip › Cocultures/CAR AO 1.10 D2 A2_Plot8.bmp]

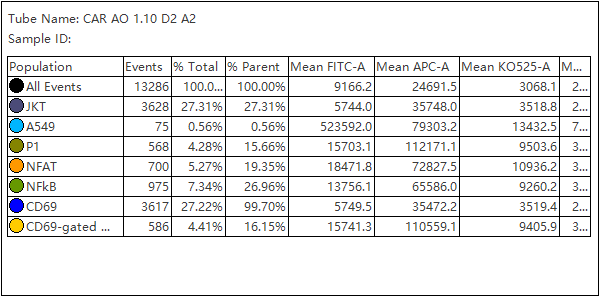

Supplement: Supplementary file 1 [file ijms-24-07641-s001.zip › Cocultures/CAR AO 1.10 D2 A2_Statistics1.bmp]

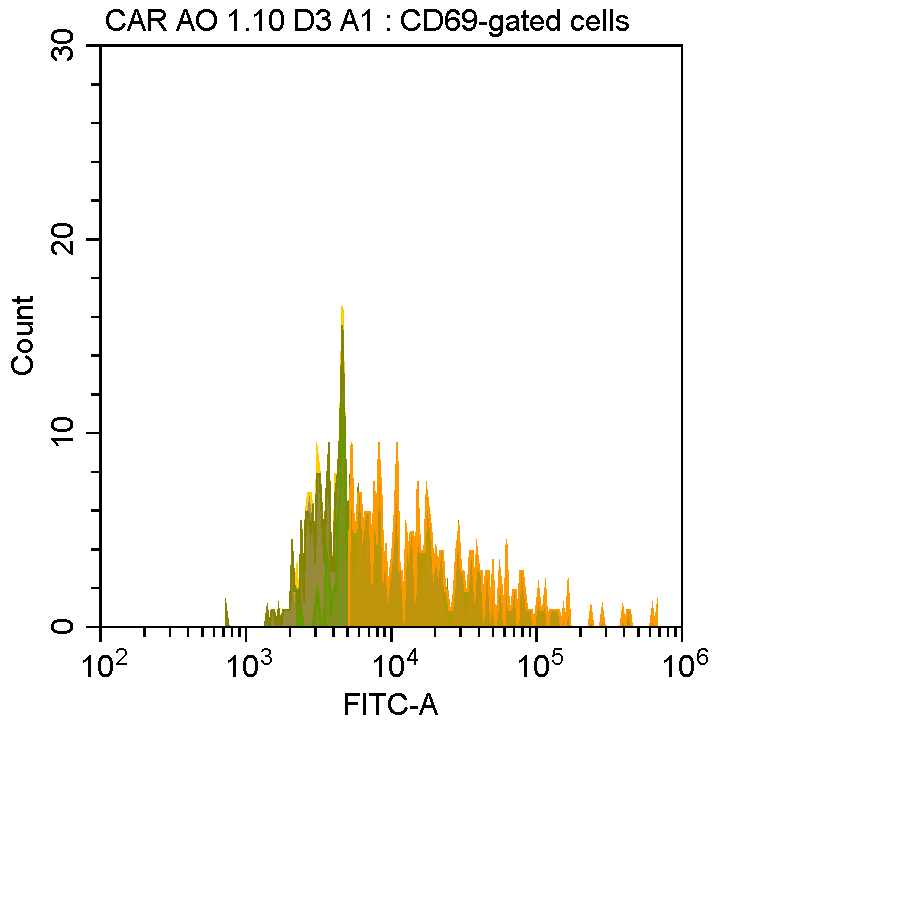

Supplement: Supplementary file 1 [file ijms-24-07641-s001.zip › Cocultures/CAR AO 1.10 D3 A1_Plot1.bmp]

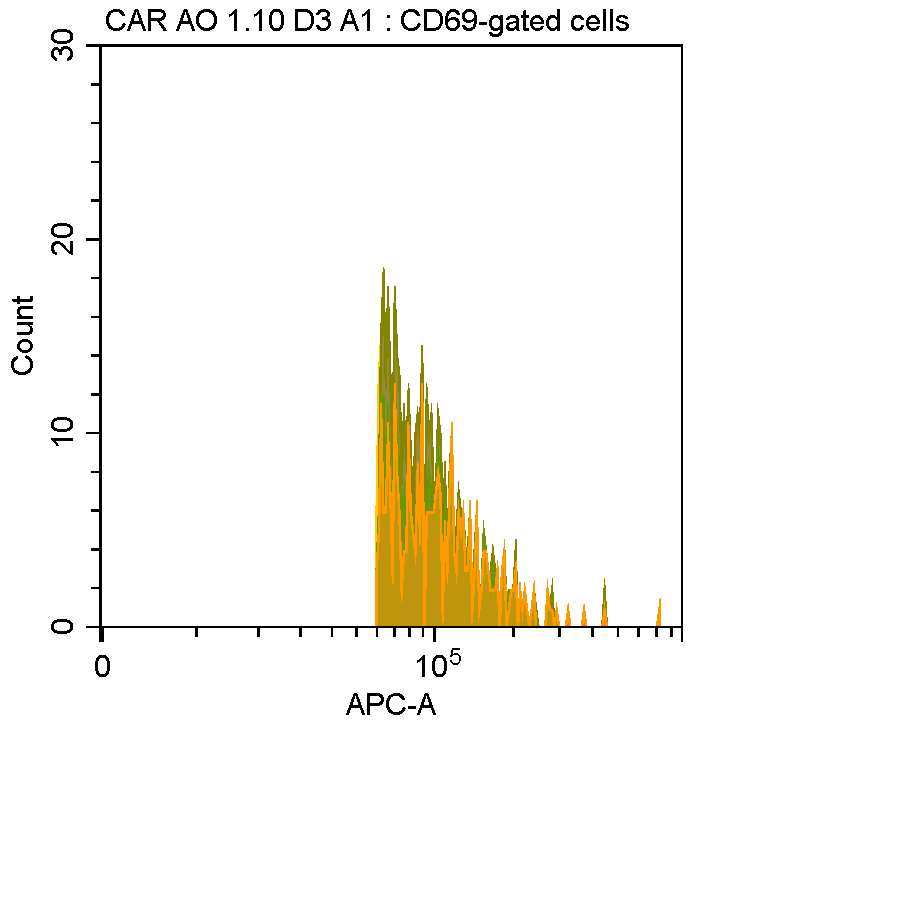

Supplement: Supplementary file 1 [file ijms-24-07641-s001.zip › Cocultures/CAR AO 1.10 D3 A1_Plot2.bmp]

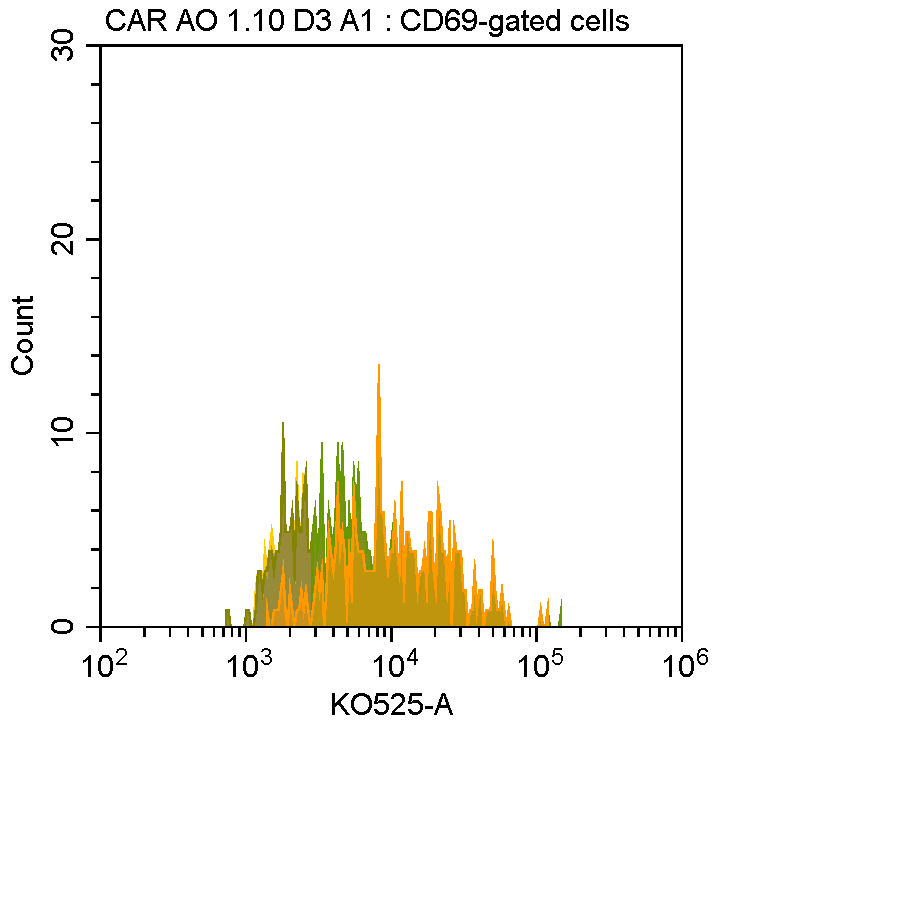

Supplement: Supplementary file 1 [file ijms-24-07641-s001.zip › Cocultures/CAR AO 1.10 D3 A1_Plot3.bmp]

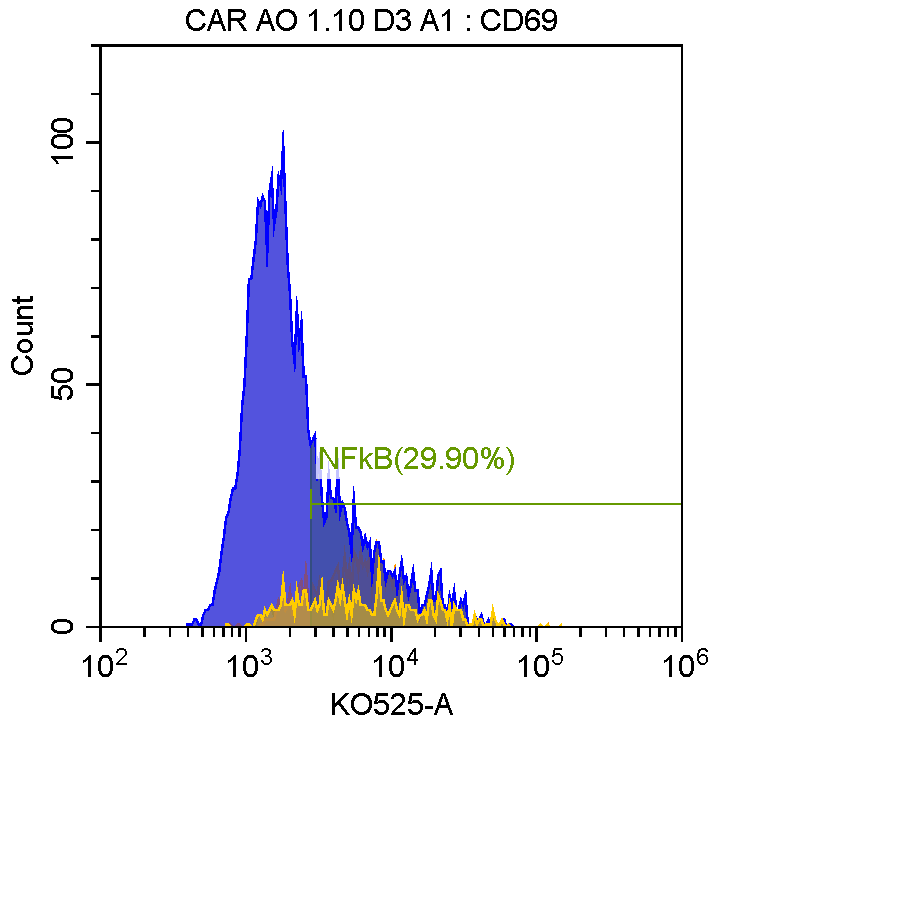

Supplement: Supplementary file 1 [file ijms-24-07641-s001.zip › Cocultures/CAR AO 1.10 D3 A1_Plot4.bmp]

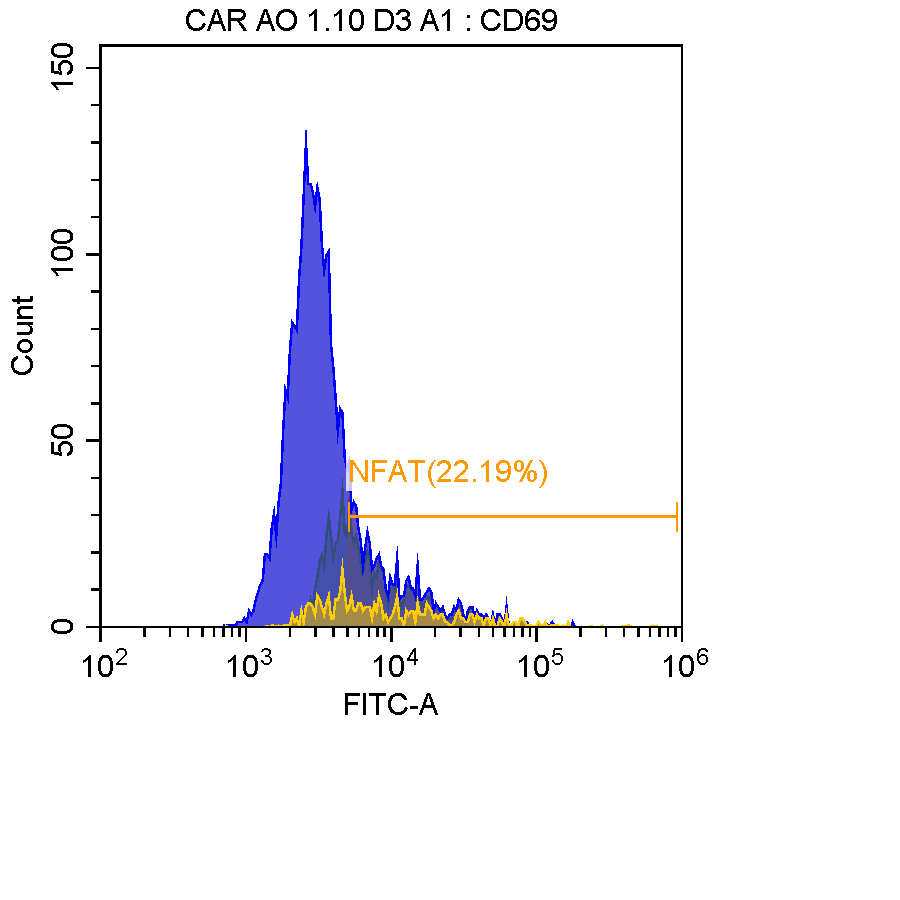

Supplement: Supplementary file 1 [file ijms-24-07641-s001.zip › Cocultures/CAR AO 1.10 D3 A1_Plot5.bmp]

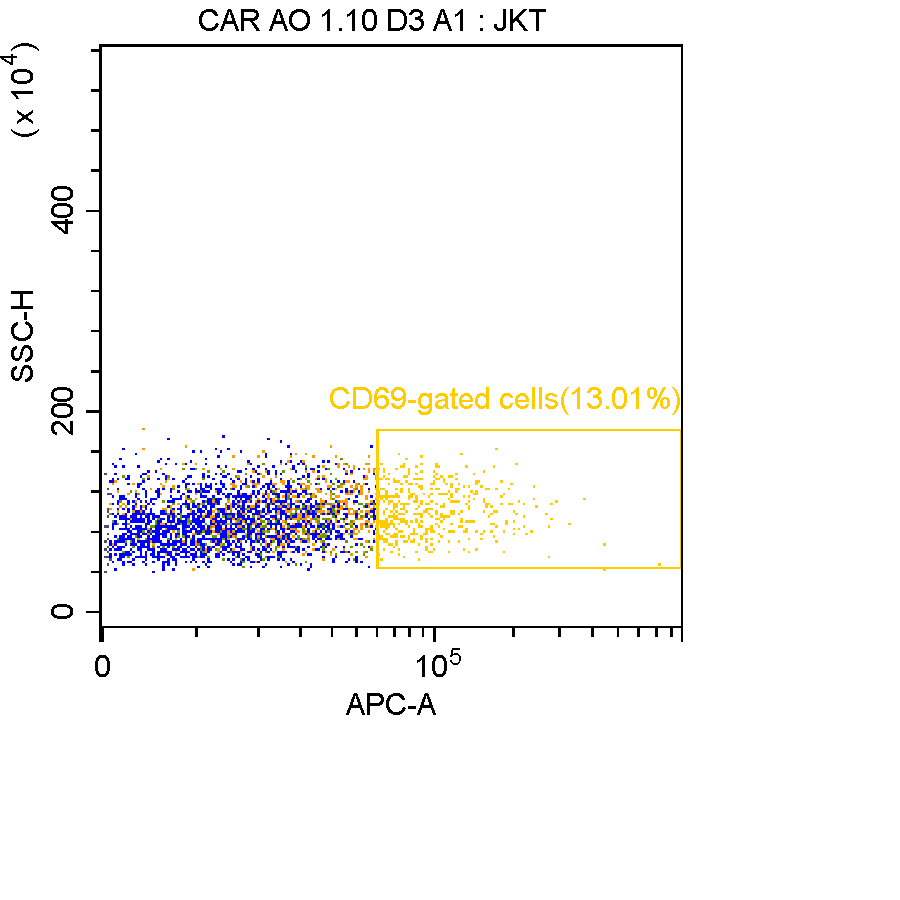

Supplement: Supplementary file 1 [file ijms-24-07641-s001.zip › Cocultures/CAR AO 1.10 D3 A1_Plot6.bmp]

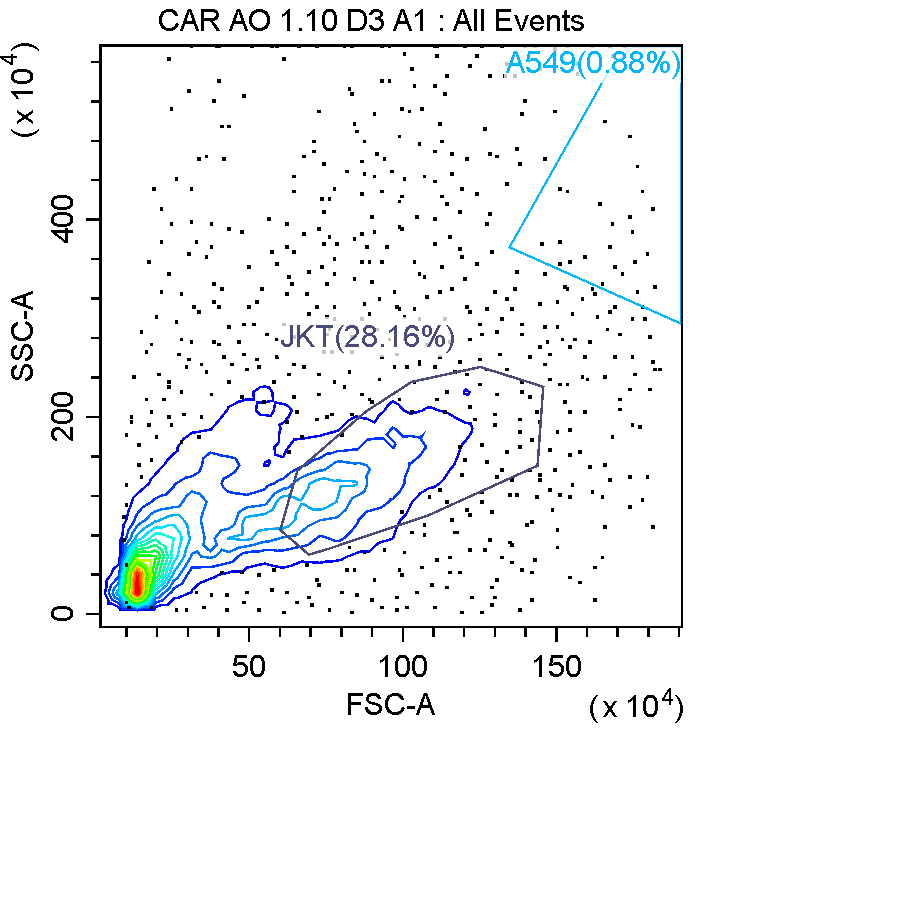

Supplement: Supplementary file 1 [file ijms-24-07641-s001.zip › Cocultures/CAR AO 1.10 D3 A1_Plot7.bmp]

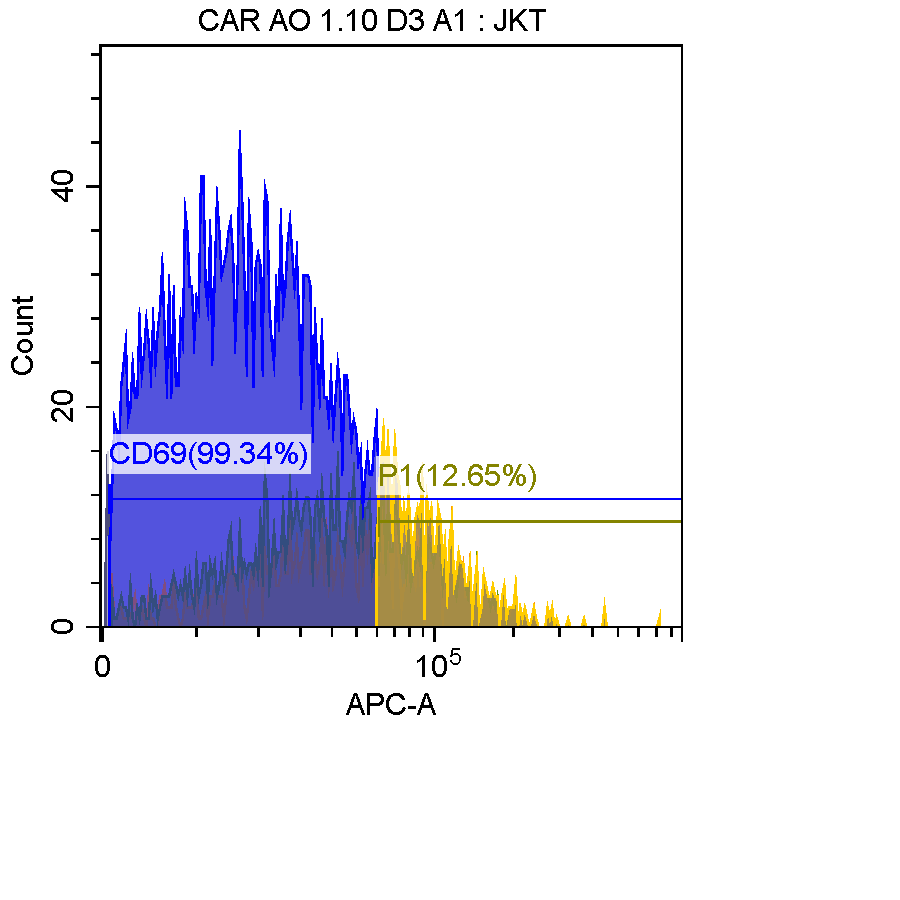

Supplement: Supplementary file 1 [file ijms-24-07641-s001.zip › Cocultures/CAR AO 1.10 D3 A1_Plot8.bmp]

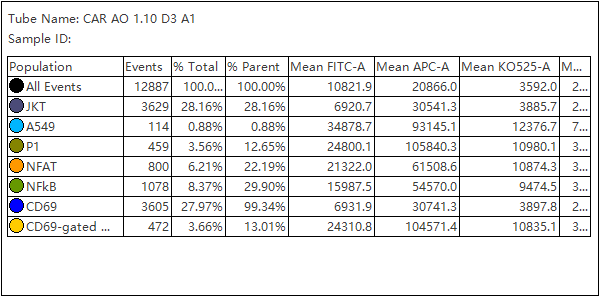

Supplement: Supplementary file 1 [file ijms-24-07641-s001.zip › Cocultures/CAR AO 1.10 D3 A1_Statistics1.bmp]

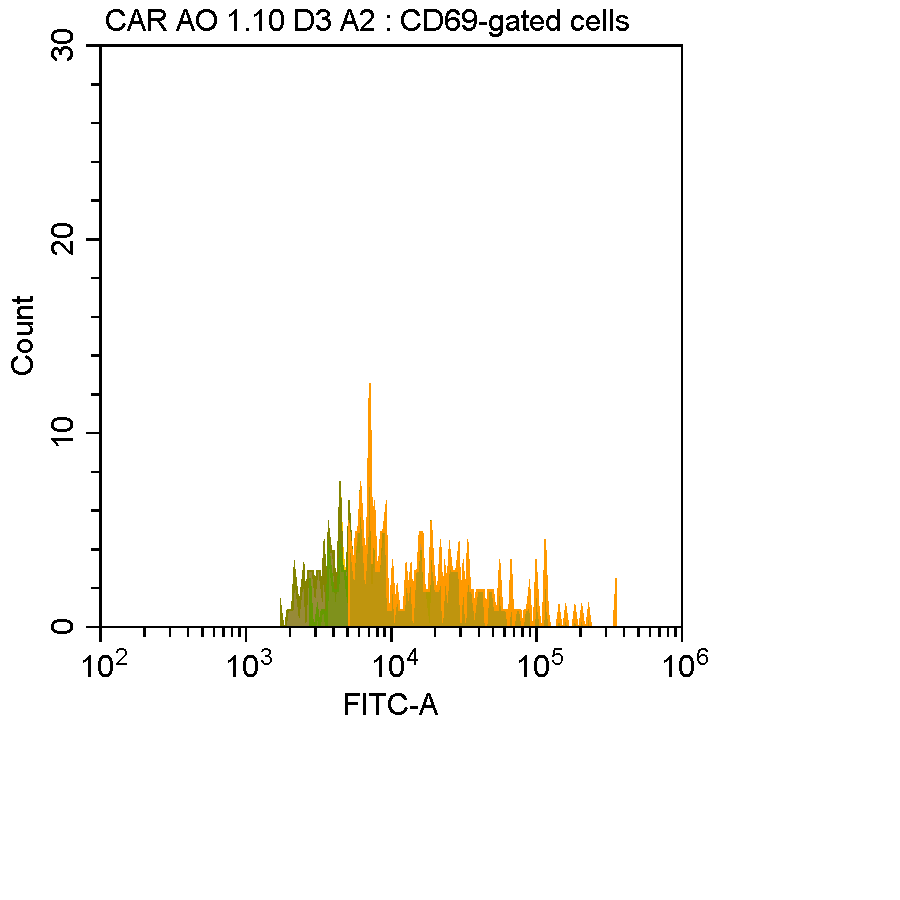

Supplement: Supplementary file 1 [file ijms-24-07641-s001.zip › Cocultures/CAR AO 1.10 D3 A2_Plot1.bmp]

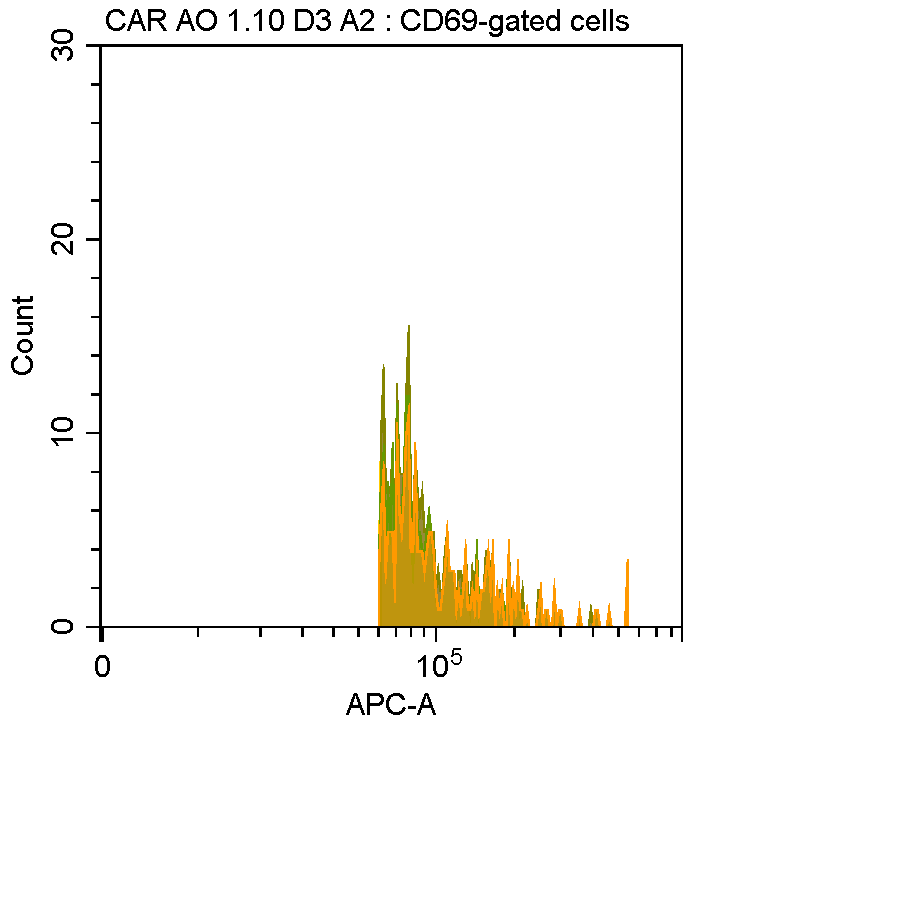

Supplement: Supplementary file 1 [file ijms-24-07641-s001.zip › Cocultures/CAR AO 1.10 D3 A2_Plot2.bmp]

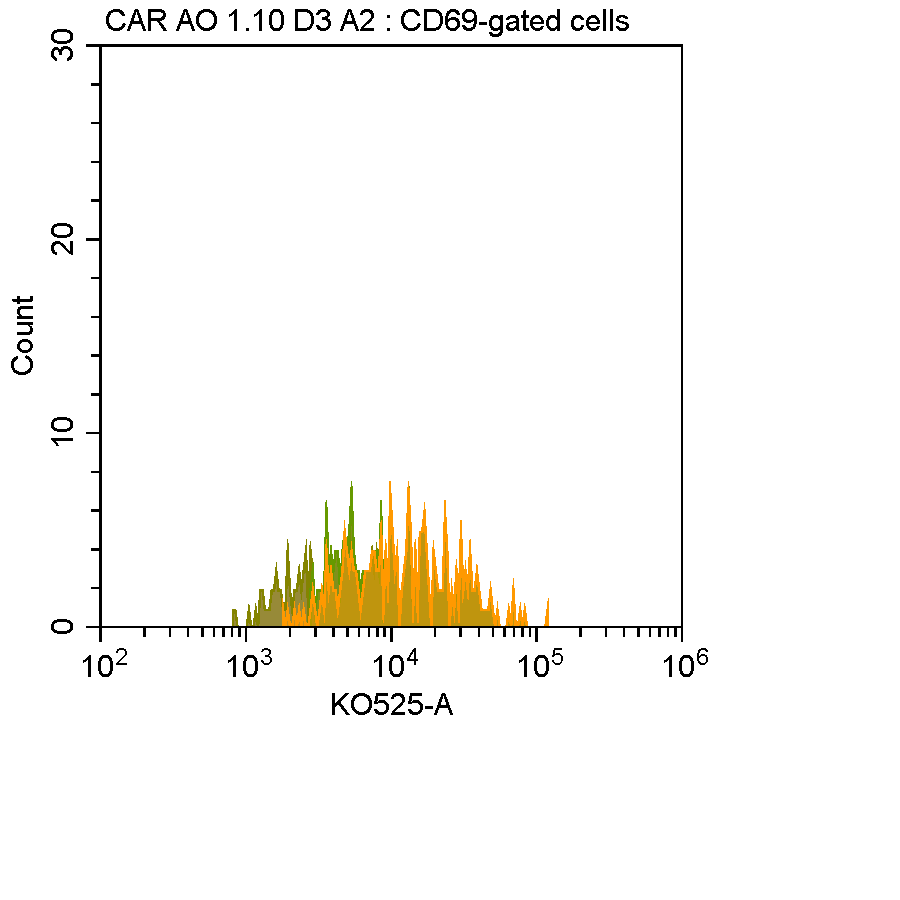

Supplement: Supplementary file 1 [file ijms-24-07641-s001.zip › Cocultures/CAR AO 1.10 D3 A2_Plot3.bmp]

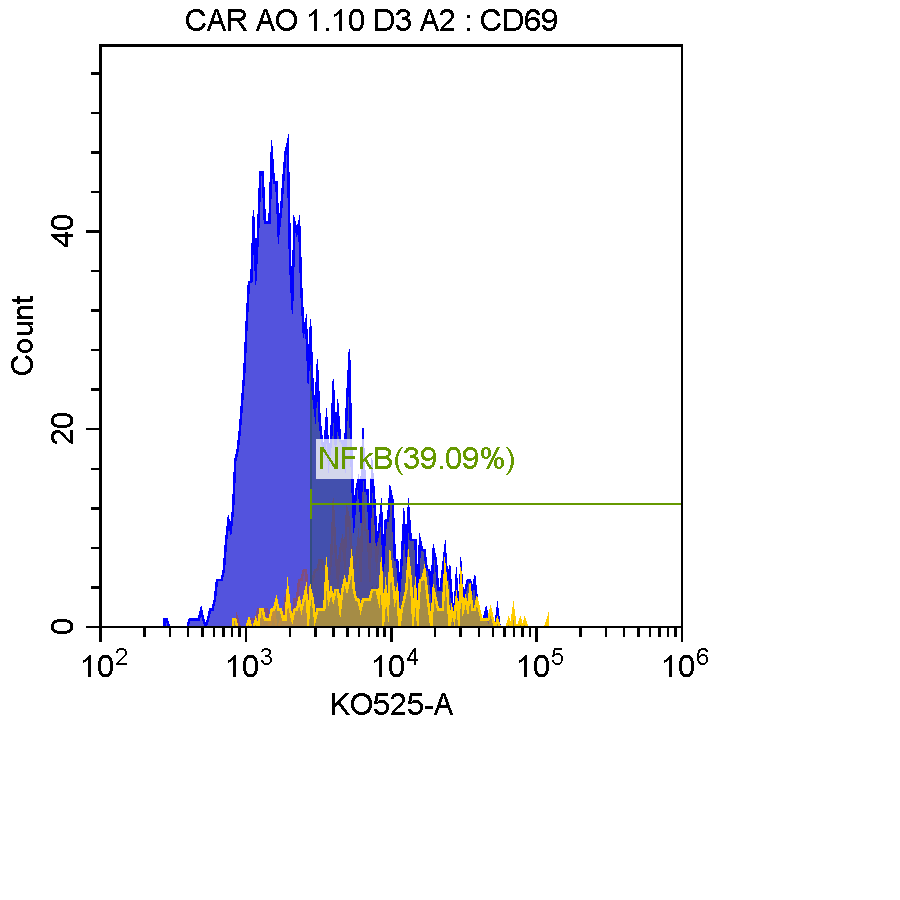

Supplement: Supplementary file 1 [file ijms-24-07641-s001.zip › Cocultures/CAR AO 1.10 D3 A2_Plot4.bmp]

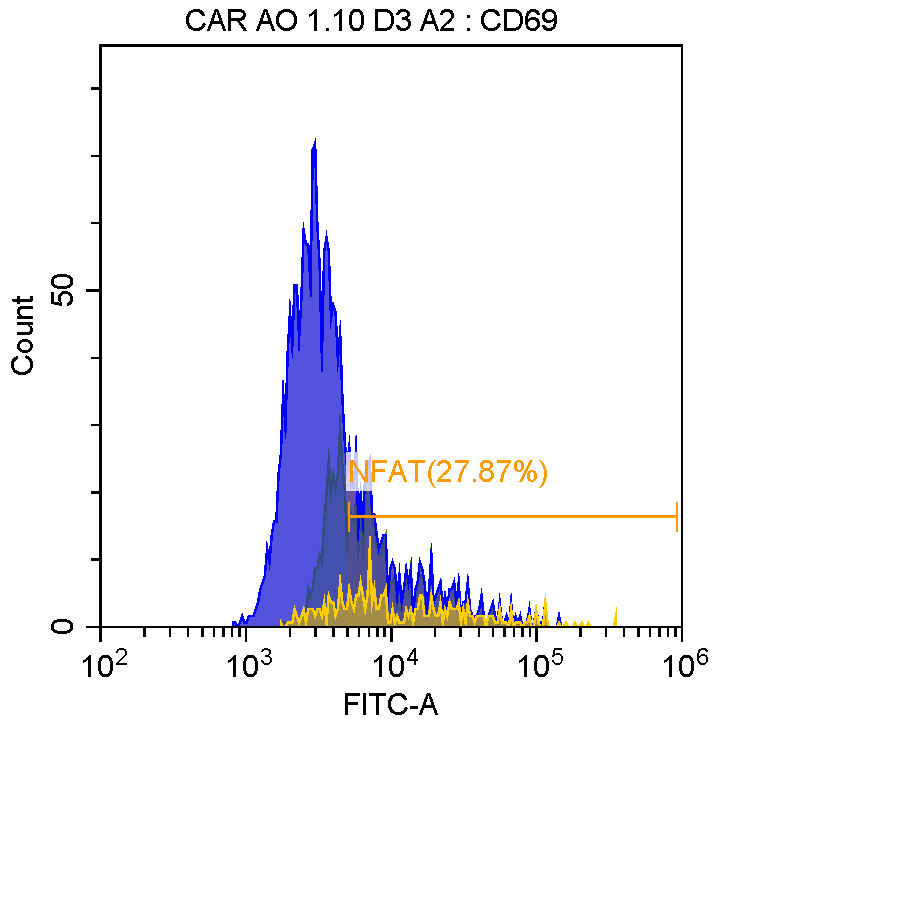

Supplement: Supplementary file 1 [file ijms-24-07641-s001.zip › Cocultures/CAR AO 1.10 D3 A2_Plot5.bmp]

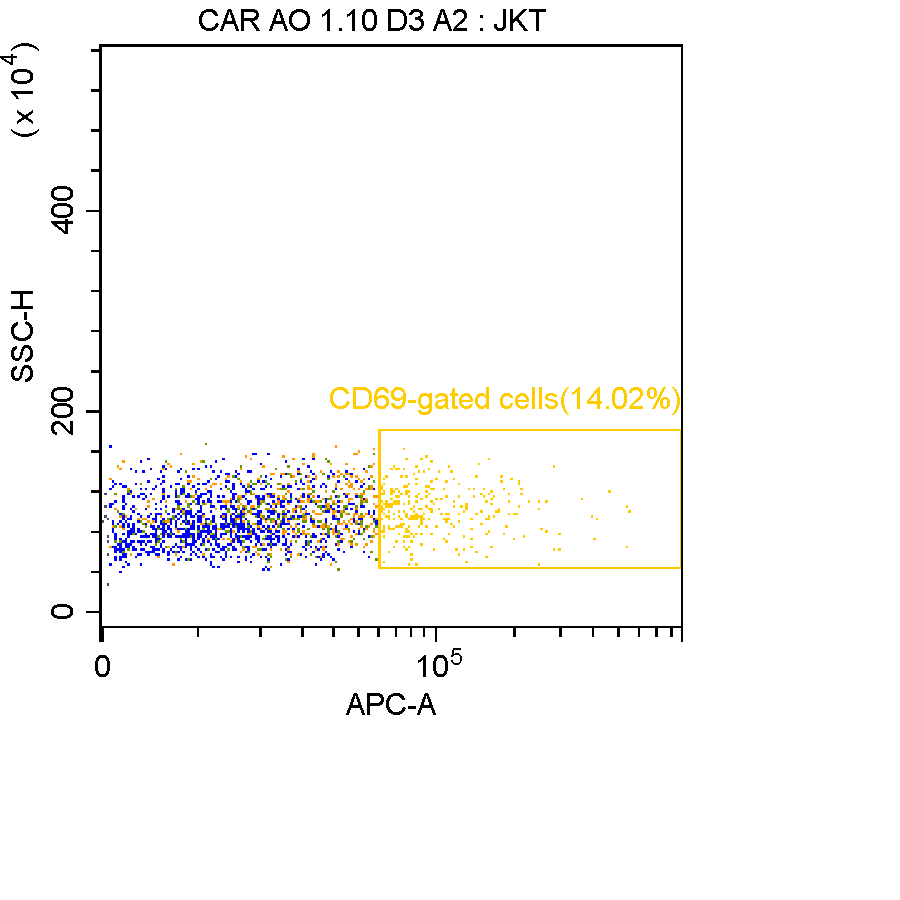

Supplement: Supplementary file 1 [file ijms-24-07641-s001.zip › Cocultures/CAR AO 1.10 D3 A2_Plot6.bmp]

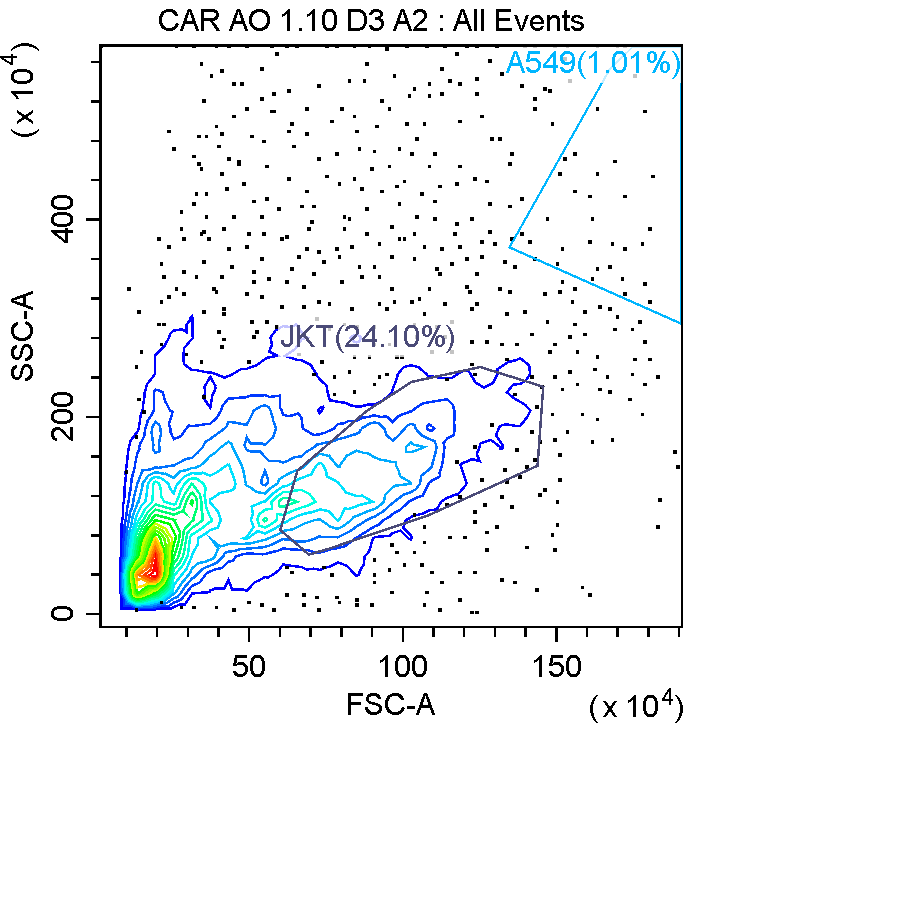

Supplement: Supplementary file 1 [file ijms-24-07641-s001.zip › Cocultures/CAR AO 1.10 D3 A2_Plot7.bmp]

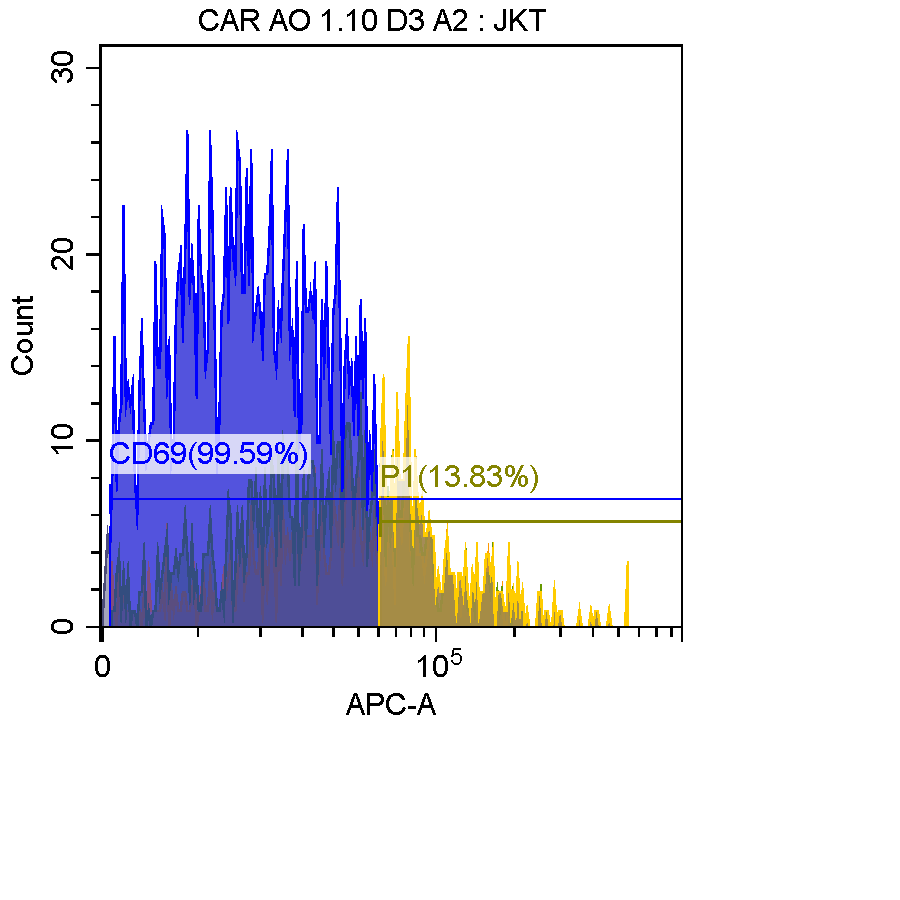

Supplement: Supplementary file 1 [file ijms-24-07641-s001.zip › Cocultures/CAR AO 1.10 D3 A2_Plot8.bmp]

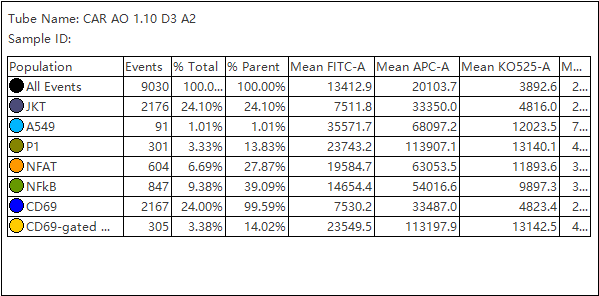

Supplement: Supplementary file 1 [file ijms-24-07641-s001.zip › Cocultures/CAR AO 1.10 D3 A2_Statistics1.bmp]

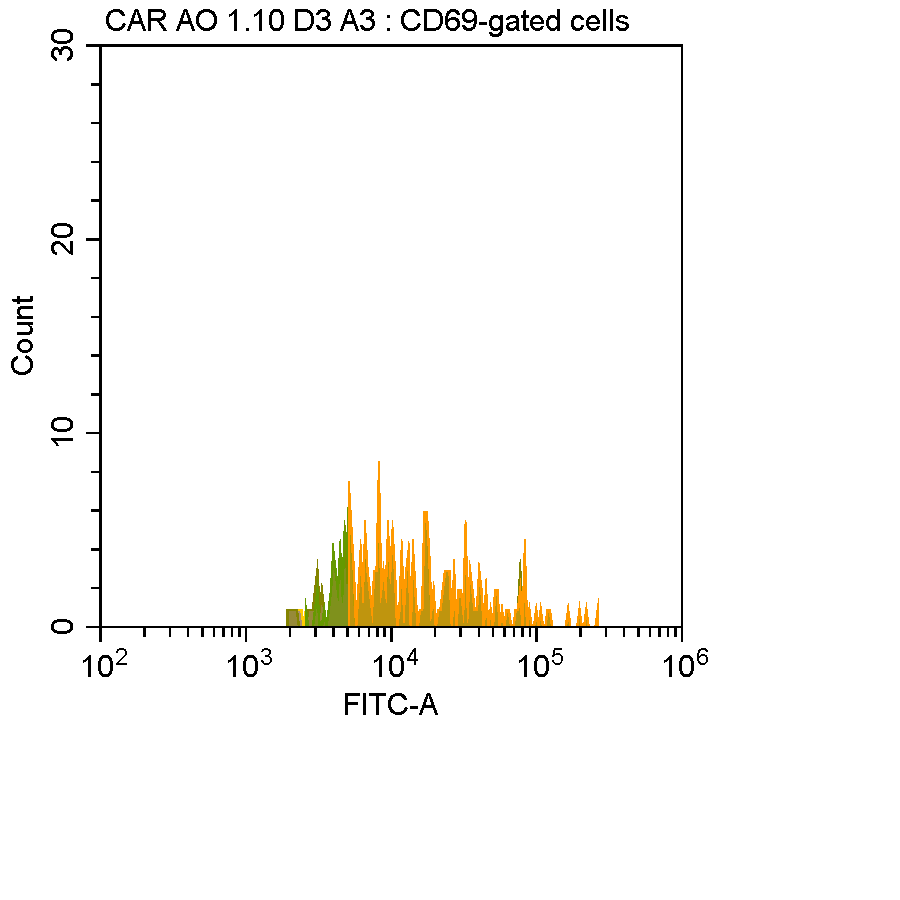

Supplement: Supplementary file 1 [file ijms-24-07641-s001.zip › Cocultures/CAR AO 1.10 D3 A3_Plot1.bmp]

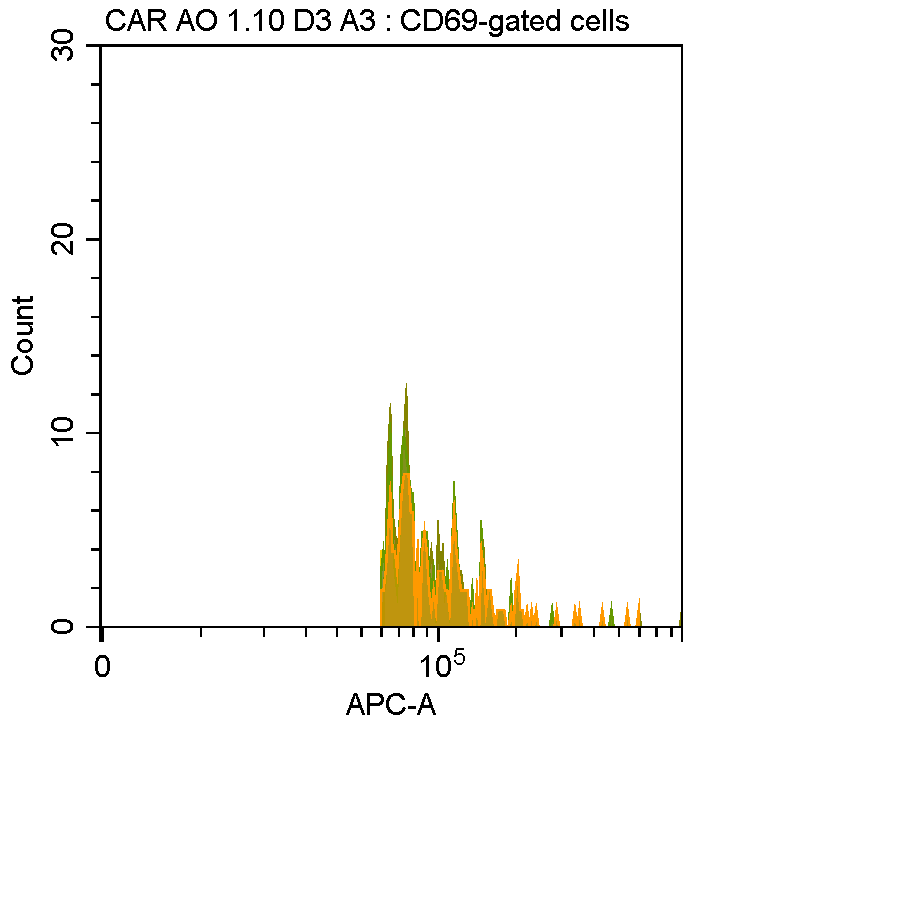

Supplement: Supplementary file 1 [file ijms-24-07641-s001.zip › Cocultures/CAR AO 1.10 D3 A3_Plot2.bmp]

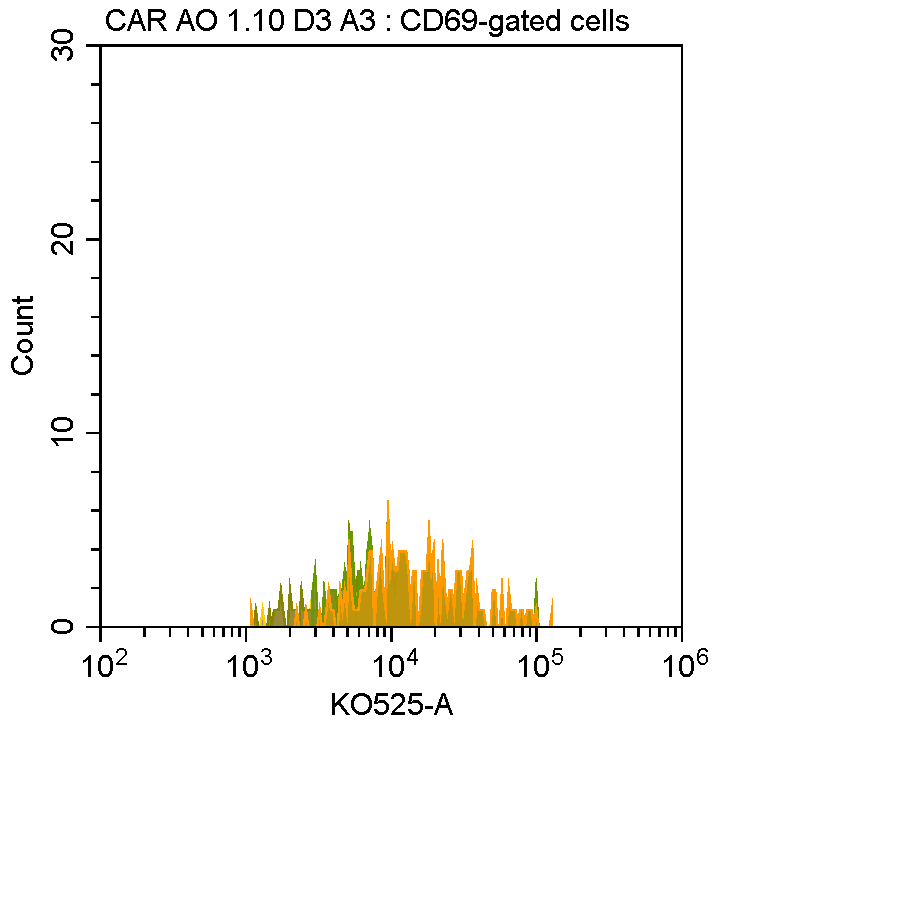

Supplement: Supplementary file 1 [file ijms-24-07641-s001.zip › Cocultures/CAR AO 1.10 D3 A3_Plot3.bmp]

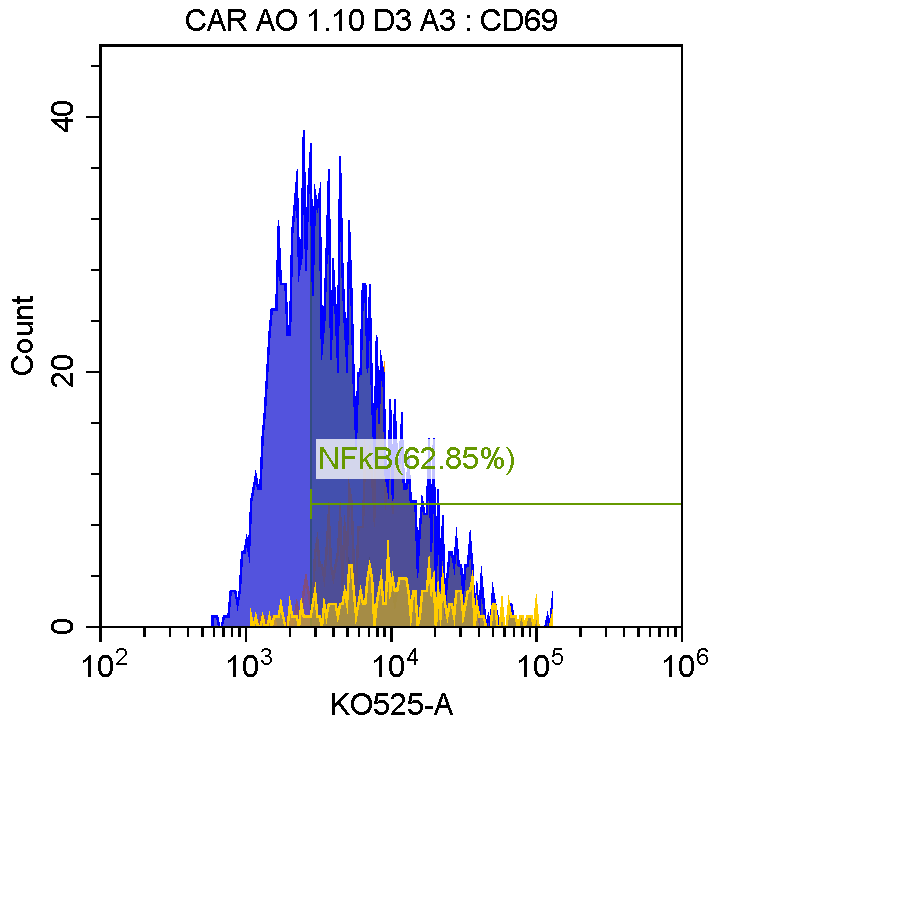

Supplement: Supplementary file 1 [file ijms-24-07641-s001.zip › Cocultures/CAR AO 1.10 D3 A3_Plot4.bmp]

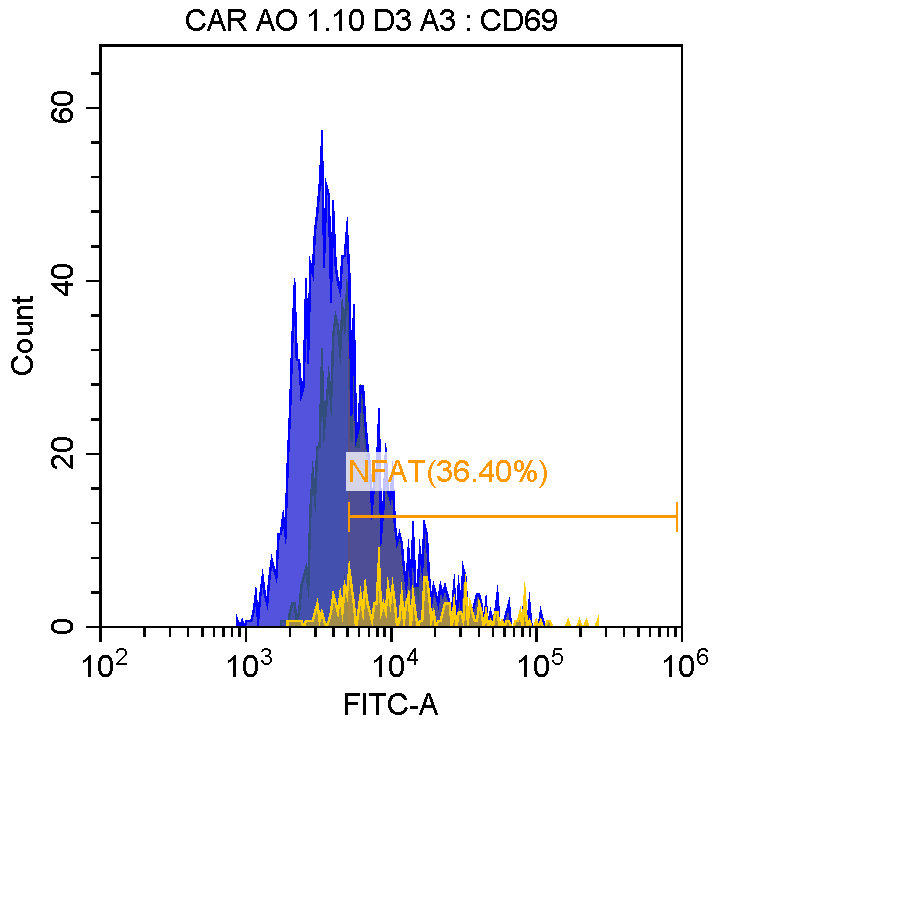

Supplement: Supplementary file 1 [file ijms-24-07641-s001.zip › Cocultures/CAR AO 1.10 D3 A3_Plot5.bmp]

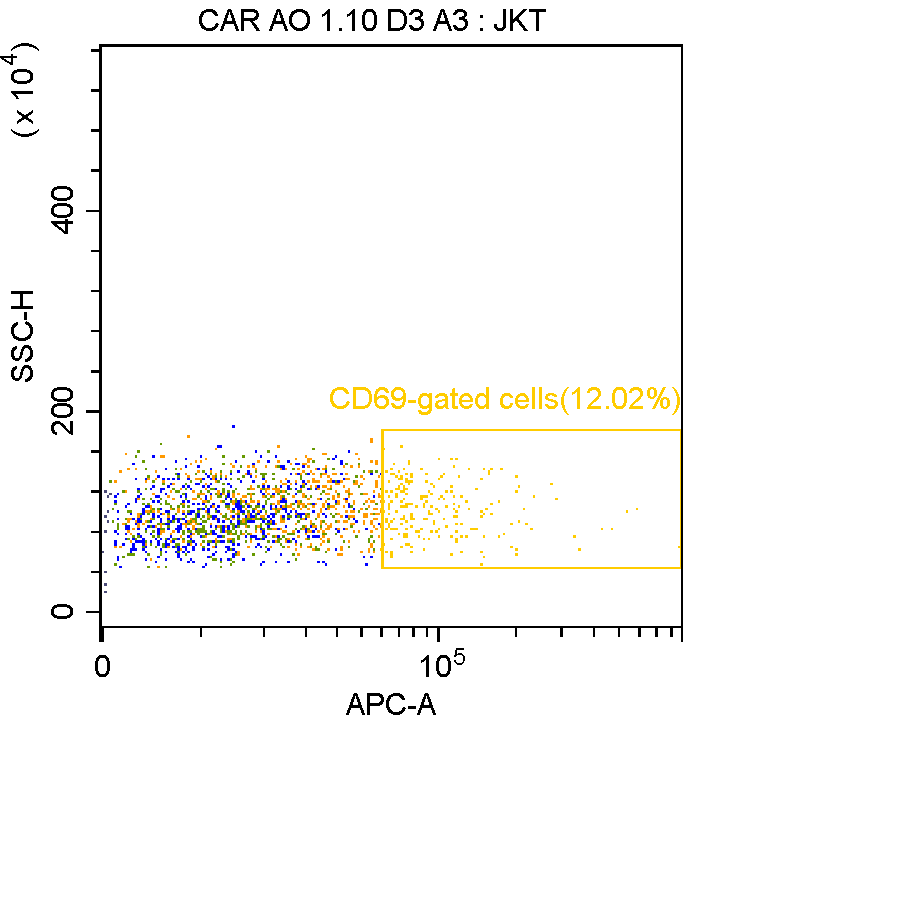

Supplement: Supplementary file 1 [file ijms-24-07641-s001.zip › Cocultures/CAR AO 1.10 D3 A3_Plot6.bmp]

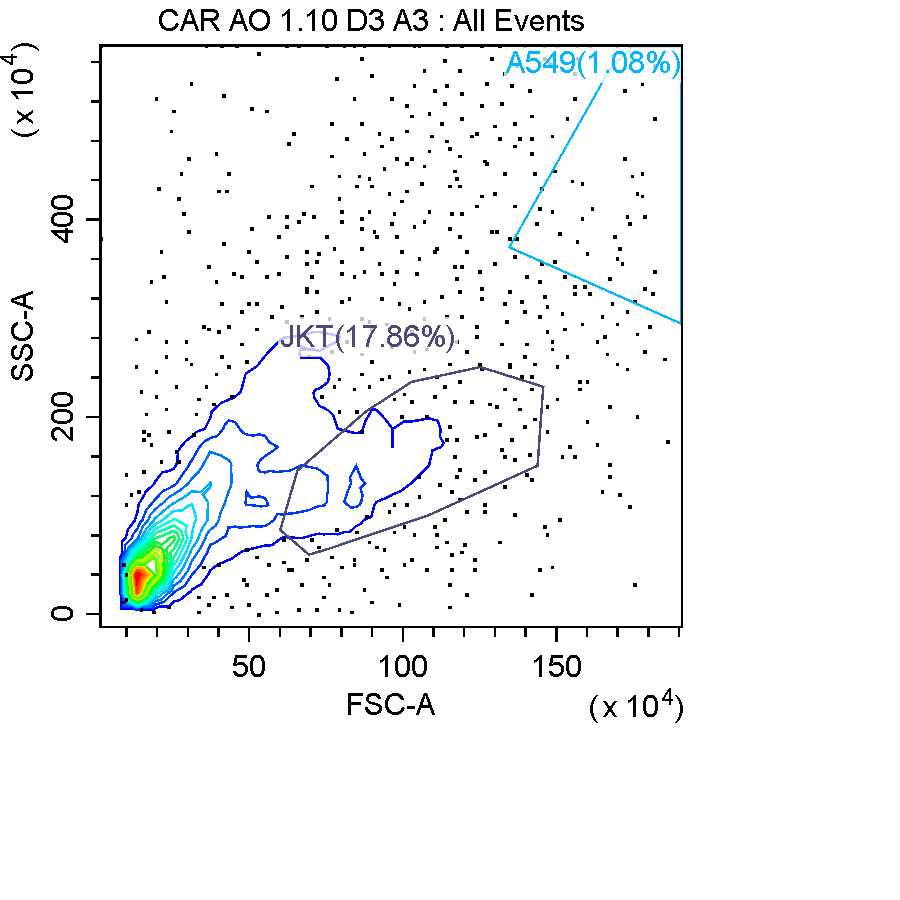

Supplement: Supplementary file 1 [file ijms-24-07641-s001.zip › Cocultures/CAR AO 1.10 D3 A3_Plot7.bmp]

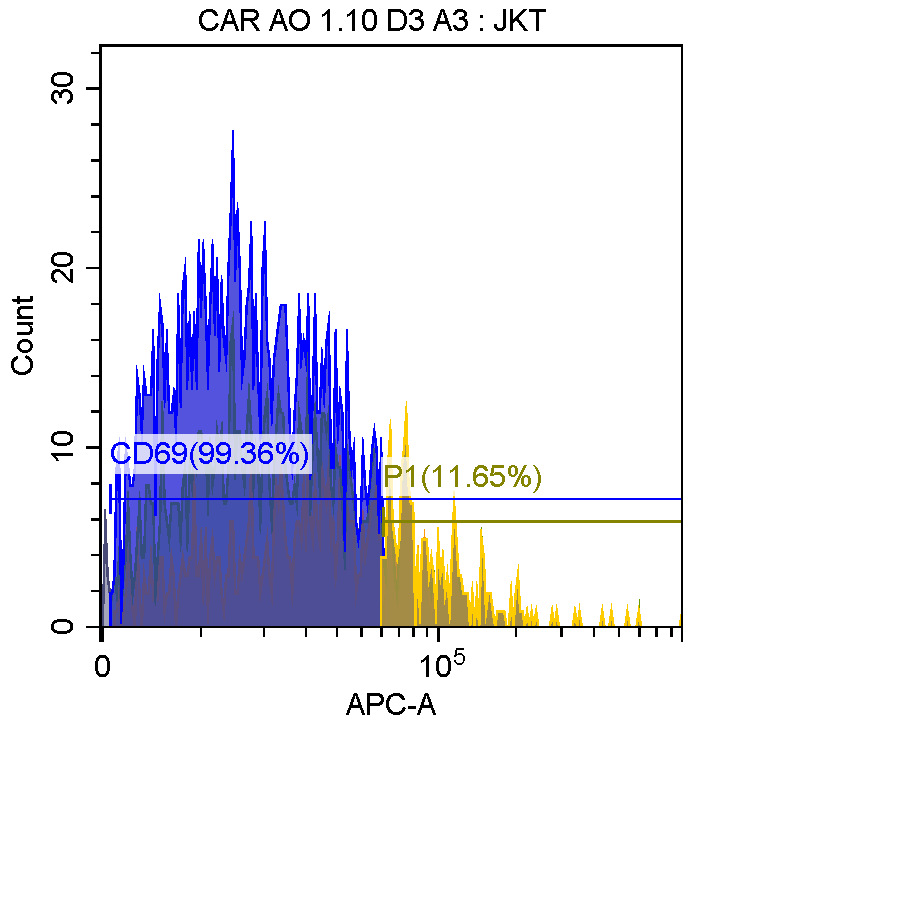

Supplement: Supplementary file 1 [file ijms-24-07641-s001.zip › Cocultures/CAR AO 1.10 D3 A3_Plot8.bmp]

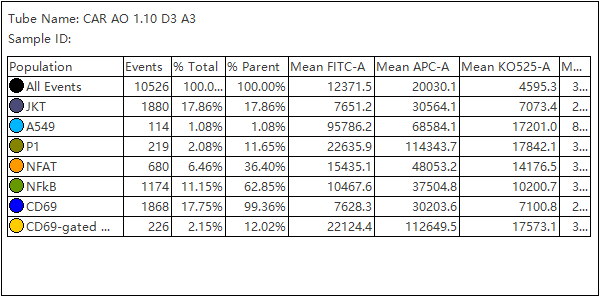

Supplement: Supplementary file 1 [file ijms-24-07641-s001.zip › Cocultures/CAR AO 1.10 D3 A3_Statistics1.bmp]

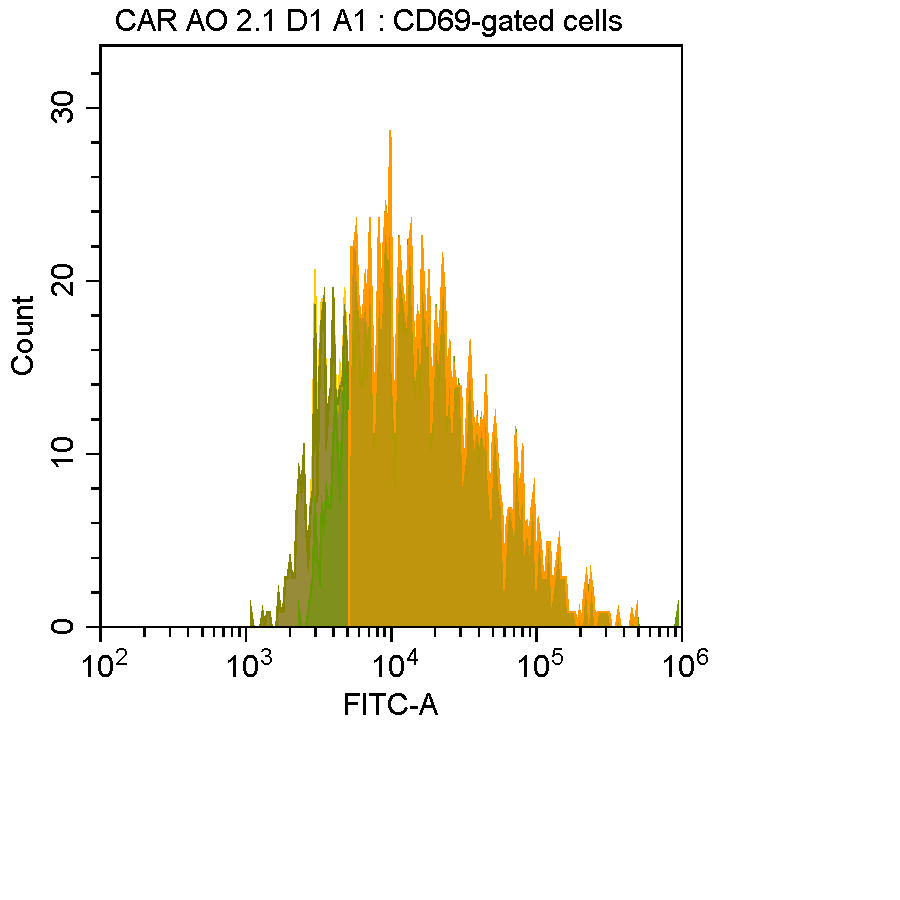

Supplement: Supplementary file 1 [file ijms-24-07641-s001.zip › Cocultures/CAR AO 2.1 D1 A1_Plot1.bmp]

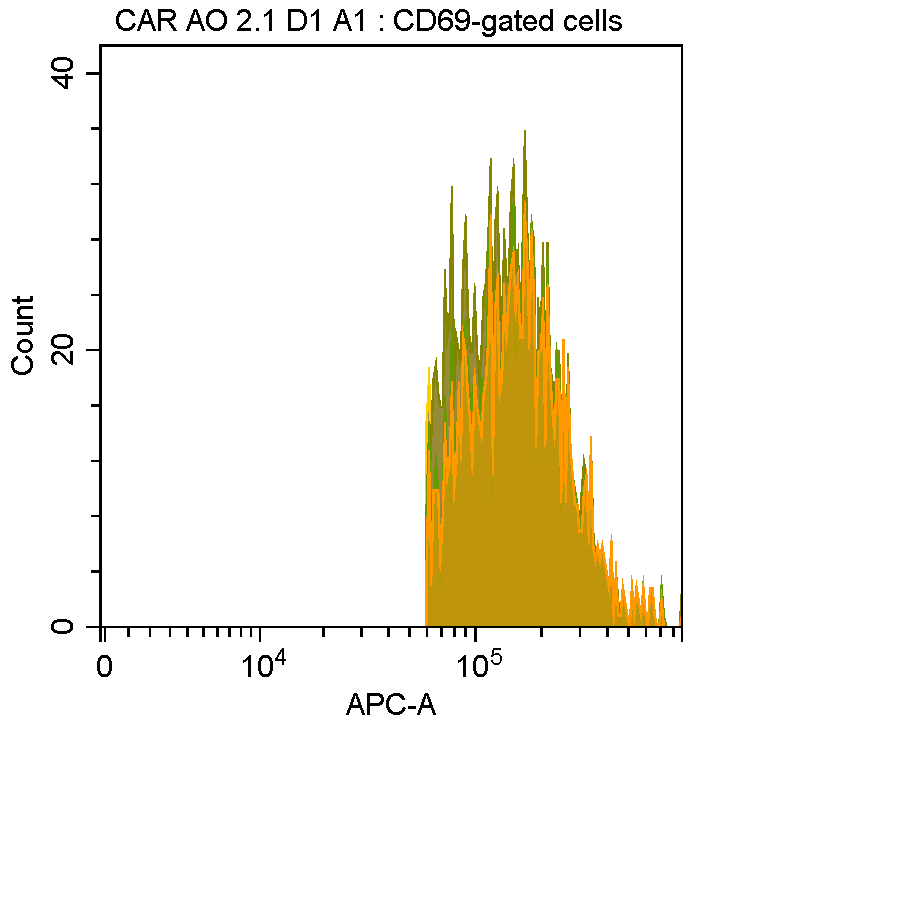

Supplement: Supplementary file 1 [file ijms-24-07641-s001.zip › Cocultures/CAR AO 2.1 D1 A1_Plot2.bmp]

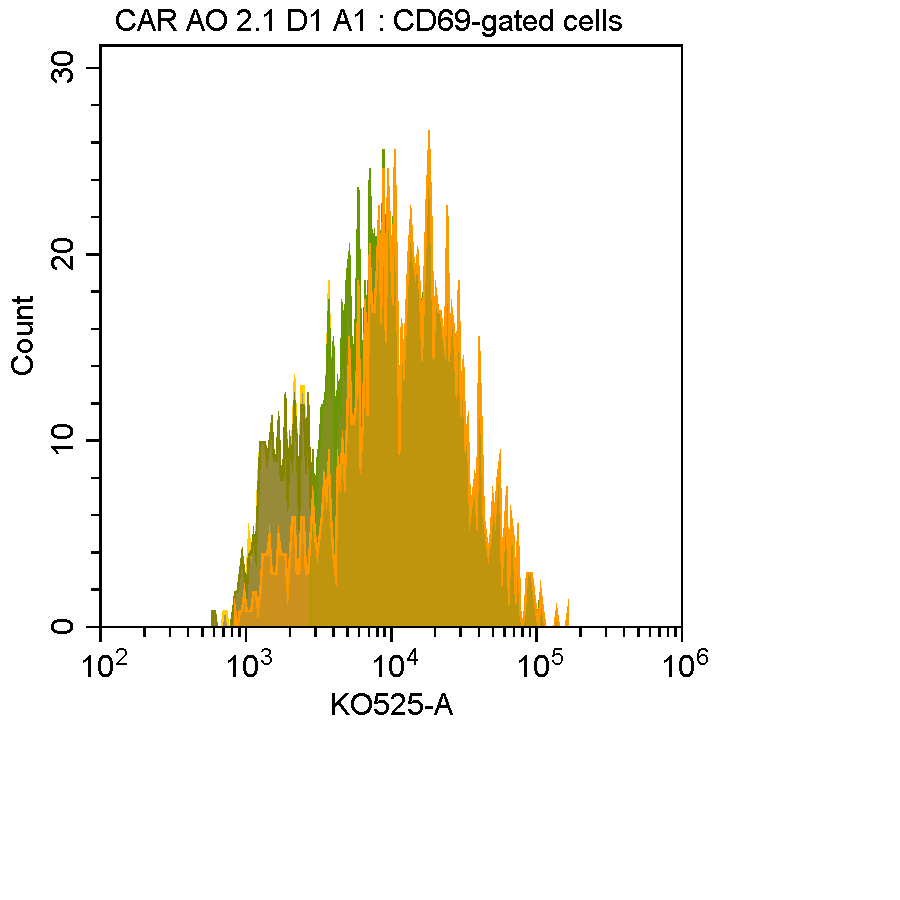

Supplement: Supplementary file 1 [file ijms-24-07641-s001.zip › Cocultures/CAR AO 2.1 D1 A1_Plot3.bmp]

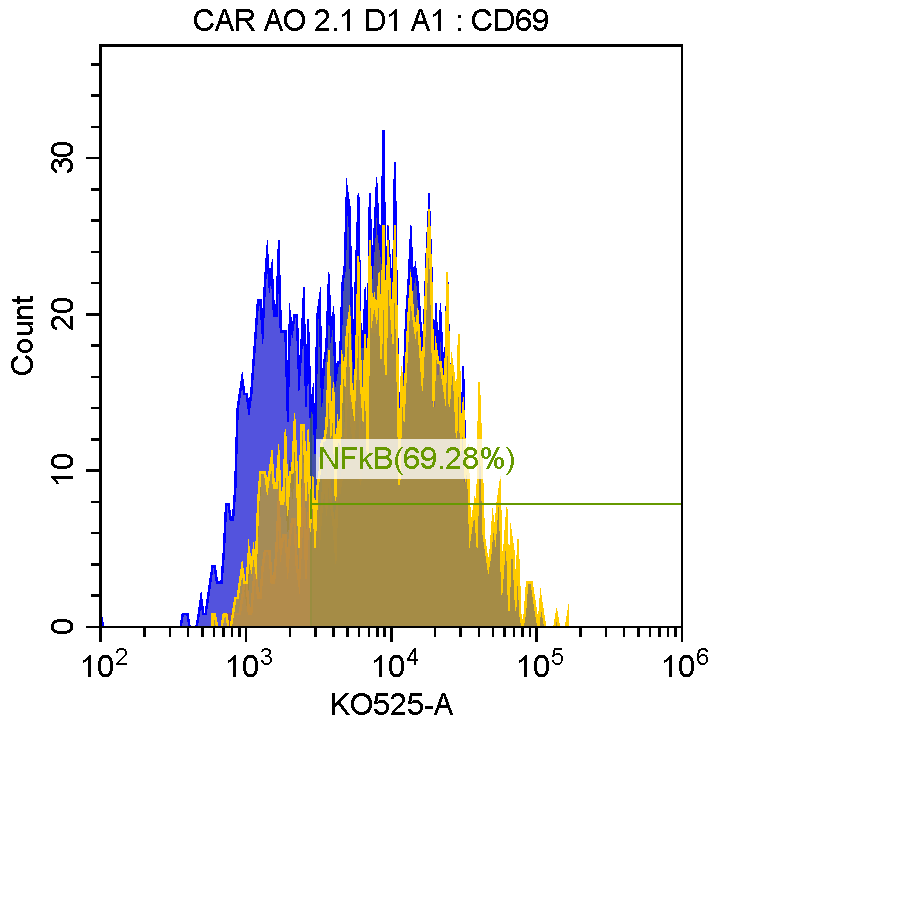

Supplement: Supplementary file 1 [file ijms-24-07641-s001.zip › Cocultures/CAR AO 2.1 D1 A1_Plot4.bmp]

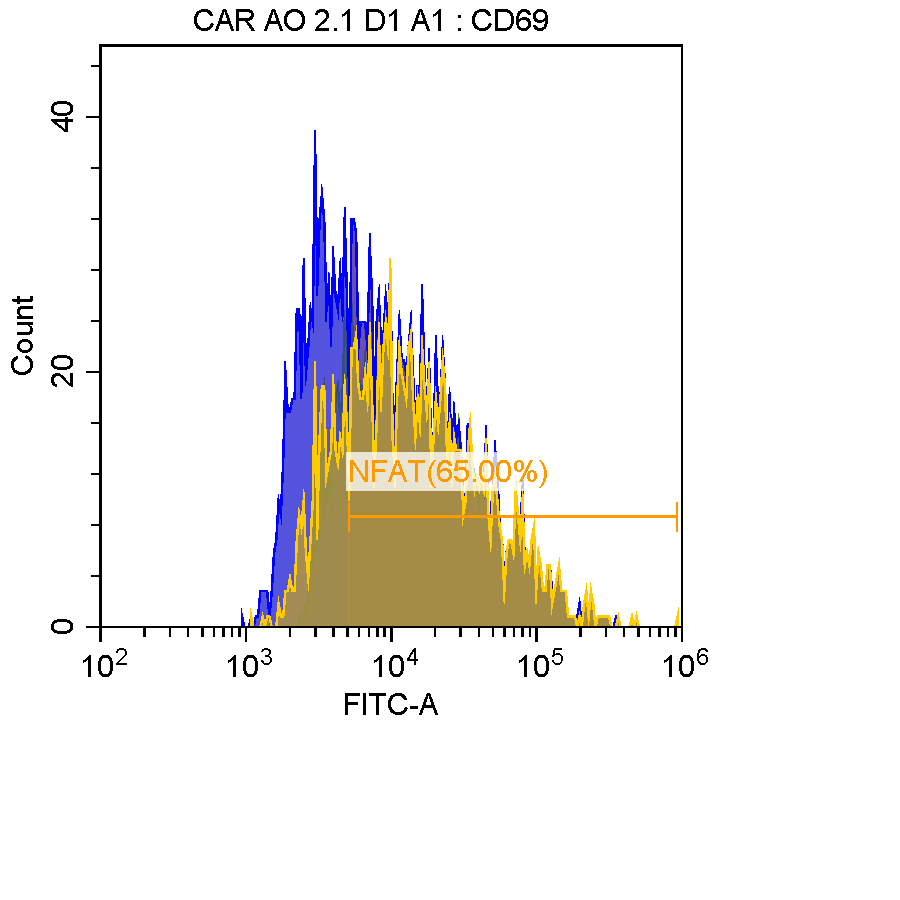

Supplement: Supplementary file 1 [file ijms-24-07641-s001.zip › Cocultures/CAR AO 2.1 D1 A1_Plot5.bmp]

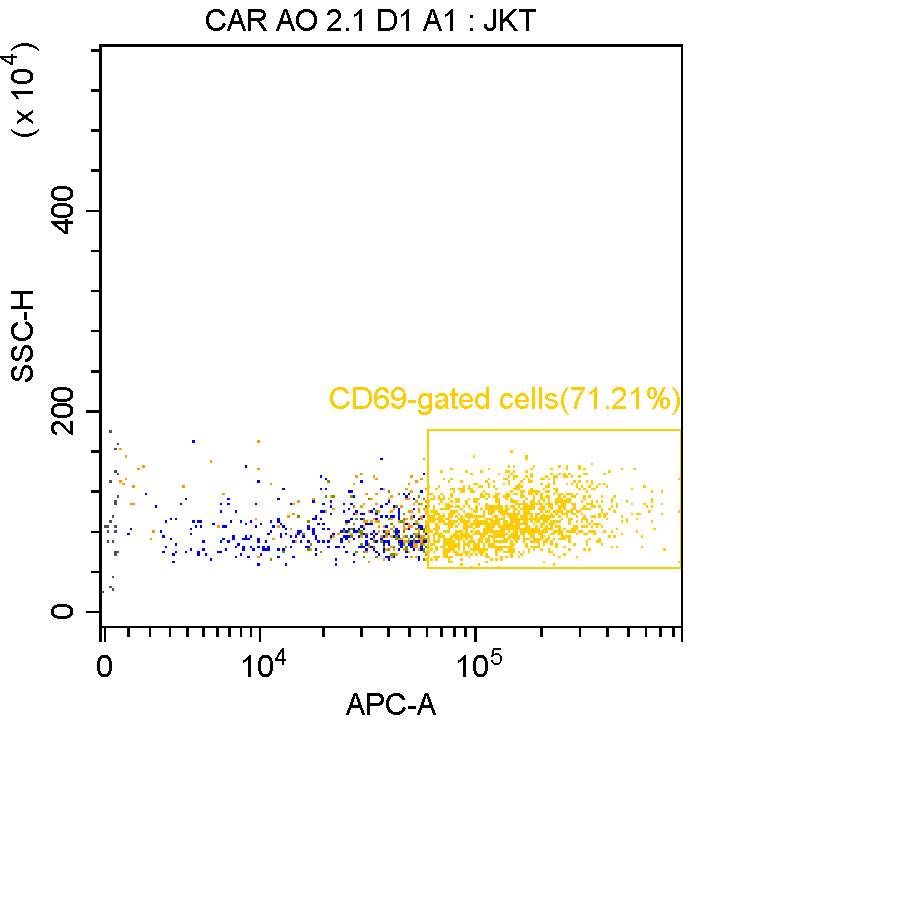

Supplement: Supplementary file 1 [file ijms-24-07641-s001.zip › Cocultures/CAR AO 2.1 D1 A1_Plot6.bmp]

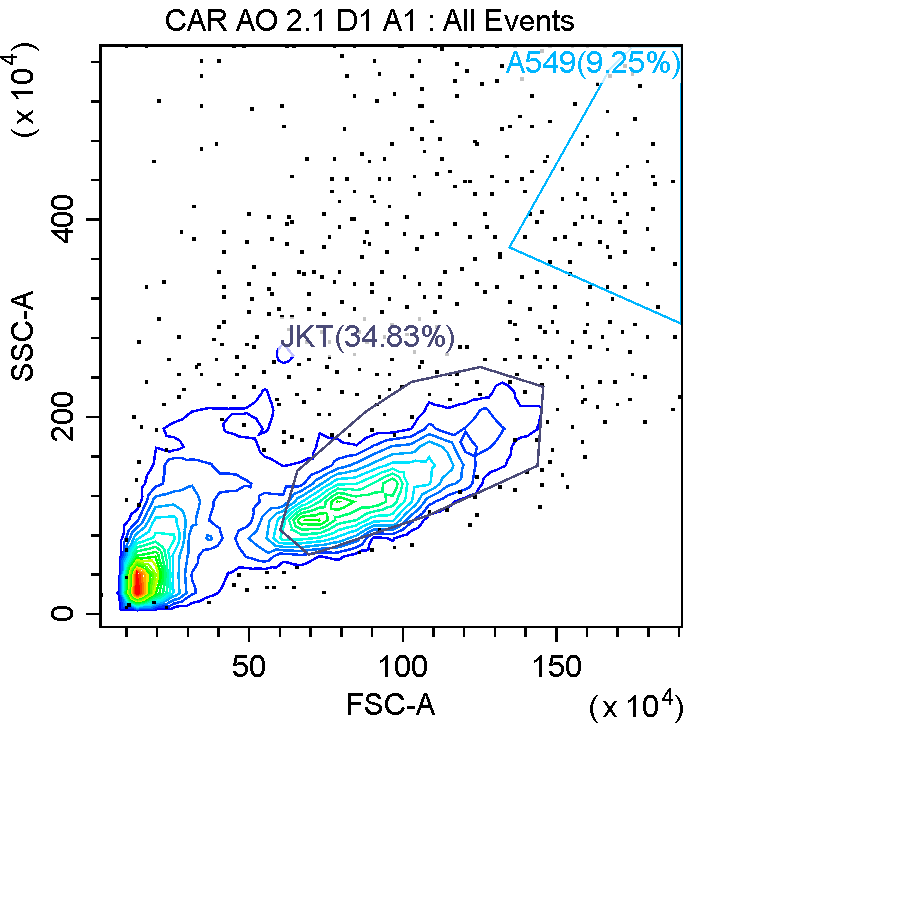

Supplement: Supplementary file 1 [file ijms-24-07641-s001.zip › Cocultures/CAR AO 2.1 D1 A1_Plot7.bmp]

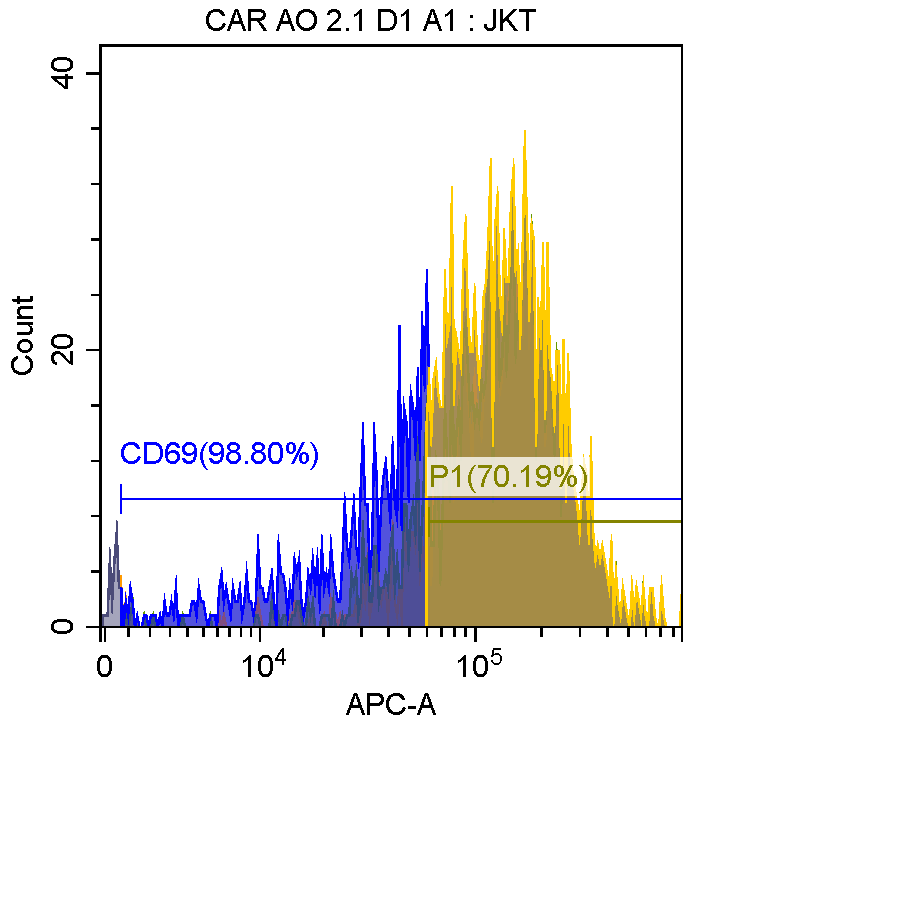

Supplement: Supplementary file 1 [file ijms-24-07641-s001.zip › Cocultures/CAR AO 2.1 D1 A1_Plot8.bmp]

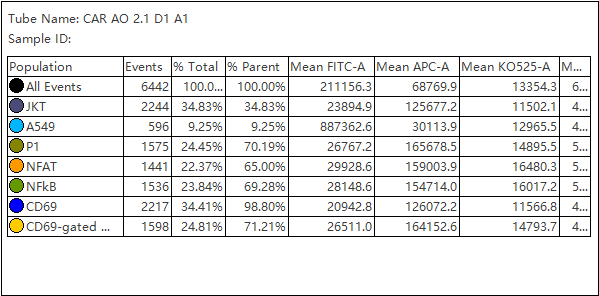

Supplement: Supplementary file 1 [file ijms-24-07641-s001.zip › Cocultures/CAR AO 2.1 D1 A1_Statistics1.bmp]

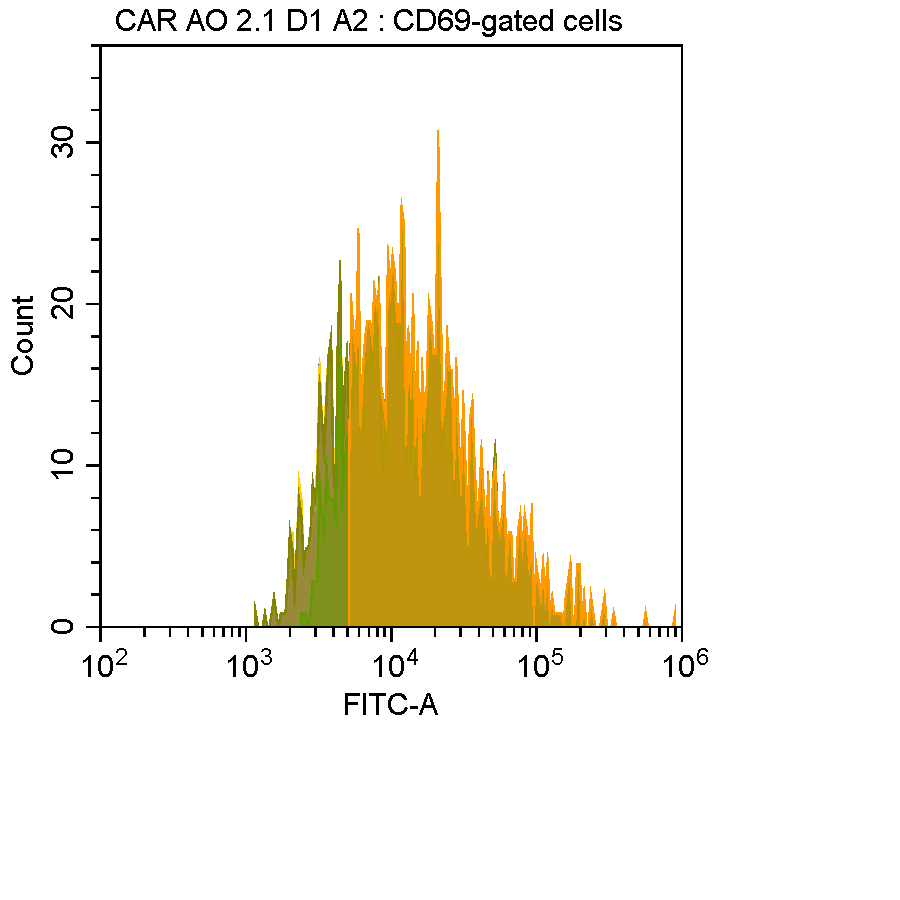

Supplement: Supplementary file 1 [file ijms-24-07641-s001.zip › Cocultures/CAR AO 2.1 D1 A2_Plot1.bmp]

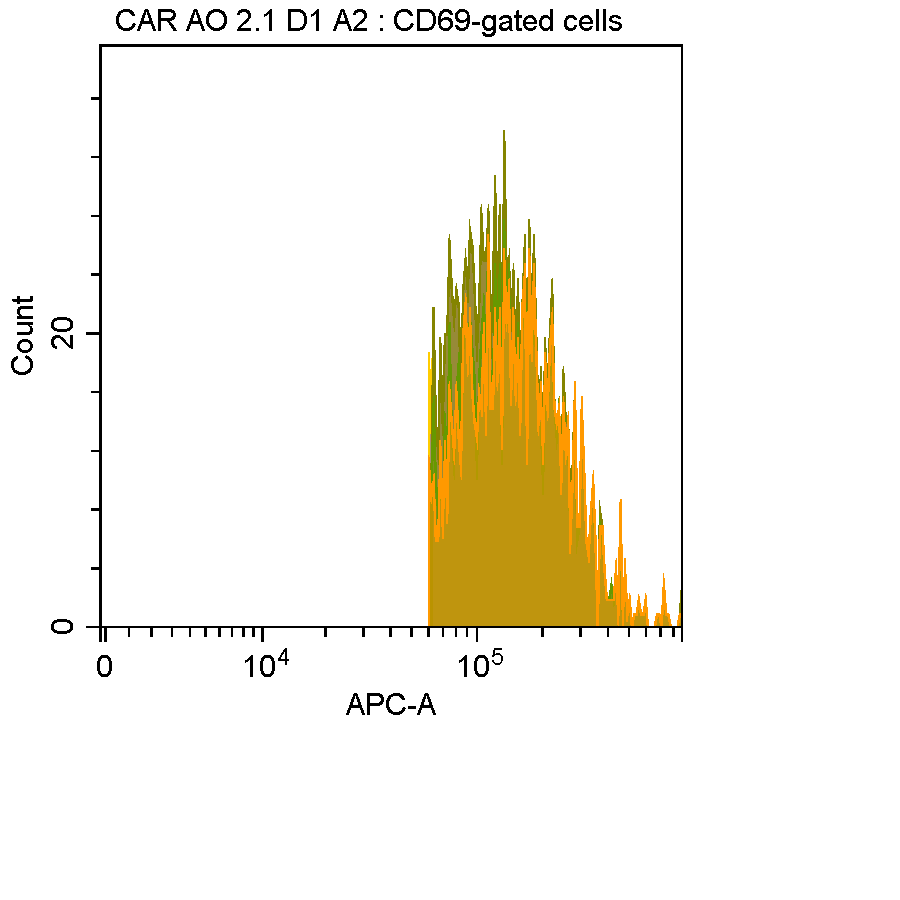

Supplement: Supplementary file 1 [file ijms-24-07641-s001.zip › Cocultures/CAR AO 2.1 D1 A2_Plot2.bmp]

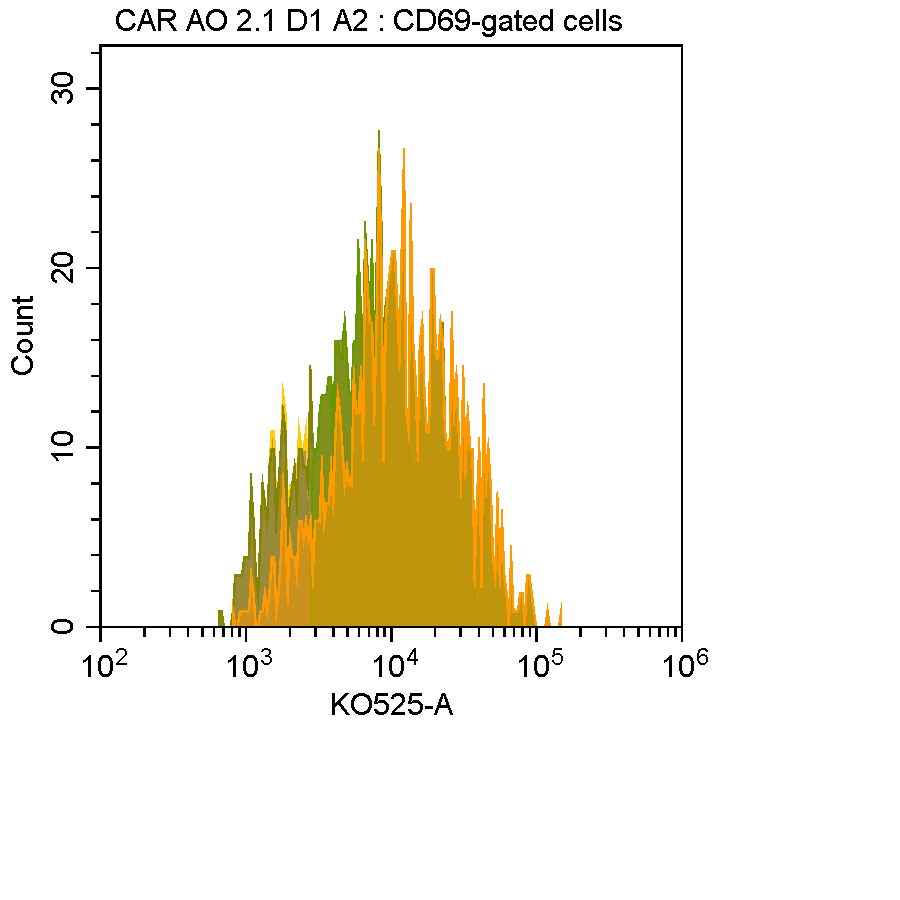

Supplement: Supplementary file 1 [file ijms-24-07641-s001.zip › Cocultures/CAR AO 2.1 D1 A2_Plot3.bmp]

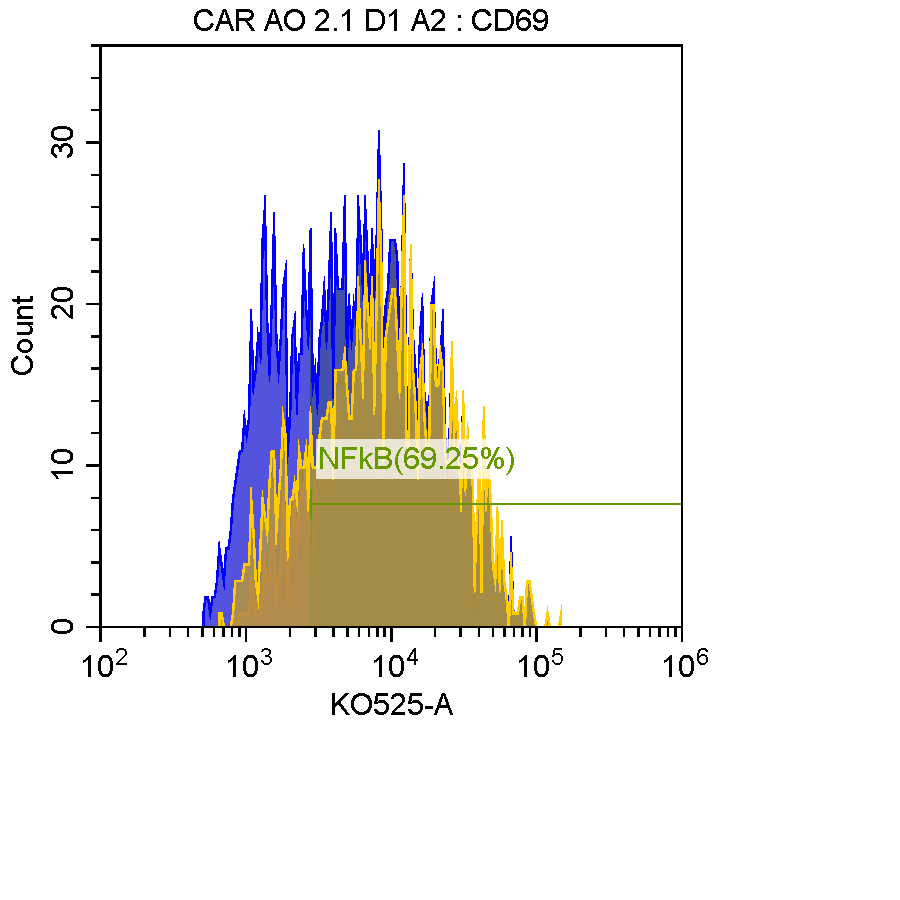

Supplement: Supplementary file 1 [file ijms-24-07641-s001.zip › Cocultures/CAR AO 2.1 D1 A2_Plot4.bmp]

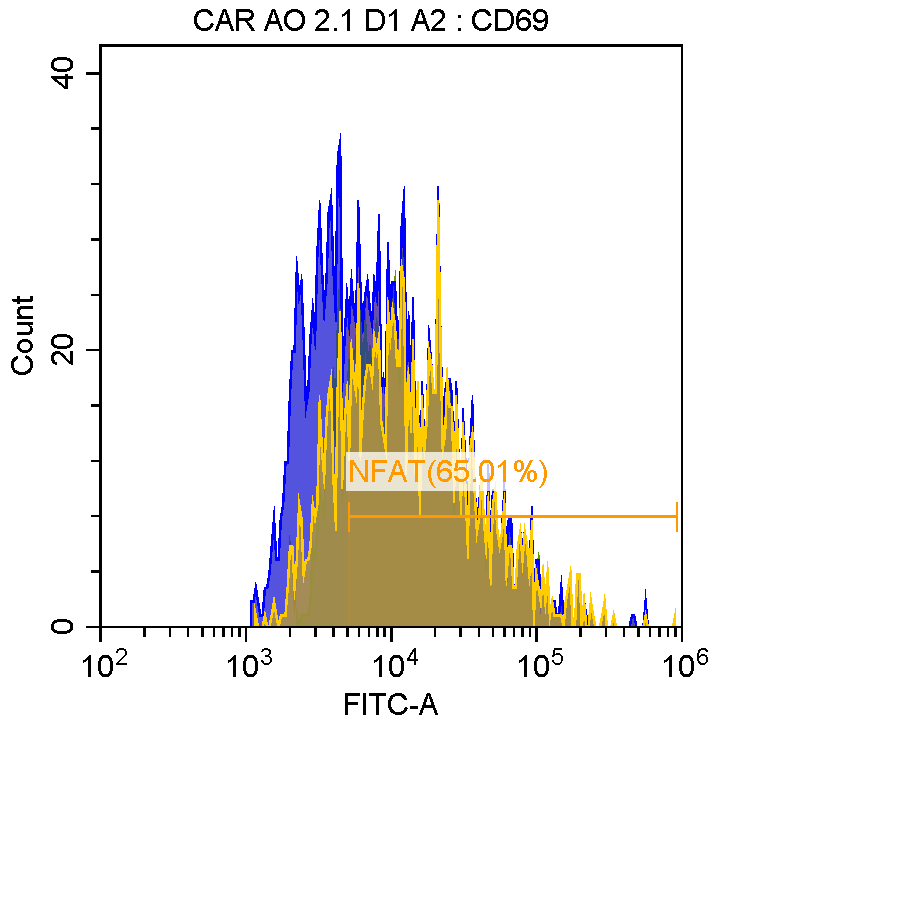

Supplement: Supplementary file 1 [file ijms-24-07641-s001.zip › Cocultures/CAR AO 2.1 D1 A2_Plot5.bmp]

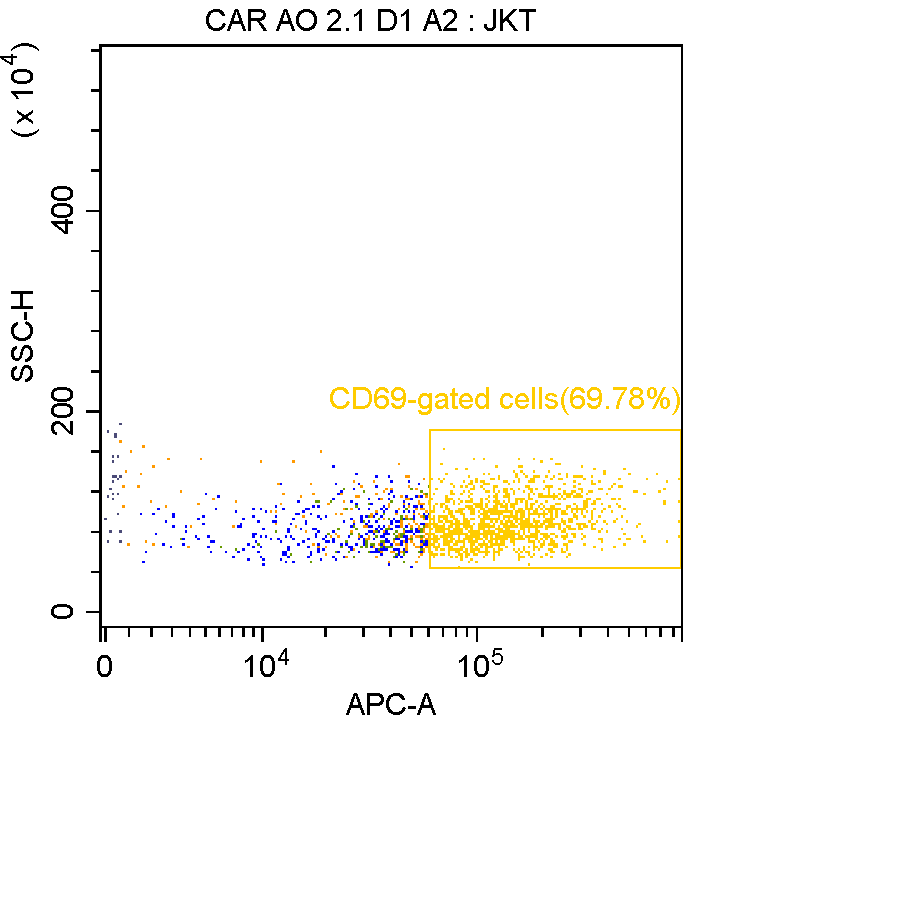

Supplement: Supplementary file 1 [file ijms-24-07641-s001.zip › Cocultures/CAR AO 2.1 D1 A2_Plot6.bmp]

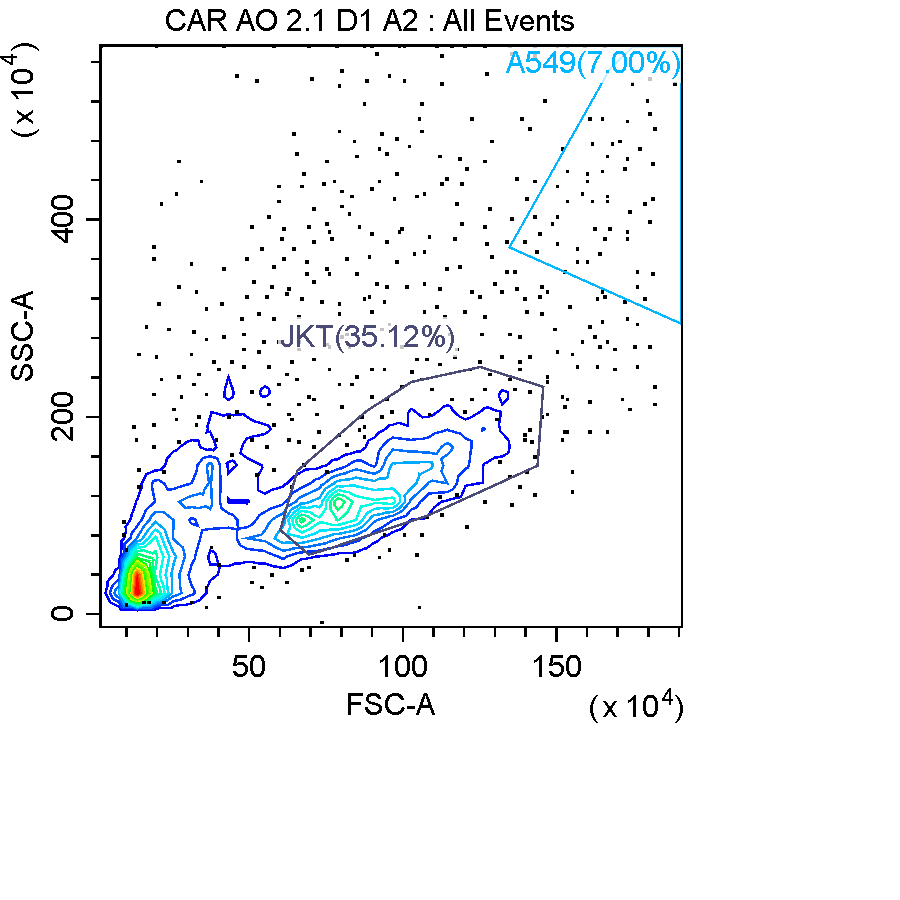

Supplement: Supplementary file 1 [file ijms-24-07641-s001.zip › Cocultures/CAR AO 2.1 D1 A2_Plot7.bmp]

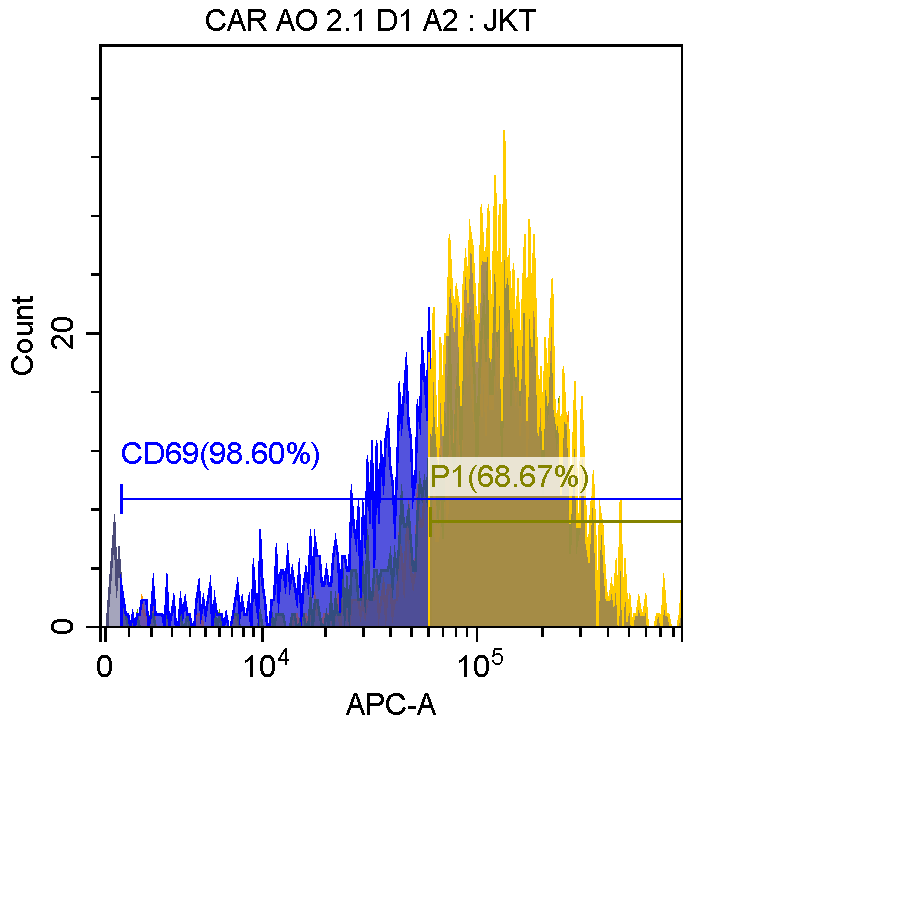

Supplement: Supplementary file 1 [file ijms-24-07641-s001.zip › Cocultures/CAR AO 2.1 D1 A2_Plot8.bmp]

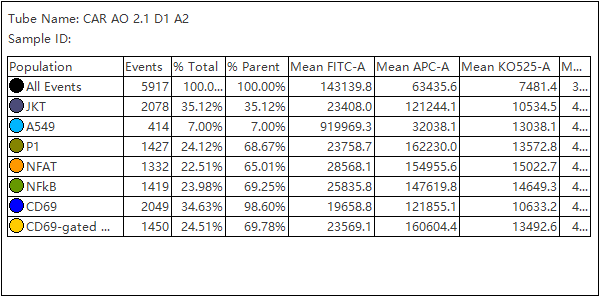

Supplement: Supplementary file 1 [file ijms-24-07641-s001.zip › Cocultures/CAR AO 2.1 D1 A2_Statistics1.bmp]

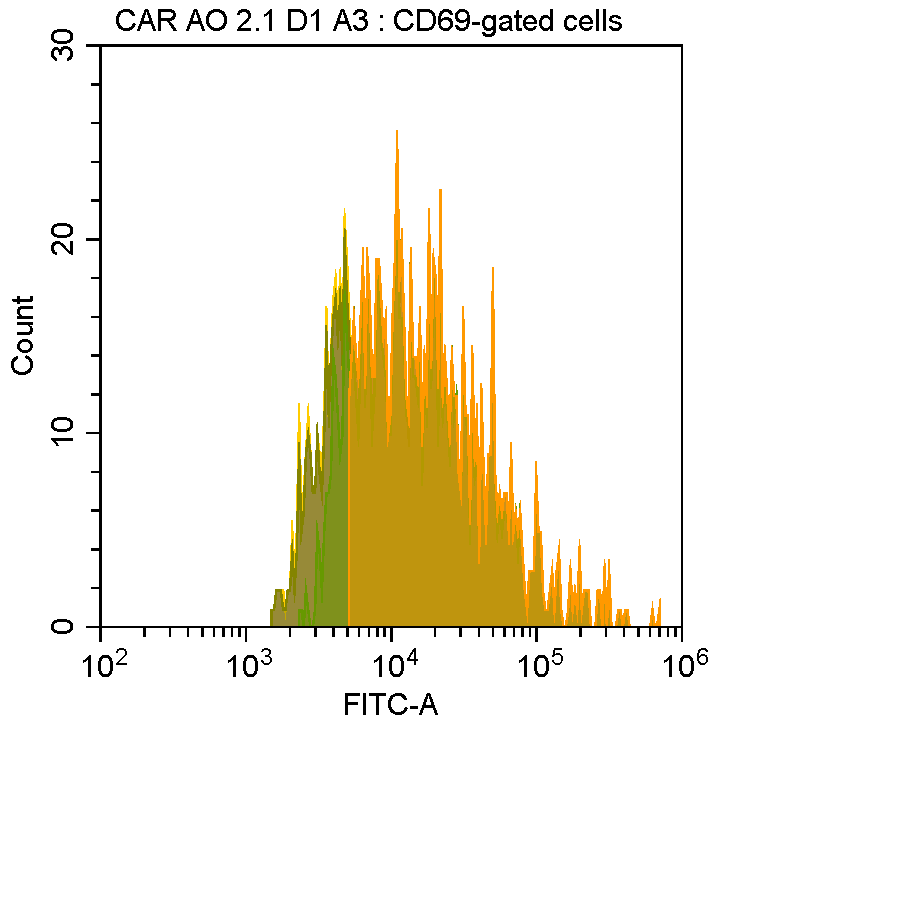

Supplement: Supplementary file 1 [file ijms-24-07641-s001.zip › Cocultures/CAR AO 2.1 D1 A3_Plot1.bmp]

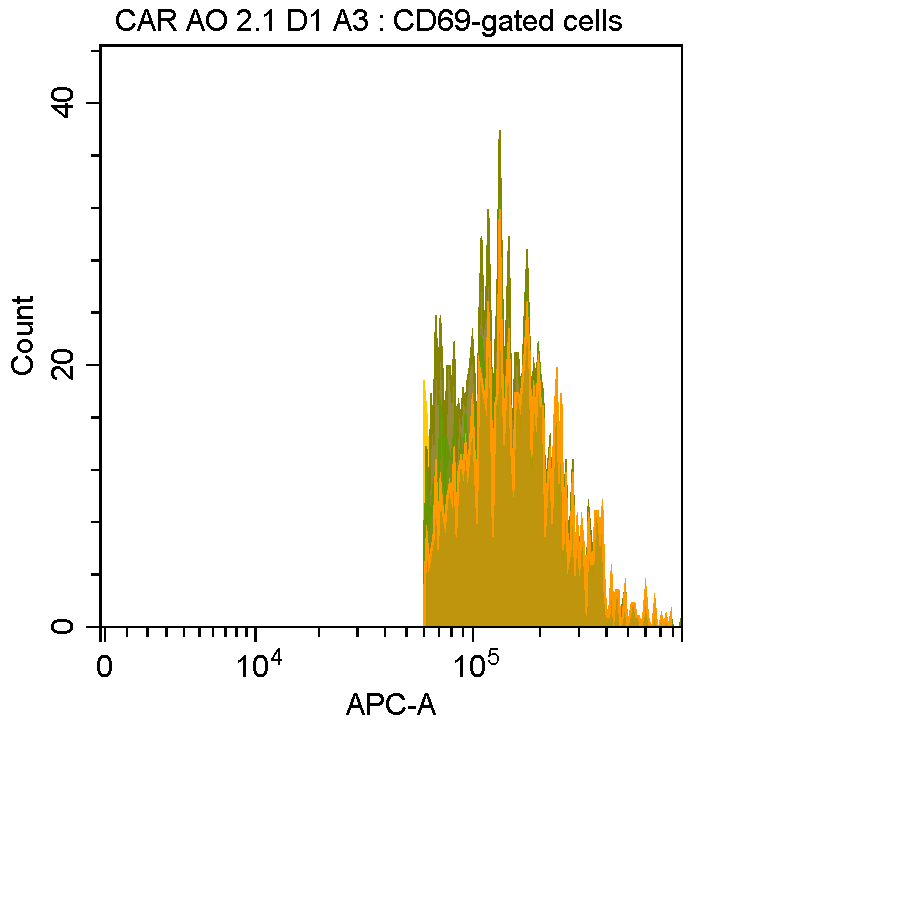

Supplement: Supplementary file 1 [file ijms-24-07641-s001.zip › Cocultures/CAR AO 2.1 D1 A3_Plot2.bmp]

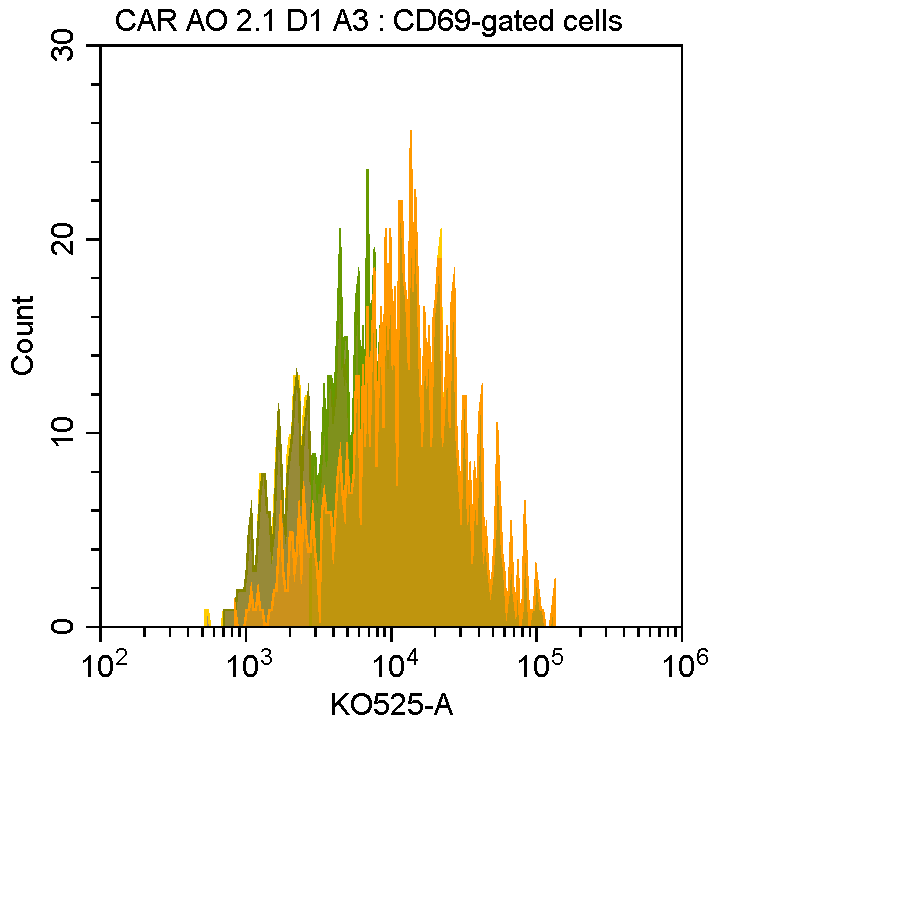

Supplement: Supplementary file 1 [file ijms-24-07641-s001.zip › Cocultures/CAR AO 2.1 D1 A3_Plot3.bmp]

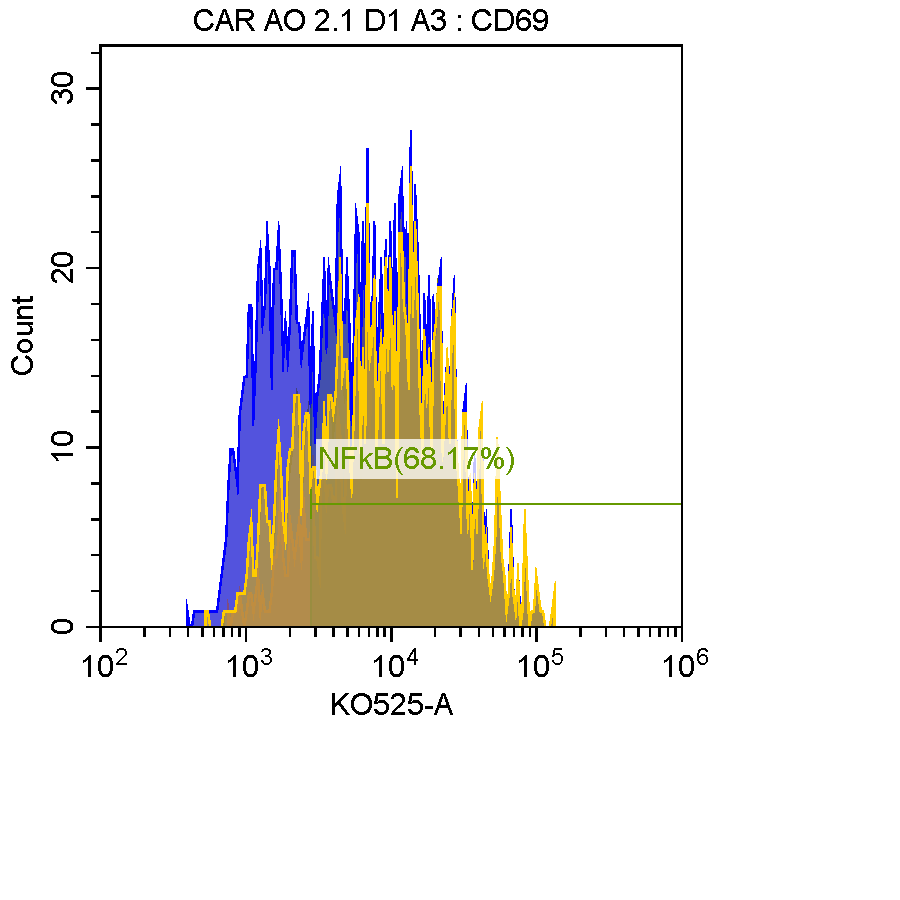

Supplement: Supplementary file 1 [file ijms-24-07641-s001.zip › Cocultures/CAR AO 2.1 D1 A3_Plot4.bmp]

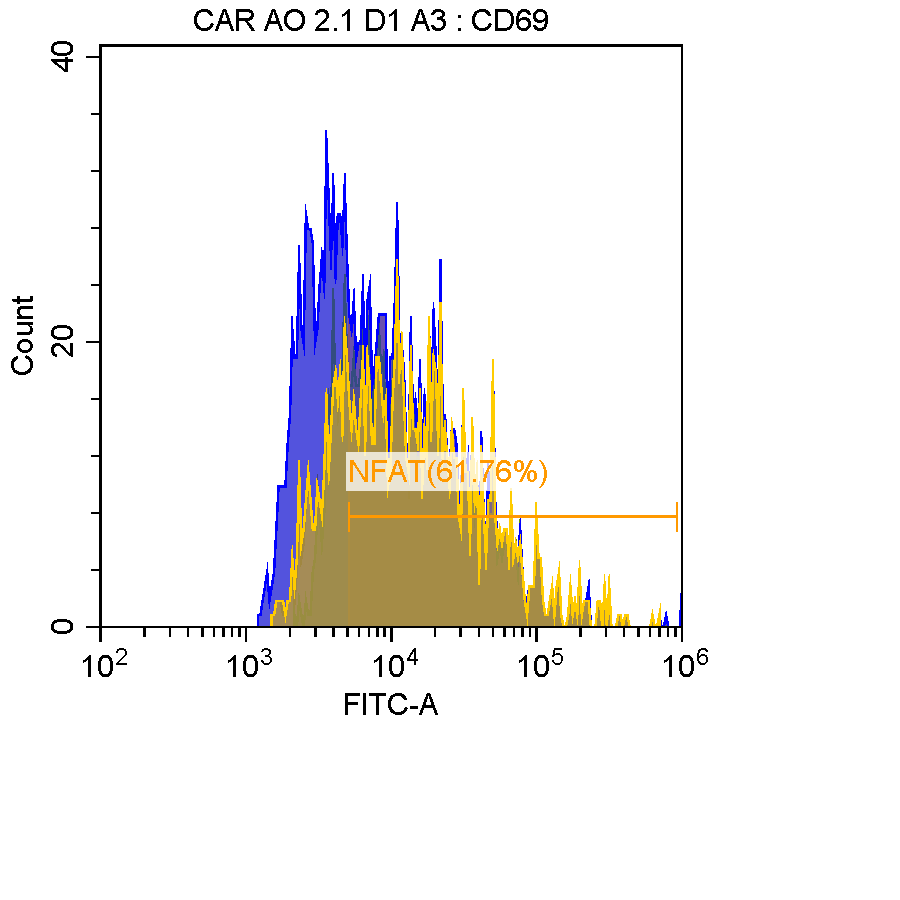

Supplement: Supplementary file 1 [file ijms-24-07641-s001.zip › Cocultures/CAR AO 2.1 D1 A3_Plot5.bmp]

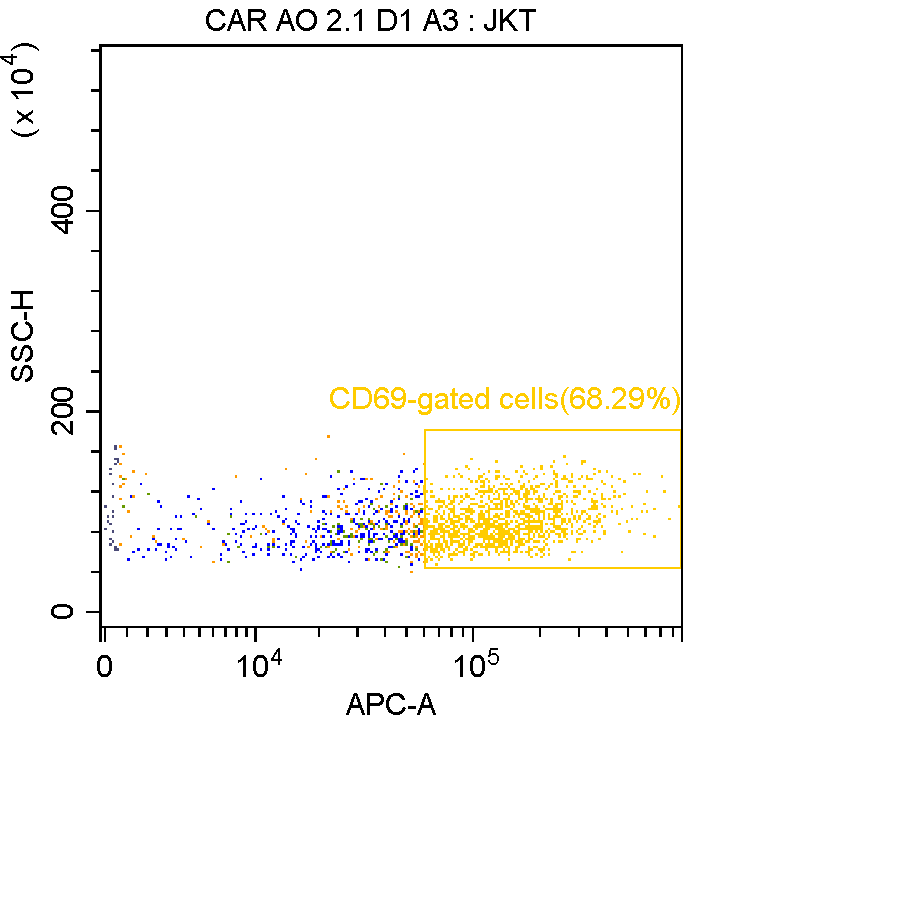

Supplement: Supplementary file 1 [file ijms-24-07641-s001.zip › Cocultures/CAR AO 2.1 D1 A3_Plot6.bmp]

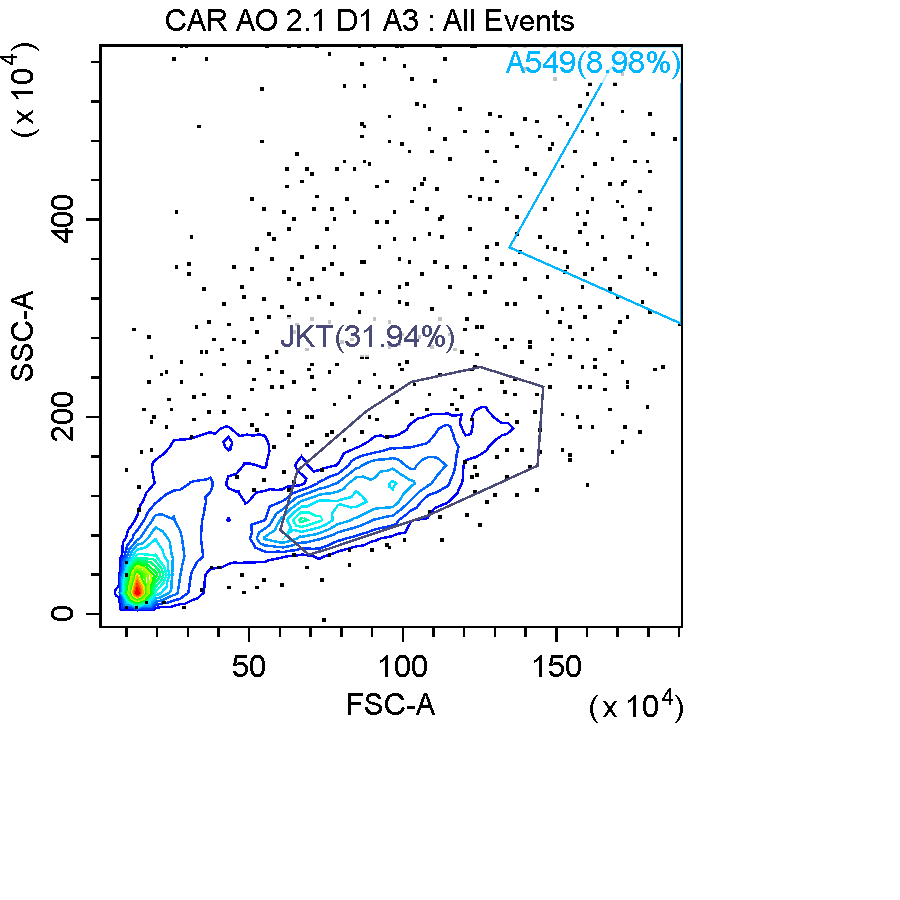

Supplement: Supplementary file 1 [file ijms-24-07641-s001.zip › Cocultures/CAR AO 2.1 D1 A3_Plot7.bmp]

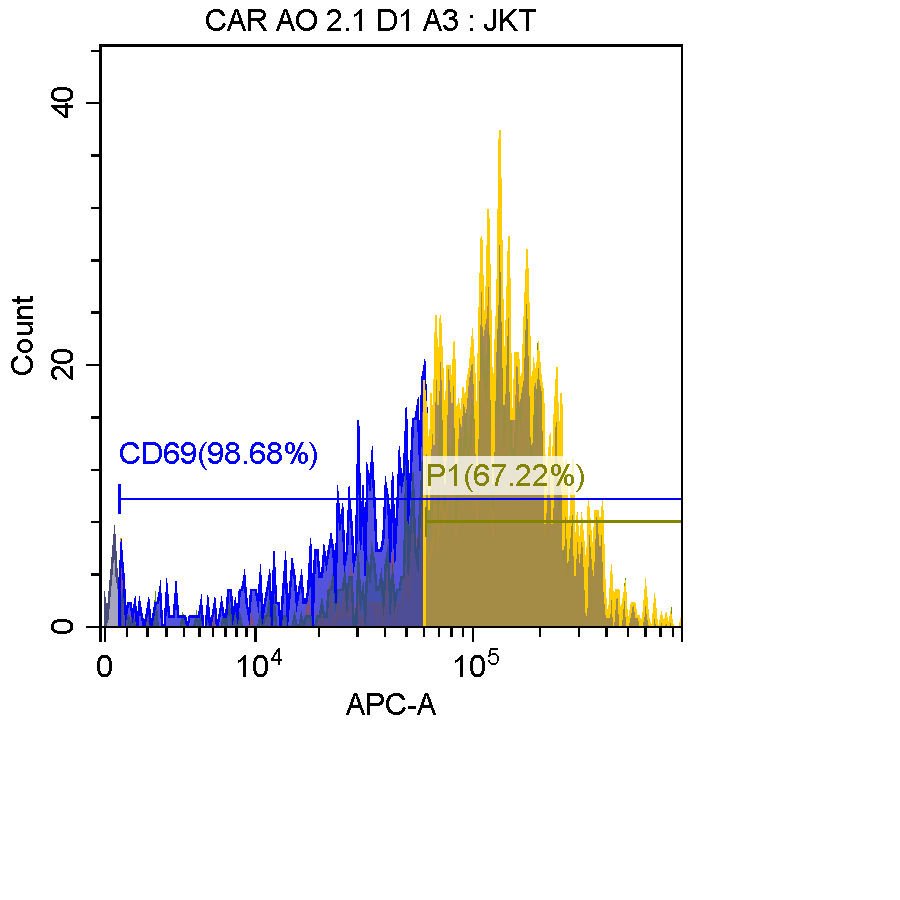

Supplement: Supplementary file 1 [file ijms-24-07641-s001.zip › Cocultures/CAR AO 2.1 D1 A3_Plot8.bmp]

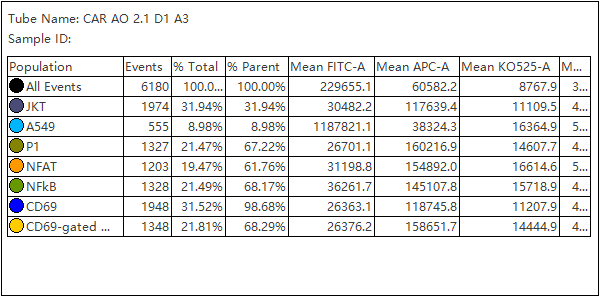

Supplement: Supplementary file 1 [file ijms-24-07641-s001.zip › Cocultures/CAR AO 2.1 D1 A3_Statistics1.bmp]

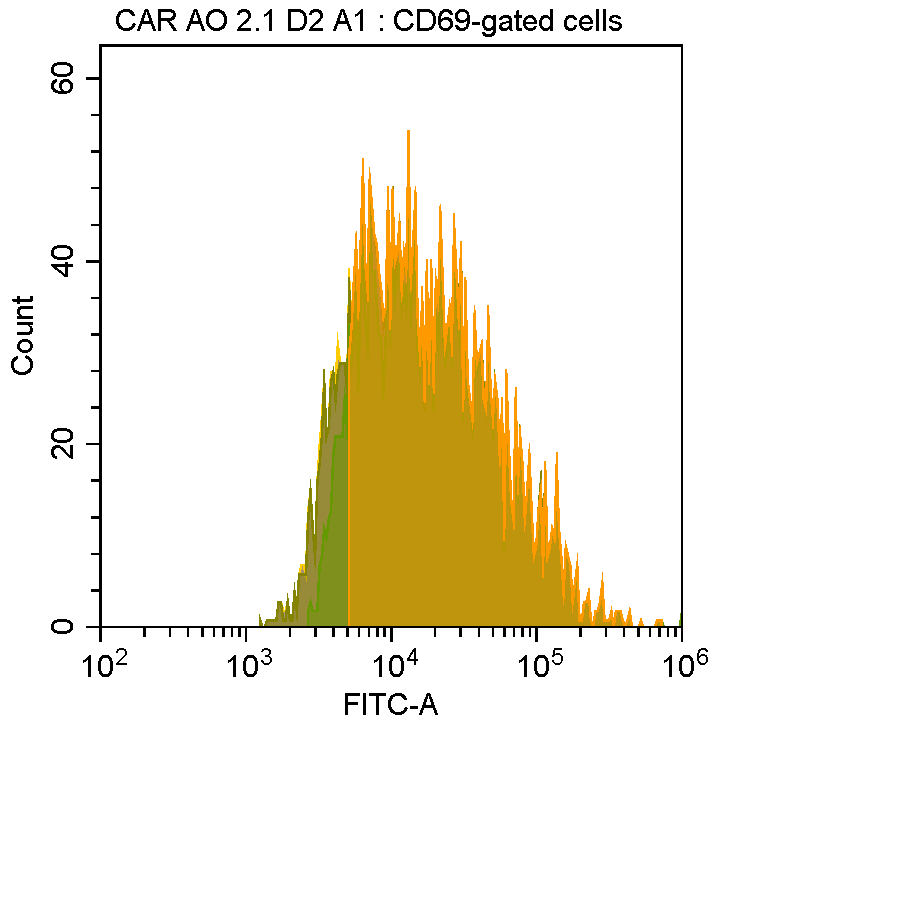

Supplement: Supplementary file 1 [file ijms-24-07641-s001.zip › Cocultures/CAR AO 2.1 D2 A1_Plot1.bmp]

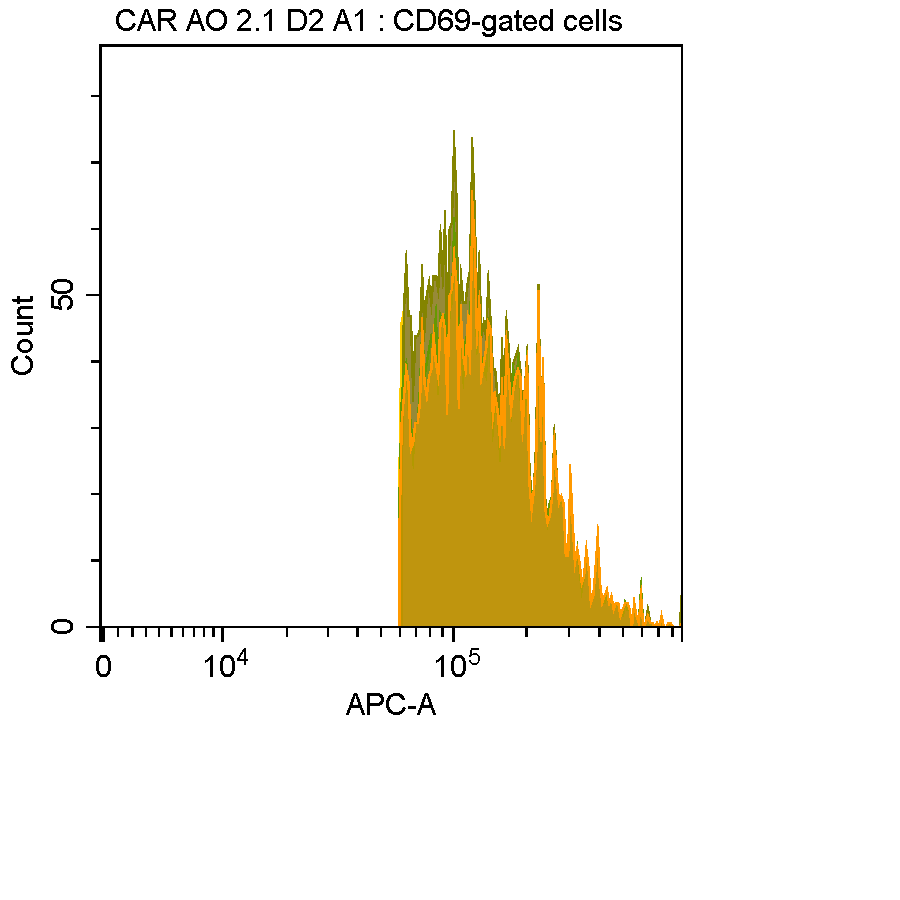

Supplement: Supplementary file 1 [file ijms-24-07641-s001.zip › Cocultures/CAR AO 2.1 D2 A1_Plot2.bmp]

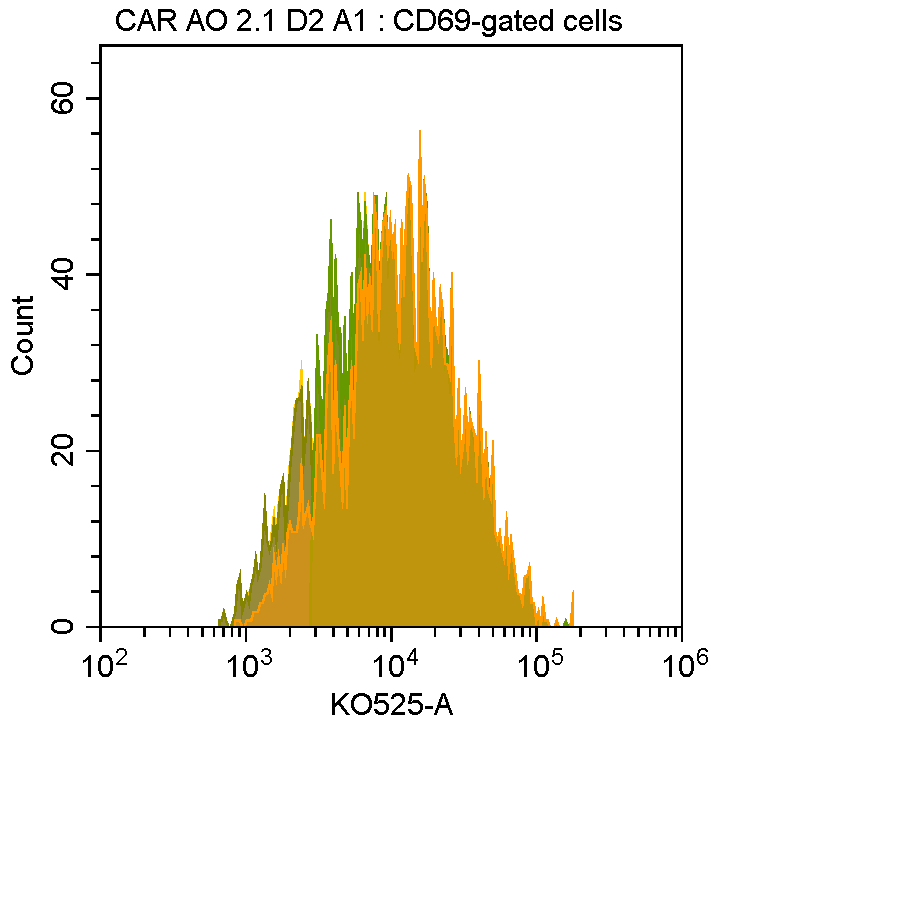

Supplement: Supplementary file 1 [file ijms-24-07641-s001.zip › Cocultures/CAR AO 2.1 D2 A1_Plot3.bmp]

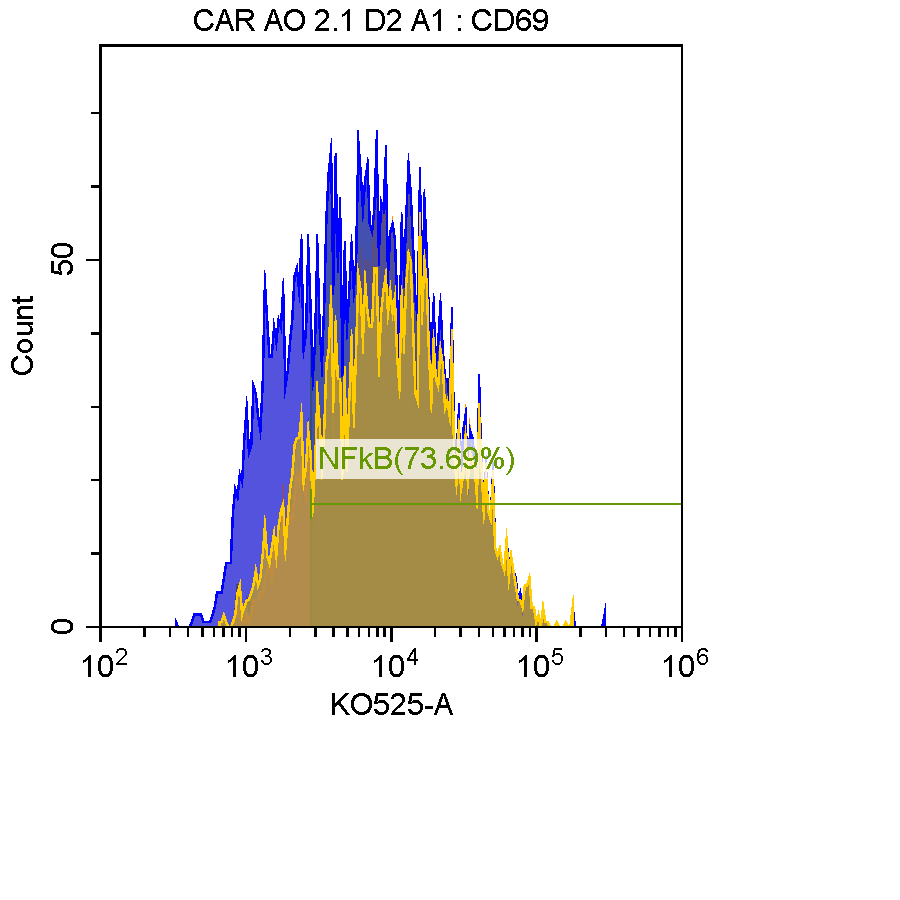

Supplement: Supplementary file 1 [file ijms-24-07641-s001.zip › Cocultures/CAR AO 2.1 D2 A1_Plot4.bmp]

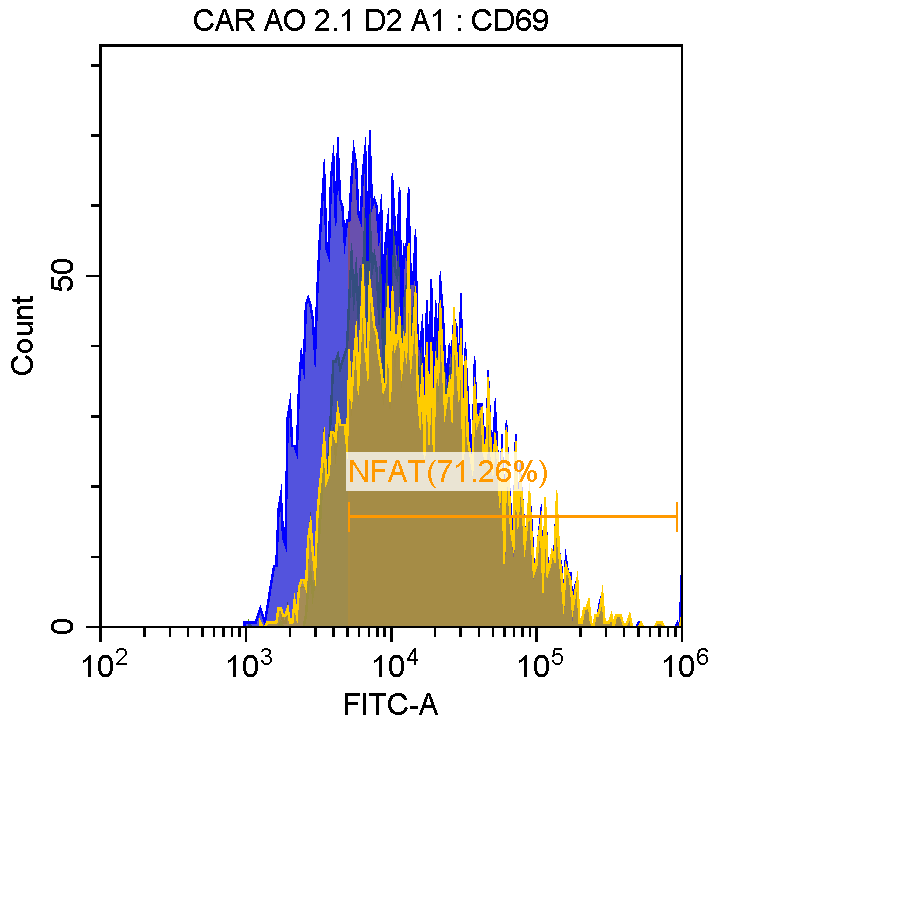

Supplement: Supplementary file 1 [file ijms-24-07641-s001.zip › Cocultures/CAR AO 2.1 D2 A1_Plot5.bmp]

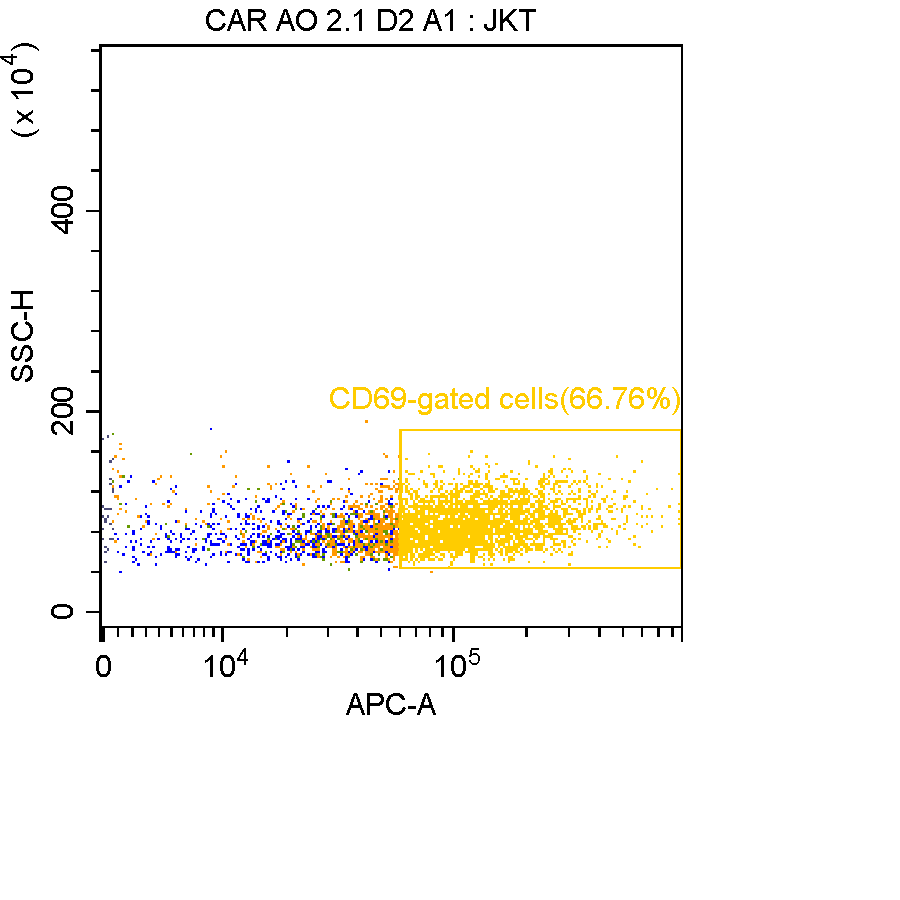

Supplement: Supplementary file 1 [file ijms-24-07641-s001.zip › Cocultures/CAR AO 2.1 D2 A1_Plot6.bmp]

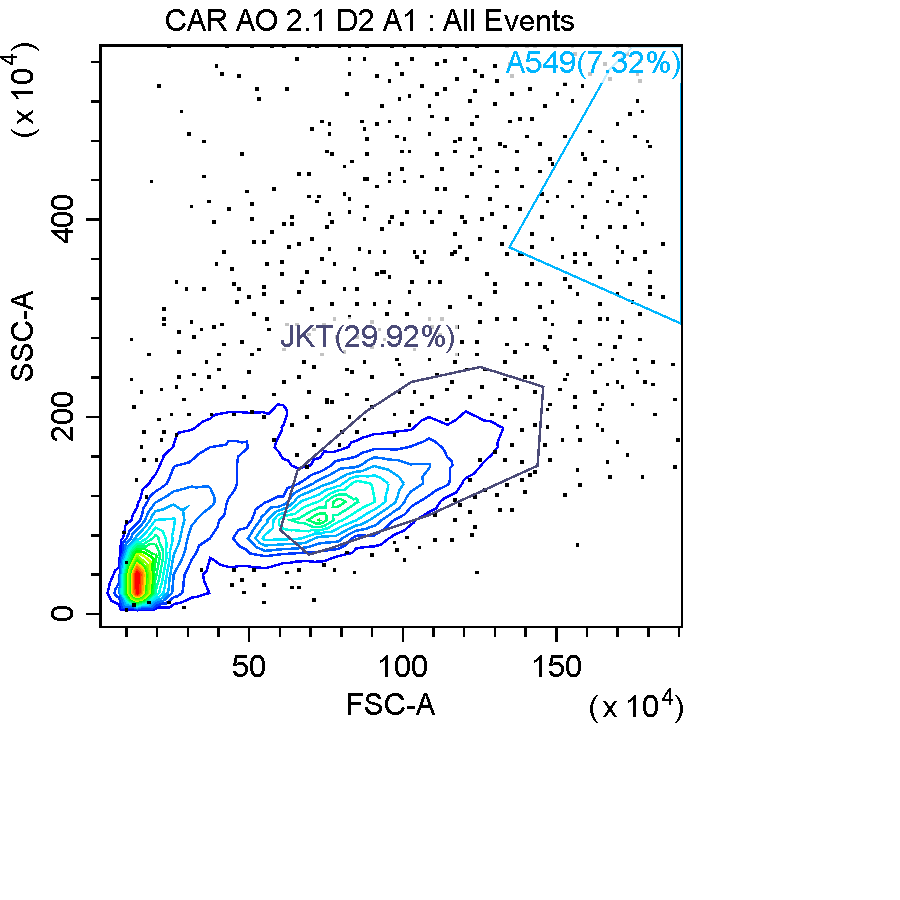

Supplement: Supplementary file 1 [file ijms-24-07641-s001.zip › Cocultures/CAR AO 2.1 D2 A1_Plot7.bmp]

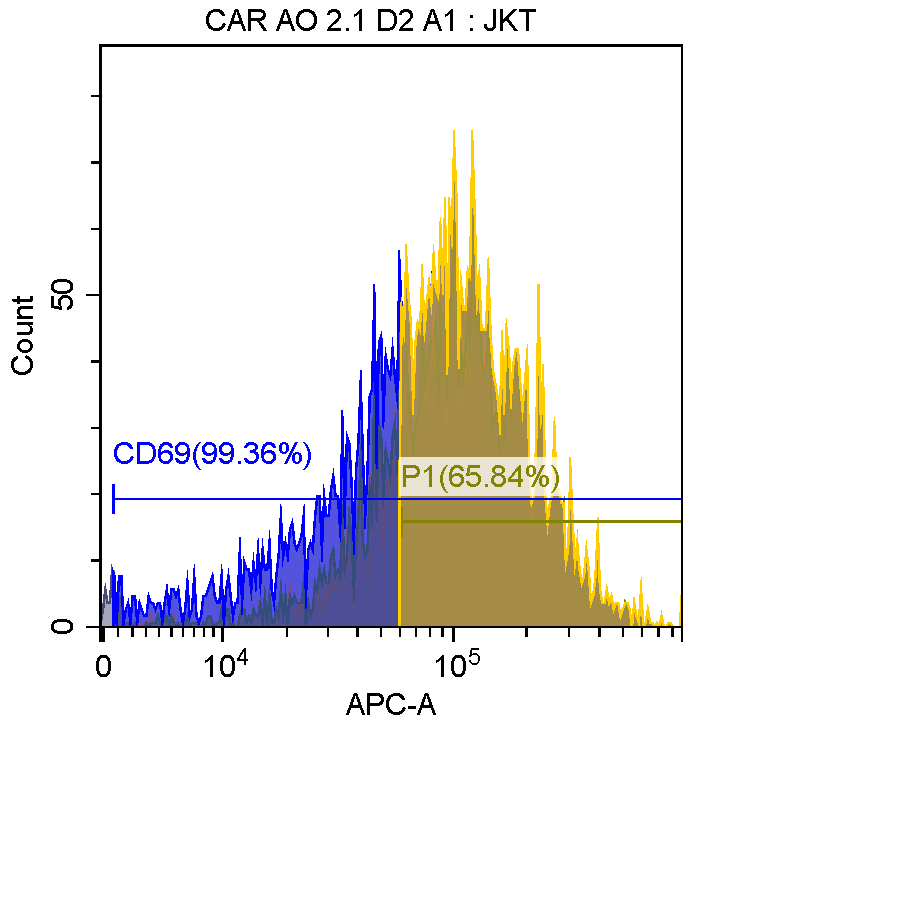

Supplement: Supplementary file 1 [file ijms-24-07641-s001.zip › Cocultures/CAR AO 2.1 D2 A1_Plot8.bmp]

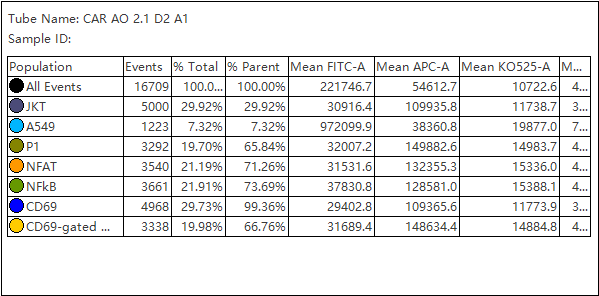

Supplement: Supplementary file 1 [file ijms-24-07641-s001.zip › Cocultures/CAR AO 2.1 D2 A1_Statistics1.bmp]

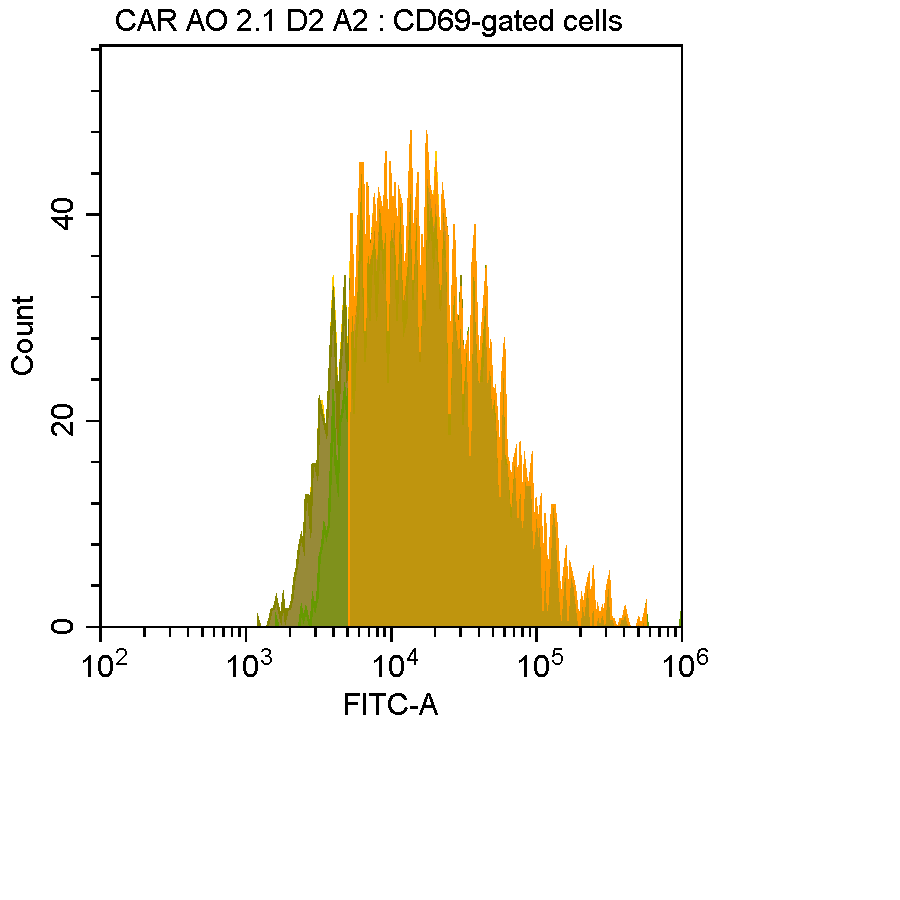

Supplement: Supplementary file 1 [file ijms-24-07641-s001.zip › Cocultures/CAR AO 2.1 D2 A2_Plot1.bmp]

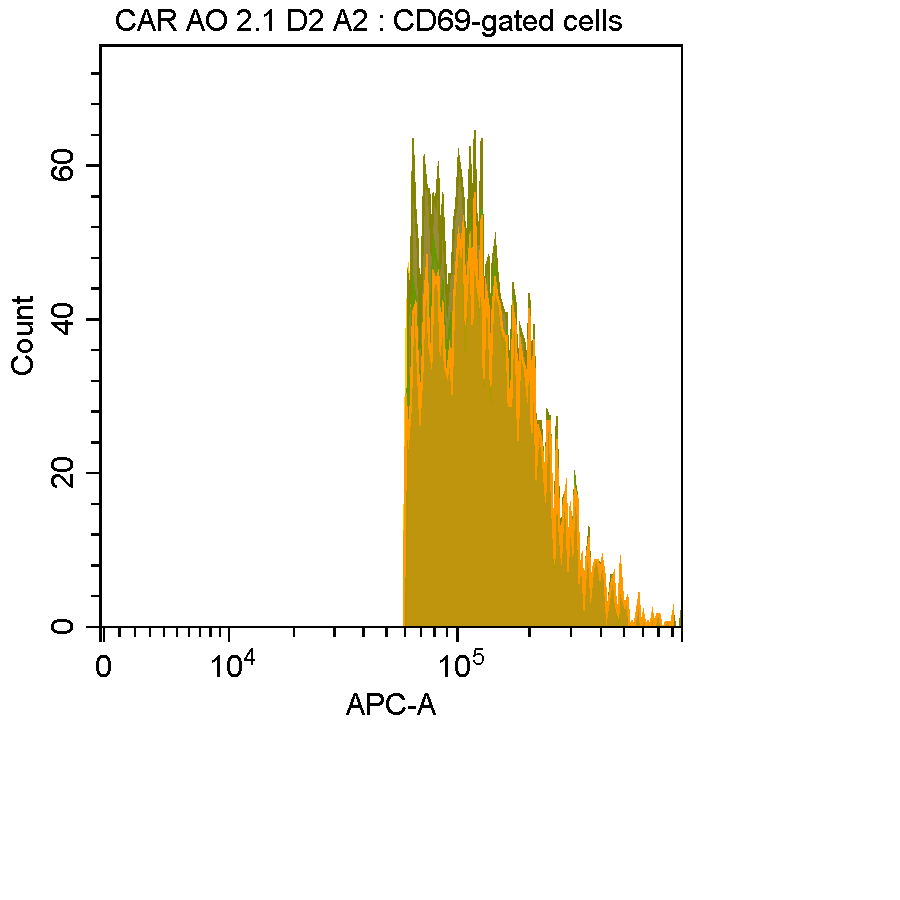

Supplement: Supplementary file 1 [file ijms-24-07641-s001.zip › Cocultures/CAR AO 2.1 D2 A2_Plot2.bmp]

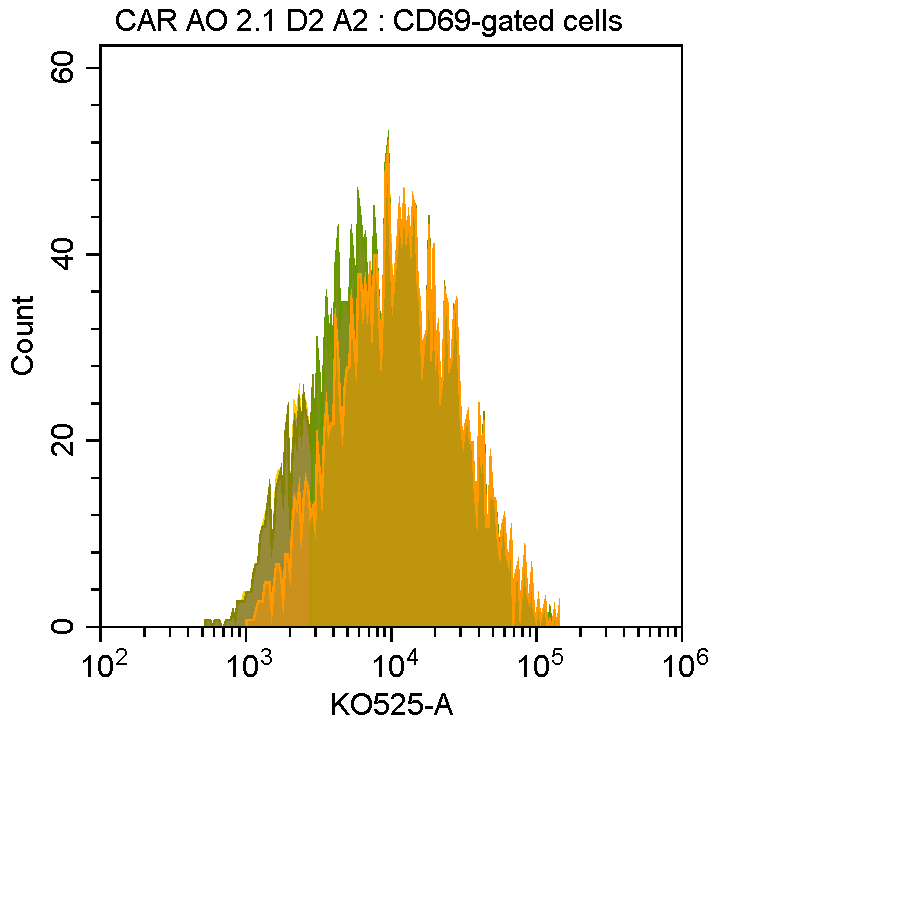

Supplement: Supplementary file 1 [file ijms-24-07641-s001.zip › Cocultures/CAR AO 2.1 D2 A2_Plot3.bmp]

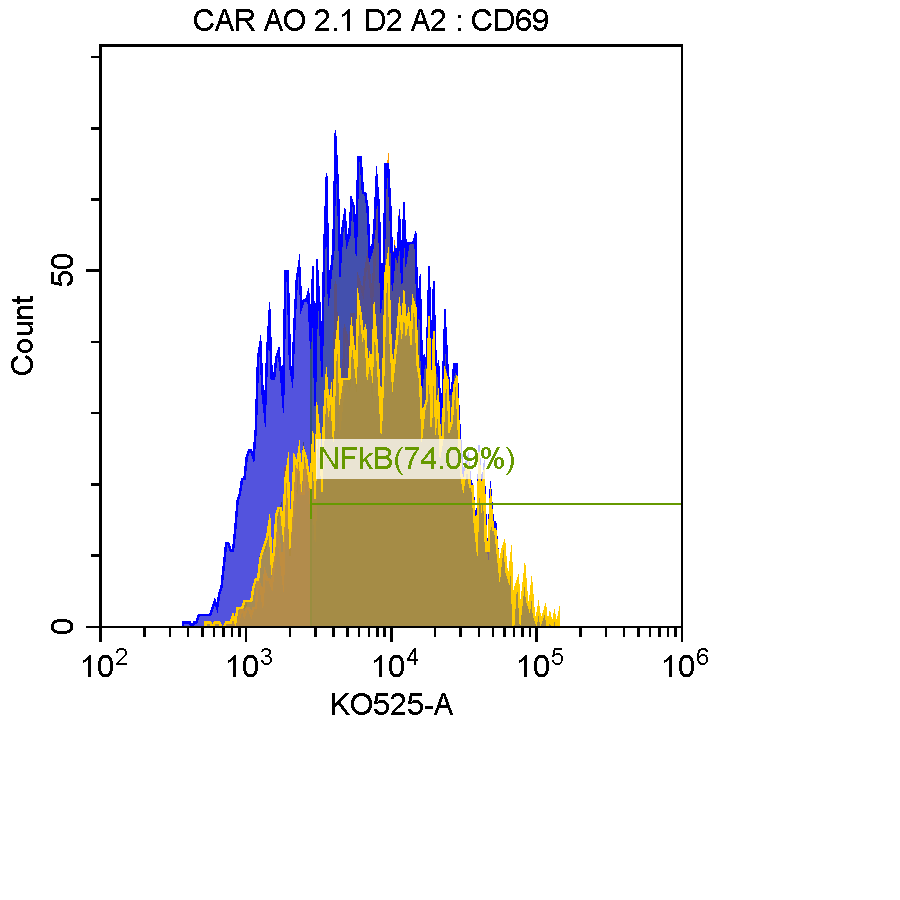

Supplement: Supplementary file 1 [file ijms-24-07641-s001.zip › Cocultures/CAR AO 2.1 D2 A2_Plot4.bmp]

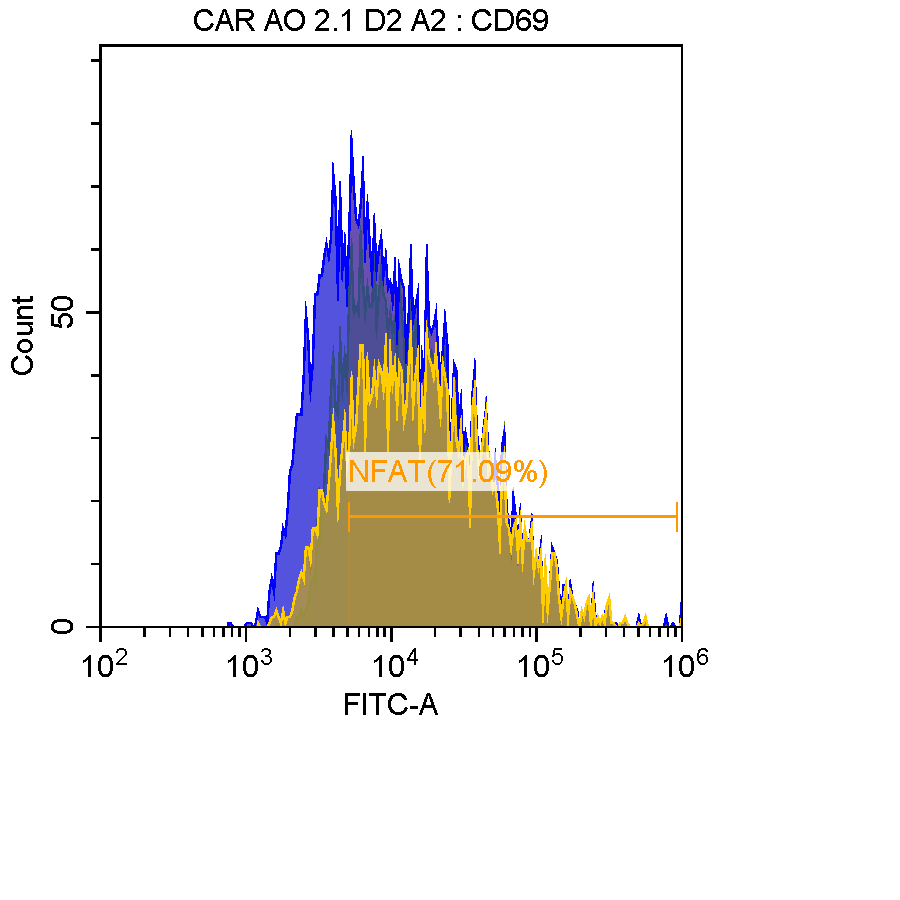

Supplement: Supplementary file 1 [file ijms-24-07641-s001.zip › Cocultures/CAR AO 2.1 D2 A2_Plot5.bmp]

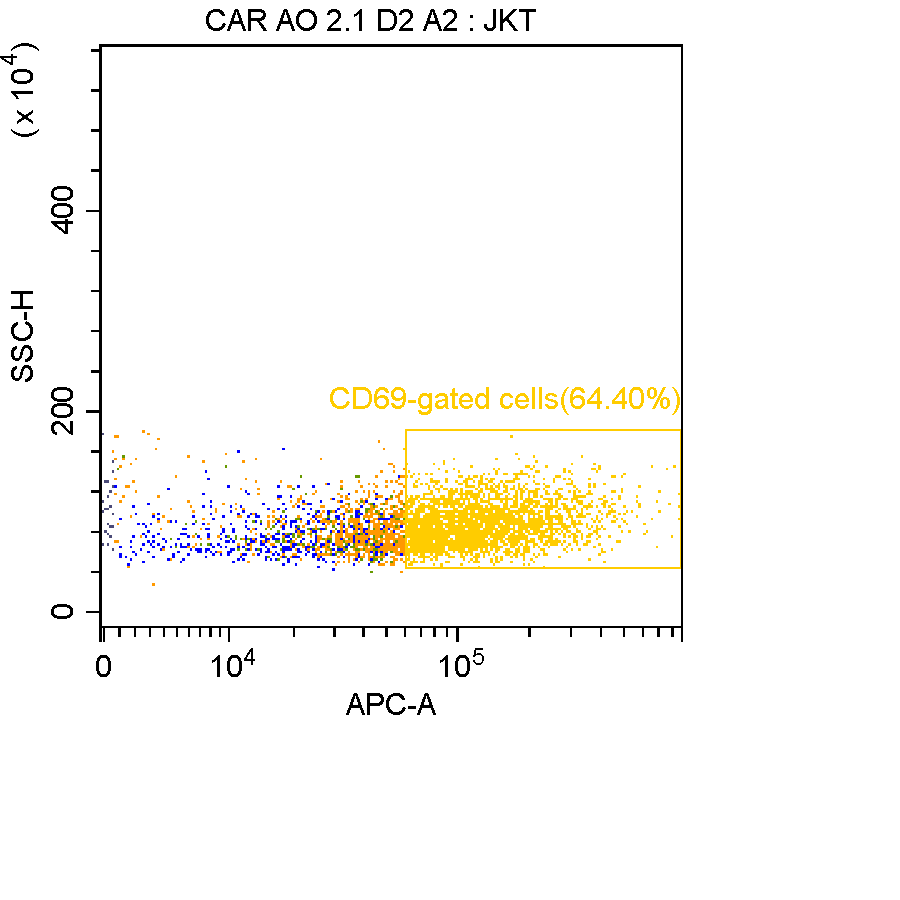

Supplement: Supplementary file 1 [file ijms-24-07641-s001.zip › Cocultures/CAR AO 2.1 D2 A2_Plot6.bmp]

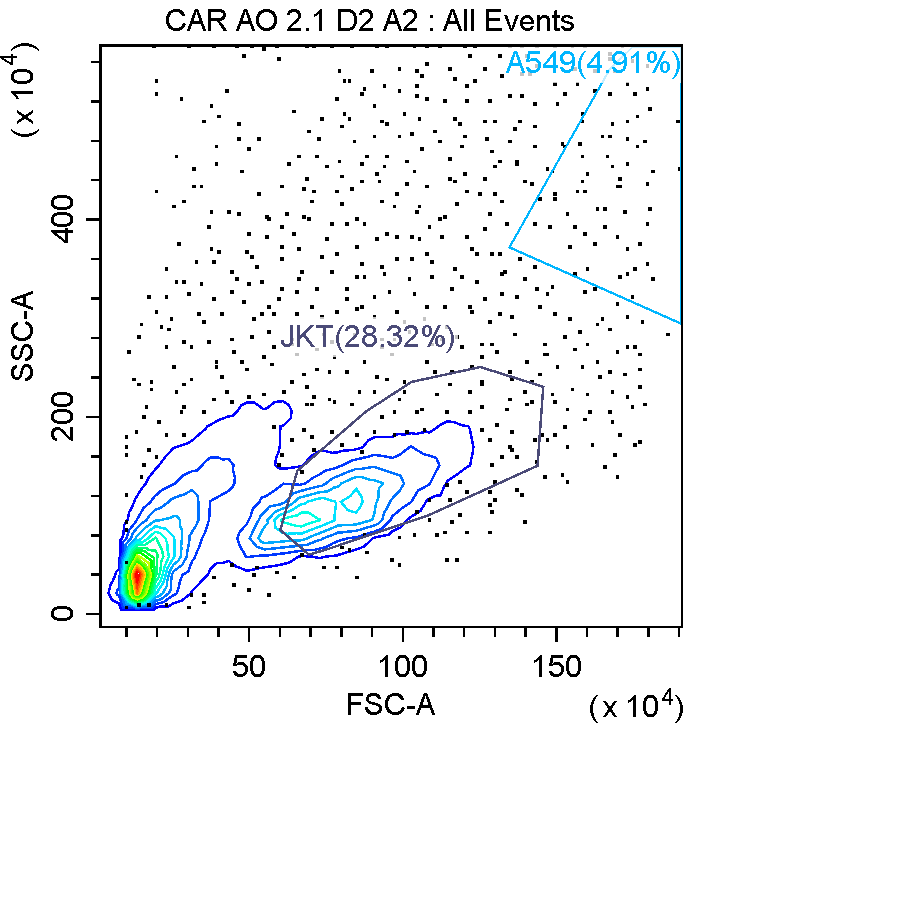

Supplement: Supplementary file 1 [file ijms-24-07641-s001.zip › Cocultures/CAR AO 2.1 D2 A2_Plot7.bmp]

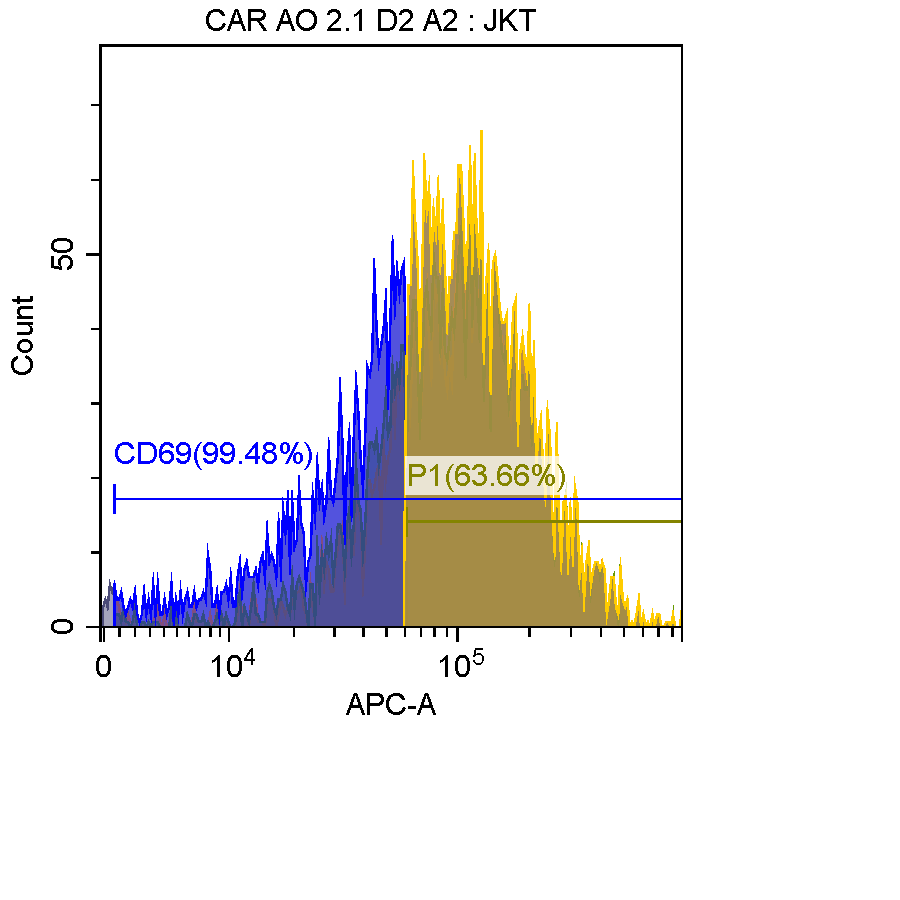

Supplement: Supplementary file 1 [file ijms-24-07641-s001.zip › Cocultures/CAR AO 2.1 D2 A2_Plot8.bmp]

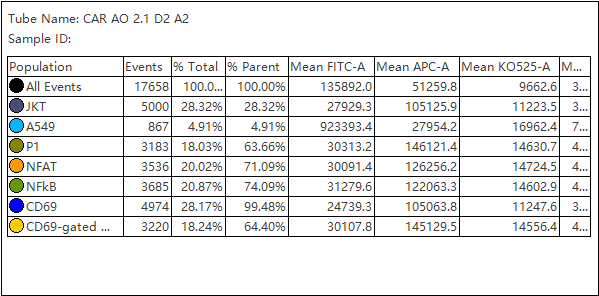

Supplement: Supplementary file 1 [file ijms-24-07641-s001.zip › Cocultures/CAR AO 2.1 D2 A2_Statistics1.bmp]

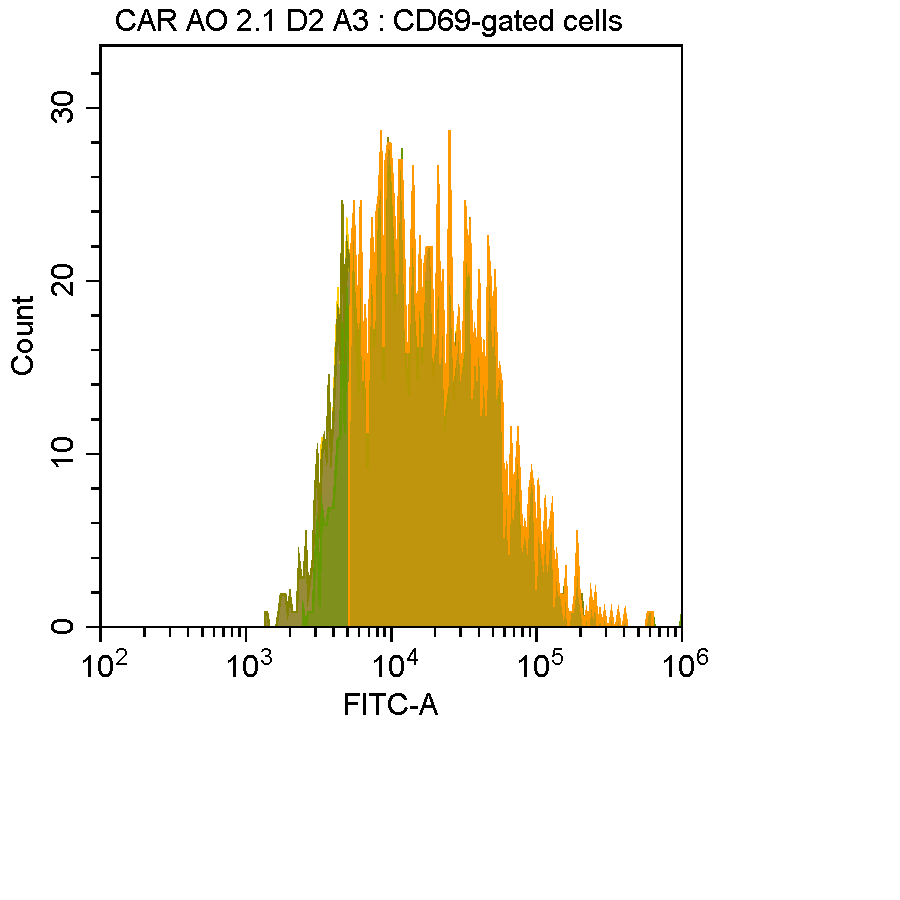

Supplement: Supplementary file 1 [file ijms-24-07641-s001.zip › Cocultures/CAR AO 2.1 D2 A3_Plot1.bmp]

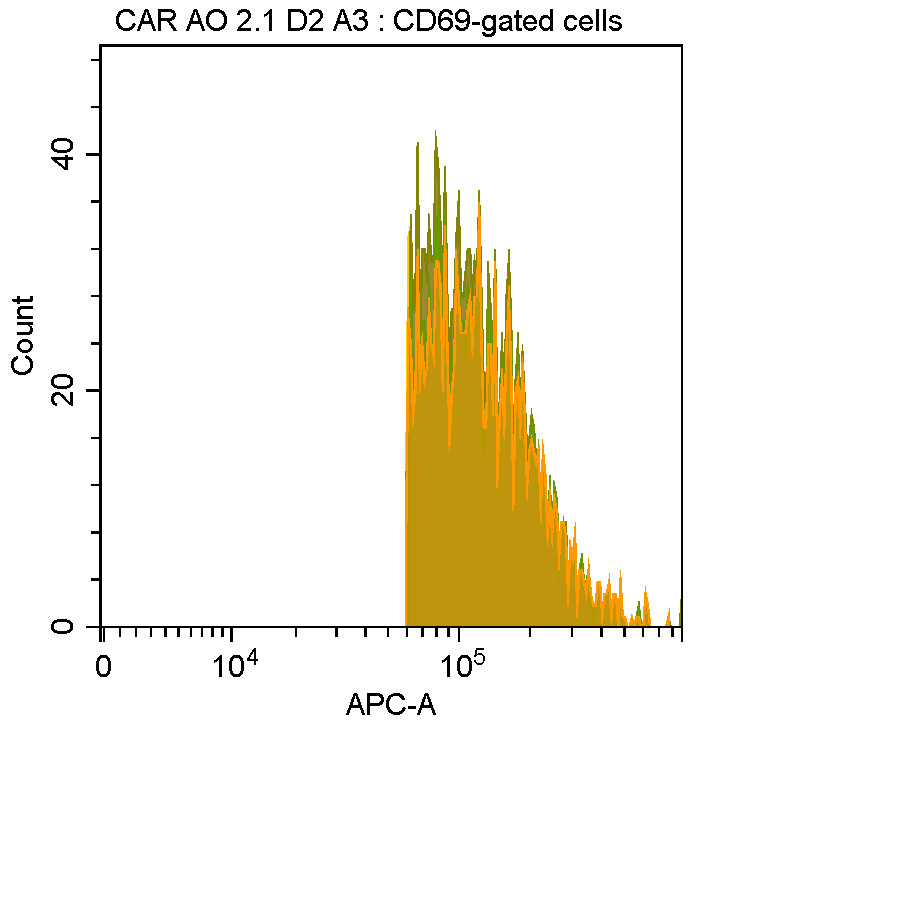

Supplement: Supplementary file 1 [file ijms-24-07641-s001.zip › Cocultures/CAR AO 2.1 D2 A3_Plot2.bmp]
